# GV-001, An Oral Available Histone Deacetylase 6 Inhibitor for the Treatment of Autosomal Dominant Polycystic Kidney Disease

**DOI:** 10.1021/acs.jmedchem.5c03139

**Published:** 2026-04-06

**Authors:** Sian-Siou Wu, Pei-Yun Hung, Tsung-Yu Yeh, Po-Jui Lin, Ji-Wang Chern, Chao-Wu Yu

**Affiliations:** † School of Pharmacy, College of Medicine, 33561 National Taiwan University, Taipei 100, Taiwan; ‡ Gilva Therapeutics Co., Ltd., Taipei 100, Taiwan

## Abstract

Autosomal dominant polycystic kidney disease (ADPKD) is a rare genetic disorder characterized by progressive cyst formation. Emerging evidence suggests that histone deacetylase 6 (HDAC6) plays a pivotal role in the regulation of disease progression. In this study, a series of quinazoli-4-one derivatives were designed and synthesized, among which compound **6a** (GV-001) exhibited superior potency and selectivity in both enzyme inhibition (IC_50_ = 1.18 nM) and immunoblotting assays. Subsequent analysis using the BioMAP fibrosis panel and an *in vitro* human kidney cyst model revealed that GV-001 mitigates inflammatory and fibrotic markers, such as sIL-6 and Collagen I, while effectively suppressing cyst growth. Additionally, GV-001 displayed favorable oral bioavailability (36%) and kidney exposure (AUC_(0–*t*)_: 1790 ng·h/mL) in rats, whereas oral administration produced significant therapeutic efficacy in an ADPKD mouse model, along with upregulation of PC1 expression. These results support GV-001 as a promising and selective HDAC6 inhibitor for the treatment of ADPKD.

## Graphical abstract

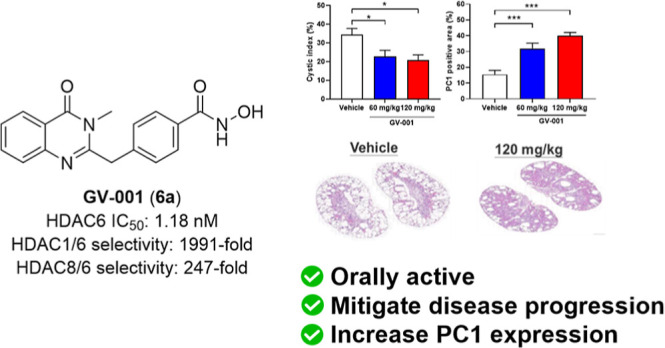

## Introduction

Autosomal dominant polycystic kidney disease (ADPKD) is a chronic kidney disease characterized by bilateral kidney enlargement containing fluid-filled cysts. As a predominant form of hereditary disorder (with a prevalence between 1 in 400 to 1 in 1000),^1^ ADPKD is mainly arisen from single genetic mutations in *PKD1* (78% of cases) and *PKD2* (15%), affecting over 12 million people worldwide.^2,3^ Individuals diagnosed with ADPKD typically exhibit hypertension during early life, and with disease progression, up to 75% of patients eventually develop end-stage renal disease (ESRD) by the age of 70.^4–7^ However, treatment options for this life-threatening disease are still limited and inadequate.

Currently, therapeutic options for patients with ADPKD remain limited. Based on the latest guideline,^8^ V2 receptor antagonist tolvaptan was the only approved treatment, aiming to delay the rapid progression of kidney disease in high-risk patients. Yet its clinical use is constrained by adverse effects, particularly hepatotoxicity. For those with hypertension, angiotensin-converting enzyme inhibitor (ACEi) or angiotensin II receptor blocker (ARB) were recommended, as cyst expansion induces focal renal ischemia, increases renin release, and consequently elevates blood pressure.^9–11^ Since activation of the renin-angiotensin system (RAS) leads to epithelial proliferation and cyst growth, pharmacological inhibition of RAS may provide therapeutic benefits. However, neither tolvaptan nor ACEi/ARBs stops disease progression, making clinical management of ADPKD more challenging.

Polycystin-1 (PC1) and polycystin-2 (PC2) are transmembrane glycoprotein encoded by genes *PKD1* and *PKD2*. Both polycystins, localized to the primary cilium, are essential for regulating intracellular signaling and tubular development in renal cells. Aberrant function of polycystins can disrupt intracellular calcium, cyclic adenosine monophosphate (cAMP), and RAS-RAF-ERK signaling pathway, thereby promoting cyst growth and abnormal cell proliferation.^1,12–14^ Progressive cyst formation and expansion lead to kidney enlargement, which damages normally functioning nephrons and ultimately results in kidney insufficiency.^2^ Additionally, since levels of functional polycystins have been shown to correlate with disease severity, it is speculated that cysts develop once polycystin levels drop below a critical threshold.^15–18^ This implicates polycystins as prognostic markers for evaluating disease progression as well as therapeutic response.

Although the complex pathophysiology of ADPKD complicates therapeutic development, ongoing research has identified potential treatment strategies. Nonetheless, it was not until around the 2010s that the role of HDAC6 in ADPKD was established. HDAC6 is a microtubule-associated deacetylase that belongs to the class IIb HDAC family.^19,20^ Unlike other HDAC isoforms, HDAC6 is predominantly localized in the cytoplasm, where it deacetylates nonhistone protein substrates (e.g., α-tubulin, cortactin, peroxiredoxins, heat shock protein 90, and other chaperone proteins), thereby influencing diverse cellular pathways, including cell migration, apoptosis, protein degradation, cell–cell interaction, etc.^21–23^ While HDAC6 has been shown to exhibit increased expression and activity in ADPKD,^24^ emerging studies suggest that it promotes cyst growth by upregulating intracellular cAMP levels and sustaining EGFR activation.^24–27^ Inhibition of HDACs, particularly HDAC6, by trichostatin A^28^ or selective HDAC6 inhibitors such as ACY-1215,^29^ tubastatin A,^27^ and tubacin^30^ (Figure 1) has been shown to ameliorate kidney cyst formation in both *in vitro* and *in vivo* models. A recent study revealed that a benzothiazole derivative with modest selectivity toward HDAC6 inhibited cyst growth in embryonic kidney and ADPKD mouse models.^31^ Furthermore, it was found that HDAC6 participated in renal fibrosis, which contributes to chronic kidney disease in ADPKD patients. Accumulation of extracellular matrix (ECM) proteins and fibroblast proliferation account for the major cause of renal fibrosis. Overexpression of HDAC6 promotes renal fibrosis through TGF-β-SMAD3 signaling cascade, accelerating epithelial–mesenchymal transition (EMT) and ECM protein accumulation.^25,32^ Altogether, inhibition of HDAC6 seems to offer promising therapeutic effects on ADPKD, which inspired us to develop a small-molecule candidate for effective intervention in patients.

**Figure 1:**
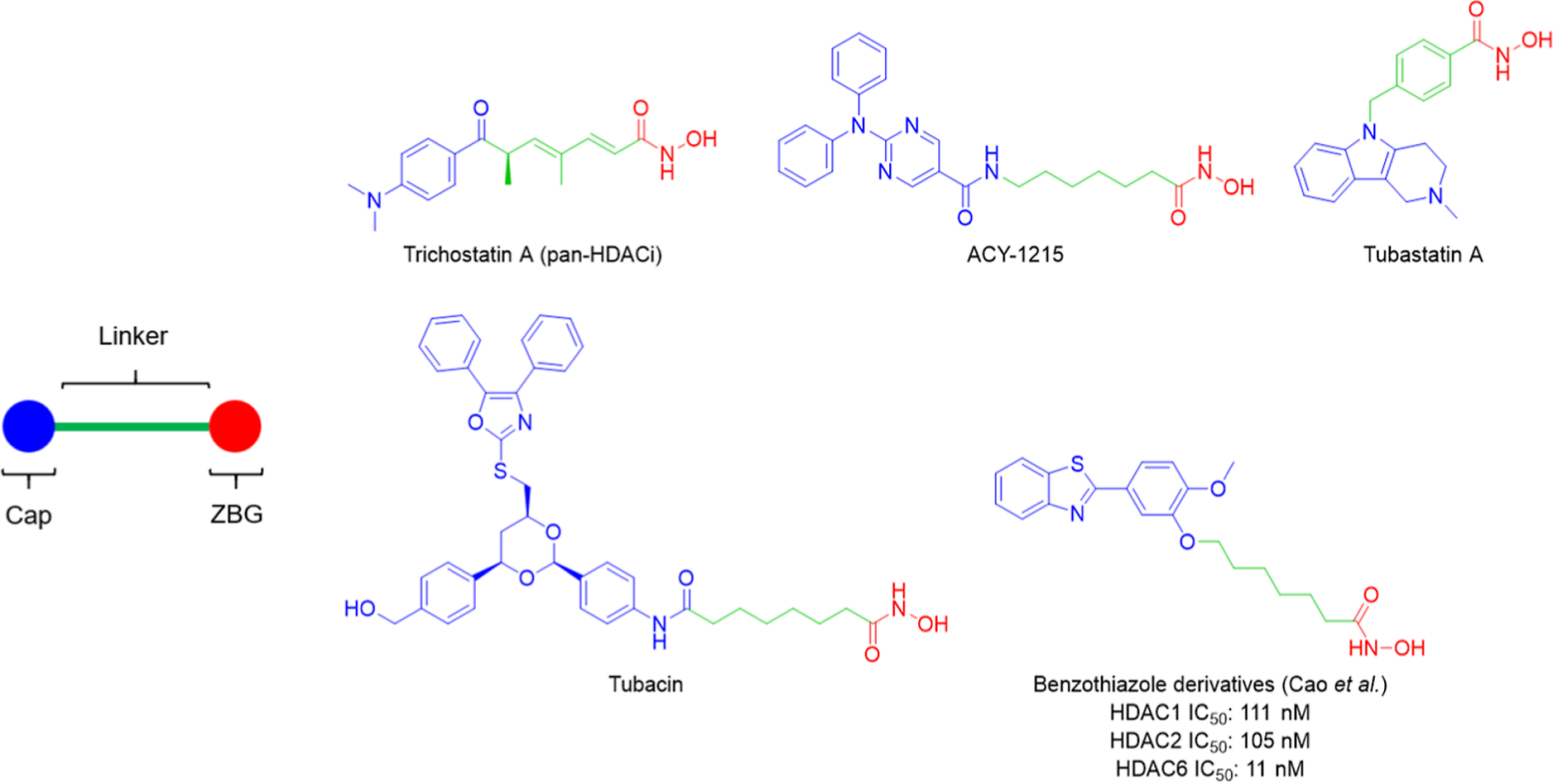
pan-HDAC and HDAC6 inhibitors that have been used to study ADPKD.

Generally, HDAC inhibitors shared a representative pharmacophore model, consisting of three key elements: a cap, a linker, and a zinc binding group (ZBG). Previously, our group has published a series of selective HDAC6 inhibitors derived from the quinazolin-4-one (**1**)^33^ and the quinazolin-2,4-dione (**2**)^34^ cap structures (Figure 2). Based on these preceding structure–activity relationship (SAR) studies, we designed and synthesized a novel set of HDAC6 inhibitors for therapeutic investigation of ADPKD. Evaluation of the BioMAP fibrosis panel were conducted to support the antifibrotic activity of compound **6a** (GV-001). The cellular activities of compounds **6a** and **6c** were compared in human ADPKD cyst models, in which both preventive and reduction assays were conducted. Furthermore, pharmacokinetic (PK) studies were performed to assess oral bioavailability in rats and mice. Finally, the therapeutic efficacy of GV-001 was validated in a transgenic ADPKD mouse model.

**Figure 2:**
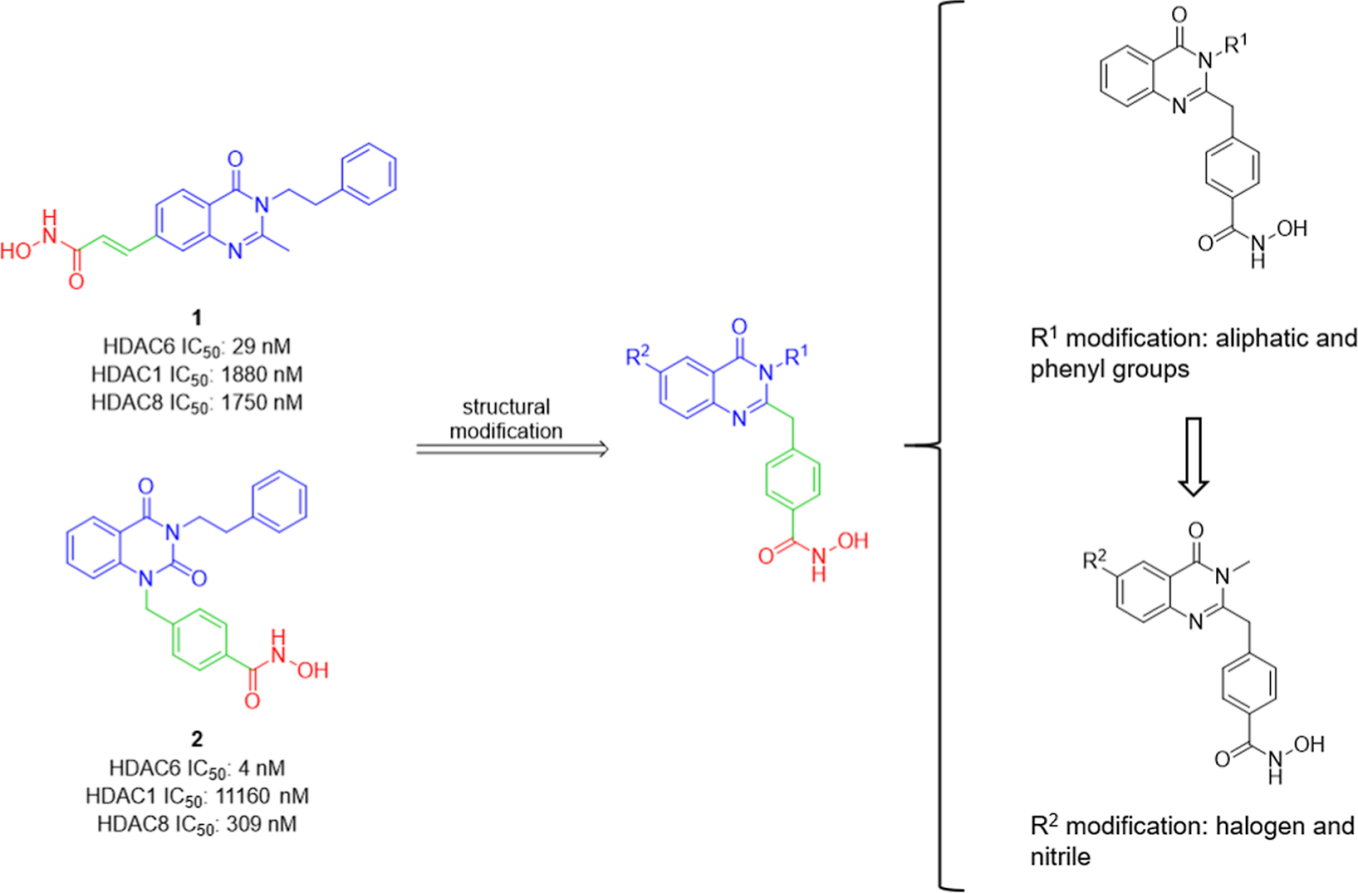
Rational drug design of quinazolin-4-one-based highly selective HDAC6 inhibitors.

## Results and Discussions

### Synthesis of 2-Substituted Quinazolin-4-one Analogs

The four-step synthetic routes for compounds **6a**–**m** were shown in Scheme 1. Briefly, the commercially available compounds **3a**–**l** were treated with 4-bromophenyl acetic acid and triphenyl phosphite, followed by the addition of substituted primary amines to afford quinazolin-4­(*3H*)-one intermediates **4a**–**l**. Following palladium-catalyzed carbonylation of compounds **4** **a–l** with Pd-Xantphos-Mo­(CO)_6_ system produced ester intermediates **5a**–**l** under basic condition. Finally, these ester intermediates were reacted with freshly prepared NH_2_OH in the presence of NaOH in methanol to yield the final products **6a**–**l**.

**Scheme 1:**
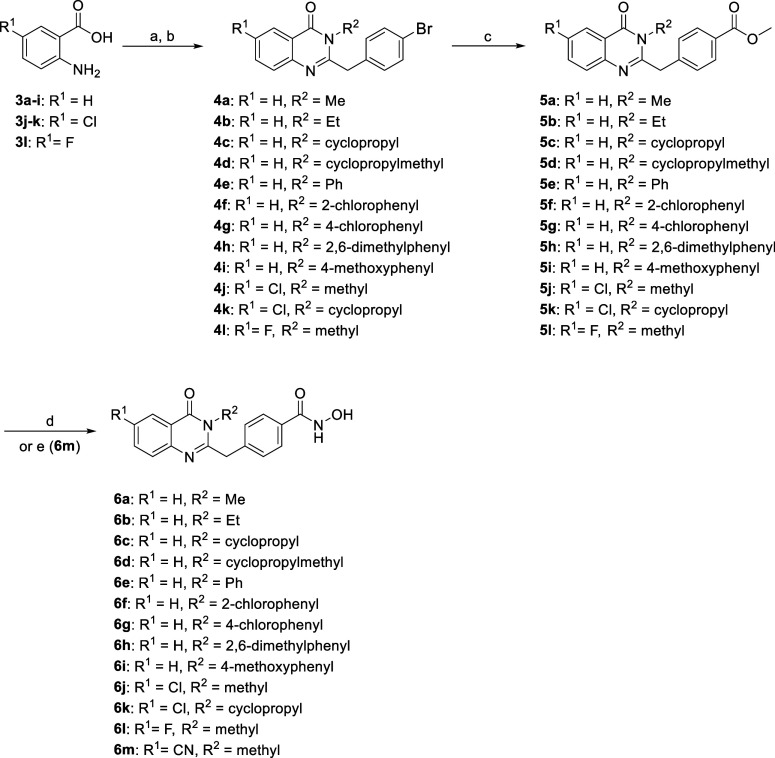
Synthesis of Compounds **6a**–**6m** ^a^

a Reagents and conditions: (a) 4-bromophenyl acetic acid, triphenyl phosphite, pyridine, μwave, 250 W, 130 °C, 15 min; (b) substituted amine, μwave, 250 W, 130 °C, 10 min; (c) Mo­(CO)_6_, Pd­(OAc)_2_, Xantphos, DMAP, DIPEA, DMAC/MeOH (1:1), reflux, 16 h or μwave, 150 W, reflux, 1 h; (d) 2 M NH_2_OH in MeOH, 0 °C to rt, 3 h; (e) (i) NaCN, NiBr_2_, NMP, μwave, 120 W, 200 °C, 10 min; (ii) NH_2_OBn·HCl, EDCI·HCl, HOBt hydrate, DIPEA, DMF, rt, 2 h; (iii) 1 M BBr_3_ dimethyl sulfide complex in DCM, DCM, 0 °C, 30 min.

For the synthesis of target compound **6m**, the nitrile group was inserted from chloride intermediate **5e** with sodium cyanide and nickel bromide via microwave irradiation to give nitrile intermediate. While most of the nitrile intermediate was found to bear a carboxylic acid instead of the original ester group, the carboxylic acid group was reacted with NH_2_OBn with coupling agents and then treated with BBr_3_ to afford hydroxamic acid **6m**.

### Structure–Activity Relationship

While previously reported HDAC6 inhibitors from our group incorporated zinc-binding groups at positions 1, 6, or 7,^33,34^ efforts to introduce a hydroxamic acid moiety at position 2 have produced a range of highly selective HDAC6 inhibitors. In this study, various substitutions at position 3 was investigated their enzymatic inhibition activities toward HDAC1, 6, and 8 (Table 1). Compounds **6a**–**d** exhibited significantly greater HDAC6 inhibitory activity (IC_50_ = 0.98–11.3 nM) than compounds **6e**–**i** (IC_50_ = 7.72–157 nM), indicating that aliphatic substituents were more active than aromatic ones. With respect to HDAC1 and HDAC8 biochemical values, compound **6a** showed excellent selectivity, with selectivity indices of 1991- and 247-fold, respectively. Replacement of the methyl group with a cyclopropyl moiety provided compound **6c**, which gave rise to even higher selectivity against HDAC1 (4000-fold) and HDAC8 (401-fold). In contrast, the selectivity of **6b** and **6d** were apparently dropped (HDAC1/6:175- to 396-fold; HDAC8/6:70- to 101-fold) upon the introduction of an additional carbon. In terms of aromatic substituents (**6e**–**6i**), most compounds gave much lower selectivity compared to **6a** and **6c**. The exception was the 2,6-dimethyl substituted phenyl **6h**, which exhibited comparable HDAC6 potency (IC_50_ = 7.72 nM) and HDAC8/6 selectivity (181-fold).

**Table 1:**
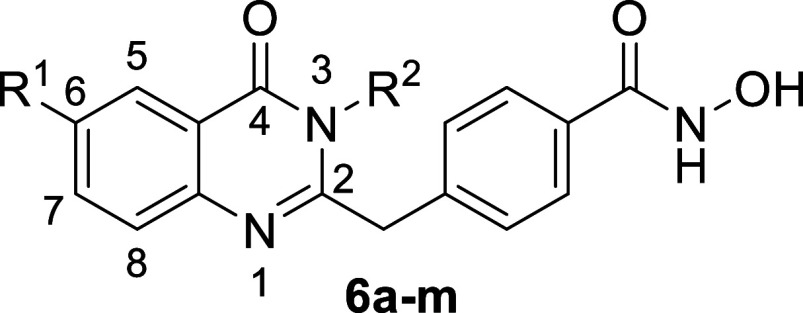
HDAC Biochemical IC_50_ Values and Selectivity Index of Compounds **6a**–**m**

Compd	R^1^	R^2^	HDAC IC_50_ (nM)^a^			selectivity Index HDAC1/6	selectivity Index HDAC8/6	*c* log *P* ^b^
			HDAC1	HDAC6	HDAC8			
**6a (GV-001)**	H	Me	2350	1.18	291	1991	247	0.41
**6b**	H	Et	788	4.51	455	175	101	1.02
**6c**	H	cyclopropyl	3920	0.98	393	4000	401	1.41
**6d**	H	cyclopropylmethyl	4480	11.3	796	396	70	1.61
**6e**	H	phenyl	12,120	73.1	1490	16.6	20	1.64
**6f**	H	2-chlorophenyl	ND	85.8	628	-	7	2.26
**6g**	H	4-chlorophenyl	ND	14.2	697	-	49	2.38
**6h**	H	2,6-dimethylphenyl	ND	7.72	1400	-	181	2.55
**6i**	H	4-methoxyphenyl	12,600	157	ND	80	-	1.74
**6j**	Cl	Me	ND	<1.52	274	-	>180	1.37
**6k**	Cl	cyclopropyl	ND	8.73	440	-	50	2.37
**6l**	F	Me	805	1.12	ND	719	-	0.90
**6m**	CN	Me	989	2.37	ND	417	-	0.35
**Trichostatin A**			1.11	2.87	291	0.39	101	2.40

a Data from Reaction Biology Corporation. Compound and control (trichostatin A) were tested once in 10-dose IC_50_ mode with 3-fold serial dilutions starting at 30 μM.

b cLogP values were calculated via MolSoft at https://www.molsoft.com/.

c ND, not determined.

Additionally, a series of analogs incorporating chloro (**6j**–**k**), fluoro (**6l**), and nitrile (**6m**) groups at R^1^ were designed to reduce hydrophilicity relative to compounds **6a** and **6c**. Despite showing favorable HDAC6 activity (**6j**–**m**, IC_50_ = 1.12–8.73 nM), these analogs were markedly less selective than compounds **6a** and **6c**. Collectively, in the current SAR studies, most compounds demonstrated HDAC6 IC_50_ values below 100 nM, with methyl (**6a**, **6j**, **6l**–**m**) and cyclopropyl (**6c**, **6k**) substituted analogs displaying nanomolar to subnanomolar bioactivity. Moreover, compounds **6a** and **6c** exhibited the highest selectivity against HDAC1 and HDAC8, indicating their promise for further pharmacological studies.

### *In Vitro* α-Tubulin Acetylation Based on Immunoblotting

Given the favorable outcomes in the enzyme inhibitory assays, compounds **6a** and **6c** were subsequently evaluated for their effects on α-tubulin and histone H3 acetylation in HepG2 cells (Figure 3 and S1). Analysis of Ac-α-tubulin expression levels revealed that both **6a** and **6c** inhibited tubulin deacetylation in a dose-dependent manner. Further comparison of the IC_50_ values for Ac-H3 indicated that **6a** exhibited a 24-fold selectivity for Ac-α-tubulin (Ac-α-tubulin: IC_50_ = 0.19 μM; Ac-H3: IC_50_ = 4.46 μM), whereas the selectivity of **6c** was notably lower (Ac-α-tubulin: IC_50_ = 3.64 μM; Ac-H3: IC_50_ = 7.88 μM). These results, which appear paradoxical to the observed enzyme activity, may be attributed to the poor cellular permeability of compound **6c**. Therefore, compound **6a** was identified as the more effective HDAC6 inhibitor, as evidenced by its selective upregulation of acetylated α-tubulin expression.

**Figure 3:**
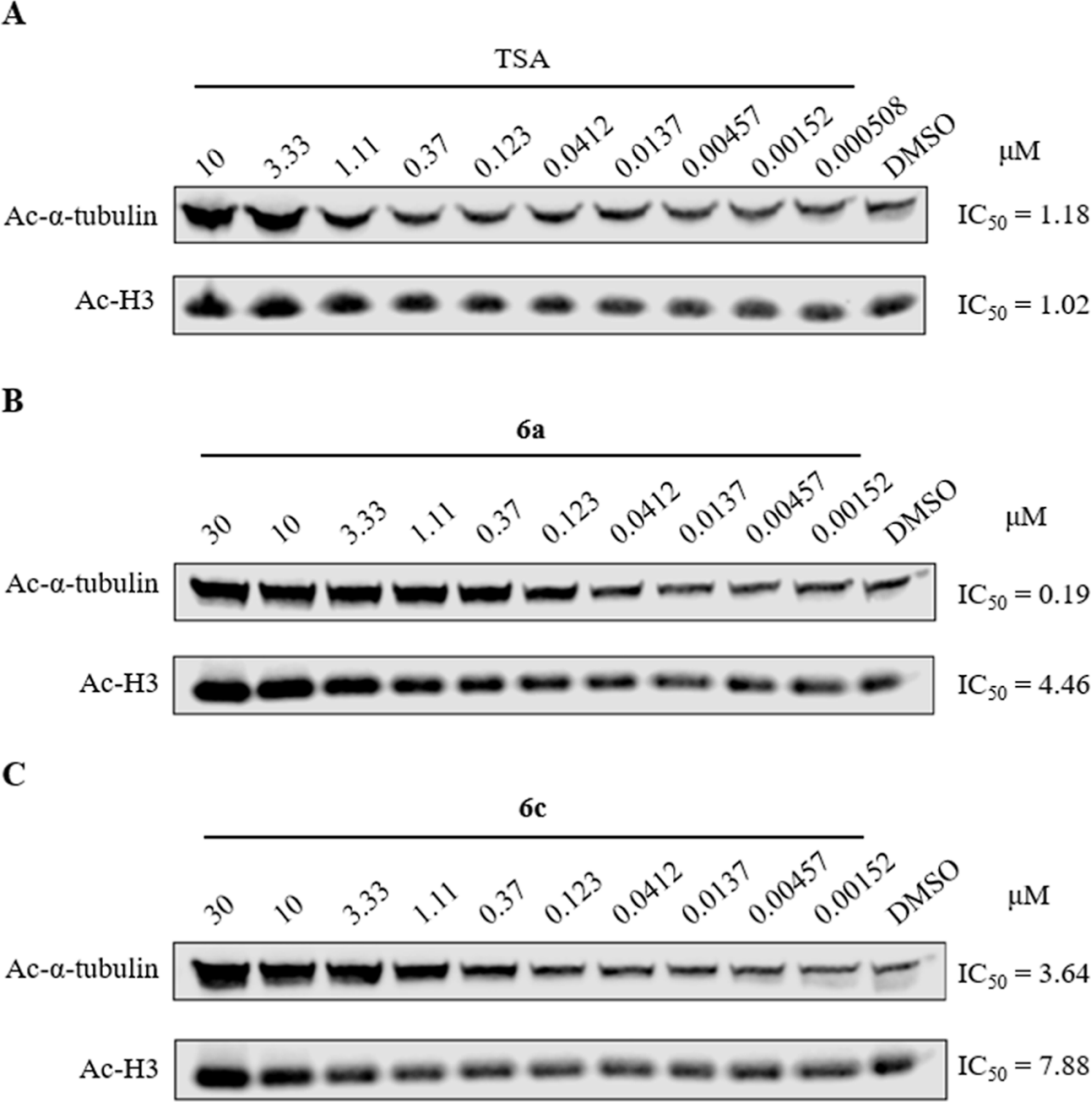
Western blot analysis of Ac-α-tubulin, Ac-H3 in HepG2 cells after 16 h of treatment. (A) Treatment with Trichostatin A (TSA) at 0.000508 to 10 μM or DMSO; (B) Treatment with **6a** at 0.00152 to 30 μM or DMSO; (C) Treatment with **6c** at 0.00152 to 30 μM or DMSO. IC_50_ curves were shown in Figure S1.

### Compound **6a** Modulates Fibrosis Markers in Human Primary Cell-Based Models of Renal Fibrotic Diseases

Next, the phenotypic profiles of selective HDAC6 inhibitor **6a** was evaluated using the BioMAP Fibrosis Panel, which comprises two selected human primary cell-based systems (Figure 4).^35^ These systems were designed to simulate the complex human disease states underlying fibrosis and wound healing, focusing on transforming growth factor β (TGF-β) and tumor necrosis factor α (TNF-α) driven aberrant inflammation related to renal fibrosis diseases. Across a broad panel of biomarker readouts, the BioMAP platform enables the prediction of efficacy and the elucidation of potential mechanisms of action through comparison with the BioMAP Reference Database.

**Figure 4:**
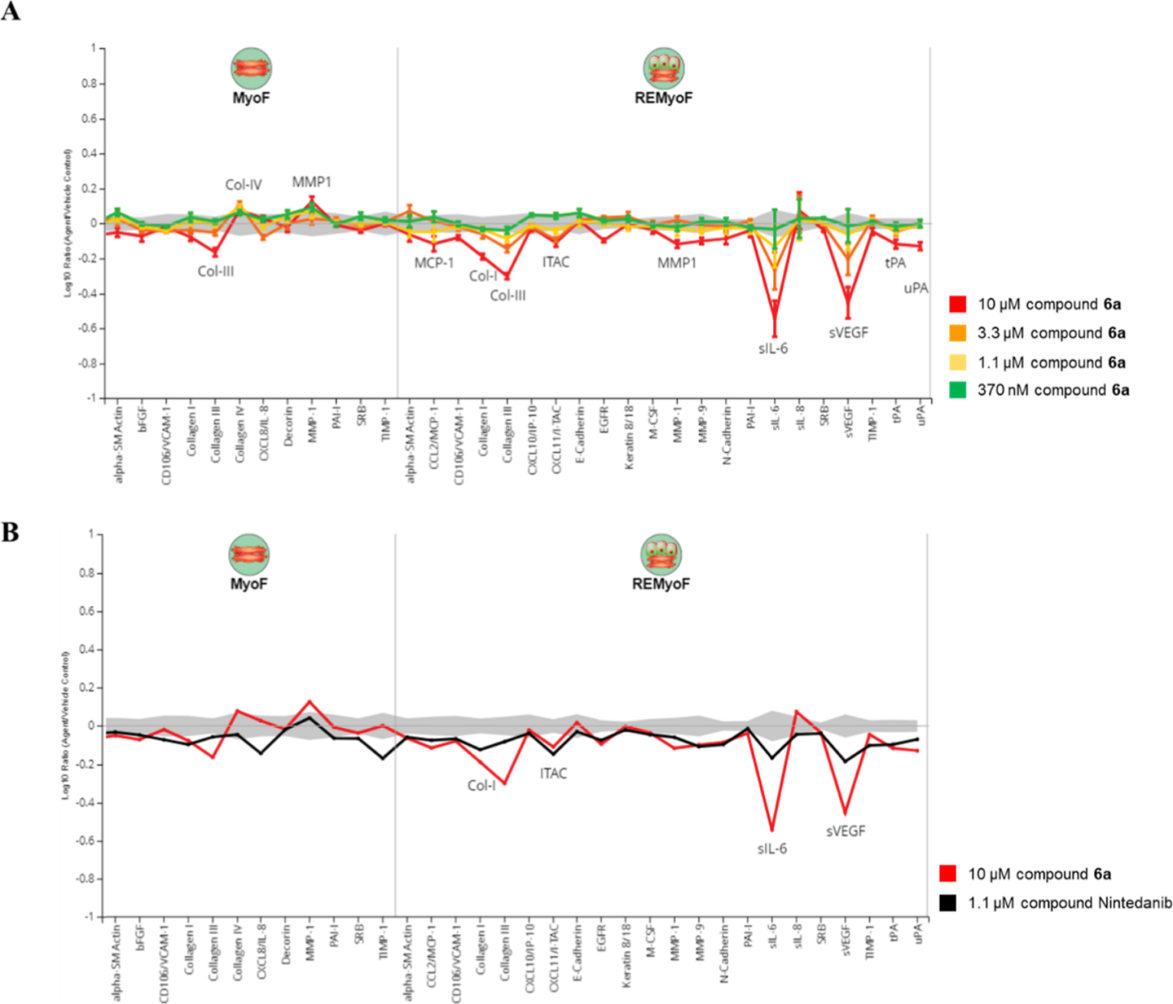
(A) BioMAP profile of **6a** in the Fibrosis Panel. The *X*-axis lists the quantitative protein-based biomarker readouts measured in each system. The *Y*-axis represents a log-transformed ratio of the biomarker readouts for the drug-treated sample (*n* = 3) over vehicle controls (*n* ≥ 6). (B) Overlay of Nintedanib and **6a**. Common biomarker readouts are annotated when the readout for both profiles is outside of the significance envelope with an effect size >20% (|log_10_ ratio| > 0.1). MyoF (modeling fibrosis): human primary lung fibroblasts; REMyoF (modeling kidney fibrotic disease): cocultured with renal proximal tubule epithelial cells.

In this study, four concentrations of **6a** (370 nM, 1.1 μM, 3.3 μM and 10 μM) were evaluated. At a concentration of 10 μM, compound **6a** significantly decreased inflammation-related markers (I-TAC, sIL-6, MCP-1), modulation of fibrosis-related matrix proteins (decreased Collagen I and III; increased Collagen IV; modulated MMP-1), and reduced tissue remodeling/wound healing markers (tPA, uPA, sVEGF) (Figure 4a). No cytotoxicity was observed at any of the tested concentrations, as determined by the sulforhodamine B (SRB) assay following 48 h of incubation. These data suggested compound **6a** is capable of suppressing inflammatory and EMT biomarkers, resulting in reduced production of ECM components.^36–39^ In the MyoF system, compound **6a** also attenuates the levels of Collagen III suggested that it can mitigate ECM deposition without the interaction with epithelial cell.

By comparing the screening profiles with 1.1 μM nintedanib (an approved antifibrotic drug) (Figure 4b), four overlapping biomarker responses were observed in the REMyoF system (Collagen I, I-TAC, sIL-6, and sVEGF), while three differentiating biomarkers were identified (MyoF: IL-8, TIMP-1, MMP-1). These findings indicate that nintedanib and **6a** exhibited both shared and distinct modulatory effects on fibrosis- and inflammation-related pathways. Overall, compound **6a** had greater impacts on key fibrosis-related biomarkers, including sIL-6 and collagens, at noncytotoxic concentrations. HDAC6 inhibition by **6a** attenuated the expression of inflammatory and immunoregulatory chemokines, EMT markers, and ECM components, in which their aberrant expressions are thought to drive the fibrosis.

### Compounds **6a** and **6c** Inhibits the Growth of Human ADPKD Cysts *In Vitro*

Emerging evidence suggests that inhibition of HDAC6 reduces cyst growth, highlighting its therapeutic potential for the treatment of ADPKD.^25,27,30^ To evaluate the effects of selective HDAC6 inhibitors on suppressing cyst growth, an *in vitro* 3D assay model was employed. Conventional 2D culture systems result in monolayer growth of kidney cells and do not support cyst formation; thus, embedding primary renal cells in a biogel matrix facilitates the formation of 3D cystic structures *in vitro*.^40^

In the current assays, cyst viability (CTG), cytotoxicity (LDH), cyst number (CN), total cyst area (TA), and average size per cyst (size/cyst) were calculated following a 14 day culture of drug-treated primary human kidney cells derived from ADPKD patients (Figure 5, S2 and S3). Prevention and reduction assays were performed to evaluate the inhibitory effect of **6a** and **6c** on cyst growth. Table 2 summarizes the IC_50_ values of prevention and reduction experiments. The results indicated that pretreatment or treatment with **6a** or **6c** led to a significant reduction of cyst viability, cyst number, total cyst area, and size/cyst in a concentration-dependent manner. Notably, the preventive treatment exhibited greater efficacy compared to the rescue assay. While both compounds effectively inhibited cyst growth, compound **6a** demonstrated a superior effect in reducing cystogenic metrics. Moreover, cytotoxicity assessed using the lactate dehydrogenase (LDH) assay indicated that both compounds induced cytotoxic effects only at concentrations above 90 μM (Figures S2 and S3), whereas the reference drug ricolinostat (ACY-1215) displayed cytotoxicity at 3 and 10 μM. These results suggest that the *in vitro* efficacy of compounds **6a** and **6b** is independent of cytotoxicity.

**Figure 5:**
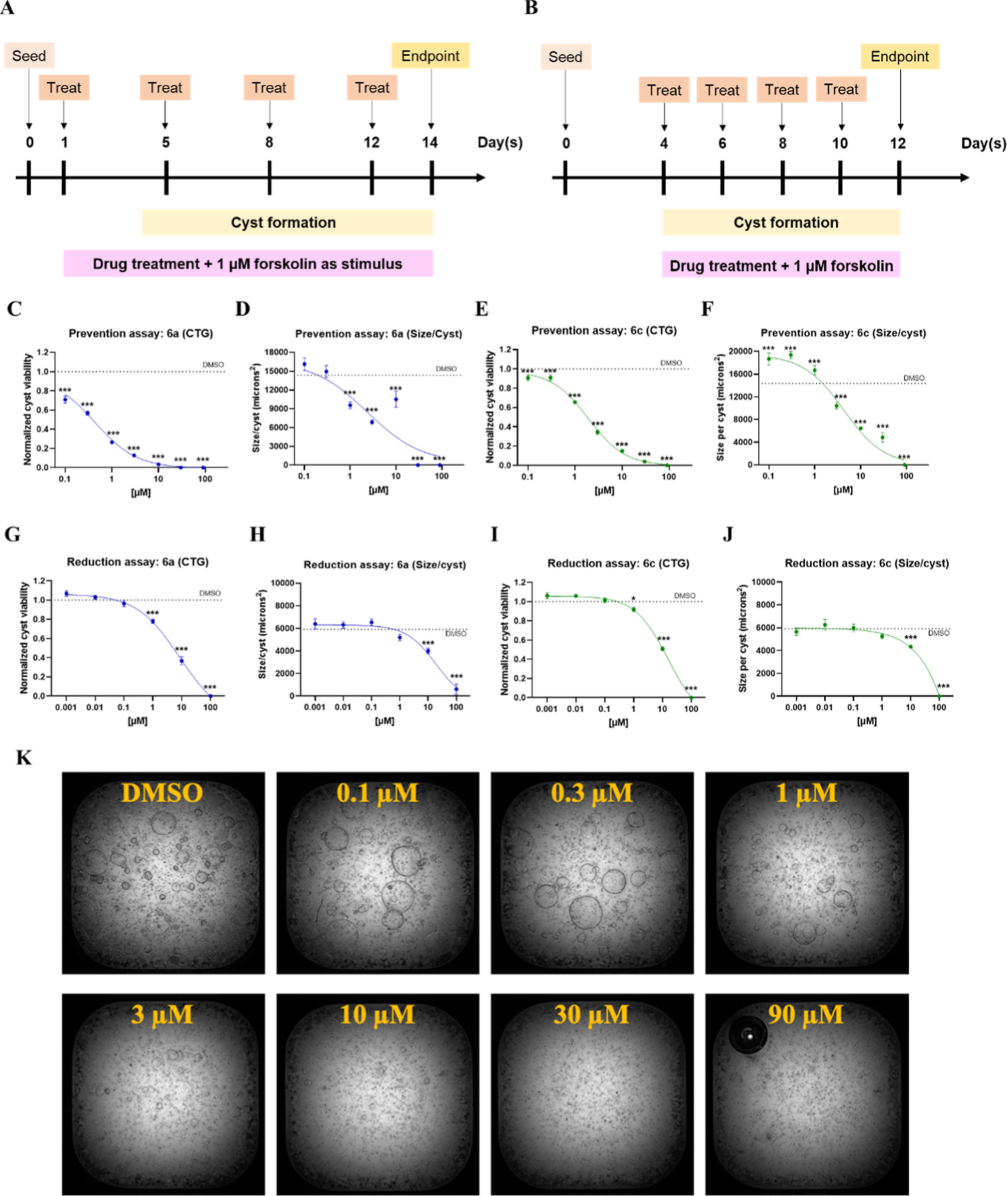
Timeline of (A) prevention and (B) reduction assay for human ADPKD cyst model. (C–F) Prevention assay and (G–J) Reduction assay assessing cyst cell viability (measured by CTG) and average cyst size (size/cyst) following treatment with compounds **6a** or **6c**, presented as concentration–response curves. (K) Representative images of preventive assay treated with DMSO or **6a** for 14 days. All data are presented as mean ± S.E.M.; DMSO vs treated groups; *p < 0.05; ***p < 0.001; *n* = 10; one-way ANOVA followed by Dunnet’s multiple comparisons test.

**Table 2:** Treatment with **6a**, **6c**, and ACY-1215 in the Primary Human ADPKD Cell against Cyst Metrics^a^

	prevention (pretreatment) assay (IC_50_, μM)				reduction (rescue) assay (IC_50_, μM)			
Compd	CTG^b^	CN^c^	TA^d^	size/cyst^e^	CTG^b^	CN^c^	TA^d^	size/cyst^e^
**6a**	0.42	2.22	0.92	2.38	8.36	13.3	10.8	18.5
**6c**	1.92	13.2	3.96	4.28	15.8	10.5	11.3	15.8

a Data from DBM. Compounds were tested at 6–7 different concentrations for 10 repeats.

b CTG, cell viability measured using CellTiterGlo.

c CN, cyst number.

d TA, total cyst area.

e Size/Cyst, average size per cyst.

Next, compounds **6a** and **6c** were compared with ACY-1215, using the same *in vitro* ADPKD model (Figure 6). The results demonstrated that compounds **6a**, **6c**, and ACY-1215 effectively inhibited cyst growth and reduced the cystogenic profile of human ADPKD cells at 10 μM. In addition, compound **6a** significantly outperformed ACY-1215 in reducing cyst viability, cyst number, and total cyst area under reductive treatment. Collectively, these findings suggest that compound **6a** may exert a protective effect against primary human ADPKD 3D cysts, supporting its potential for the prevention and/or treatment of ADPKD.

**Figure 6:**
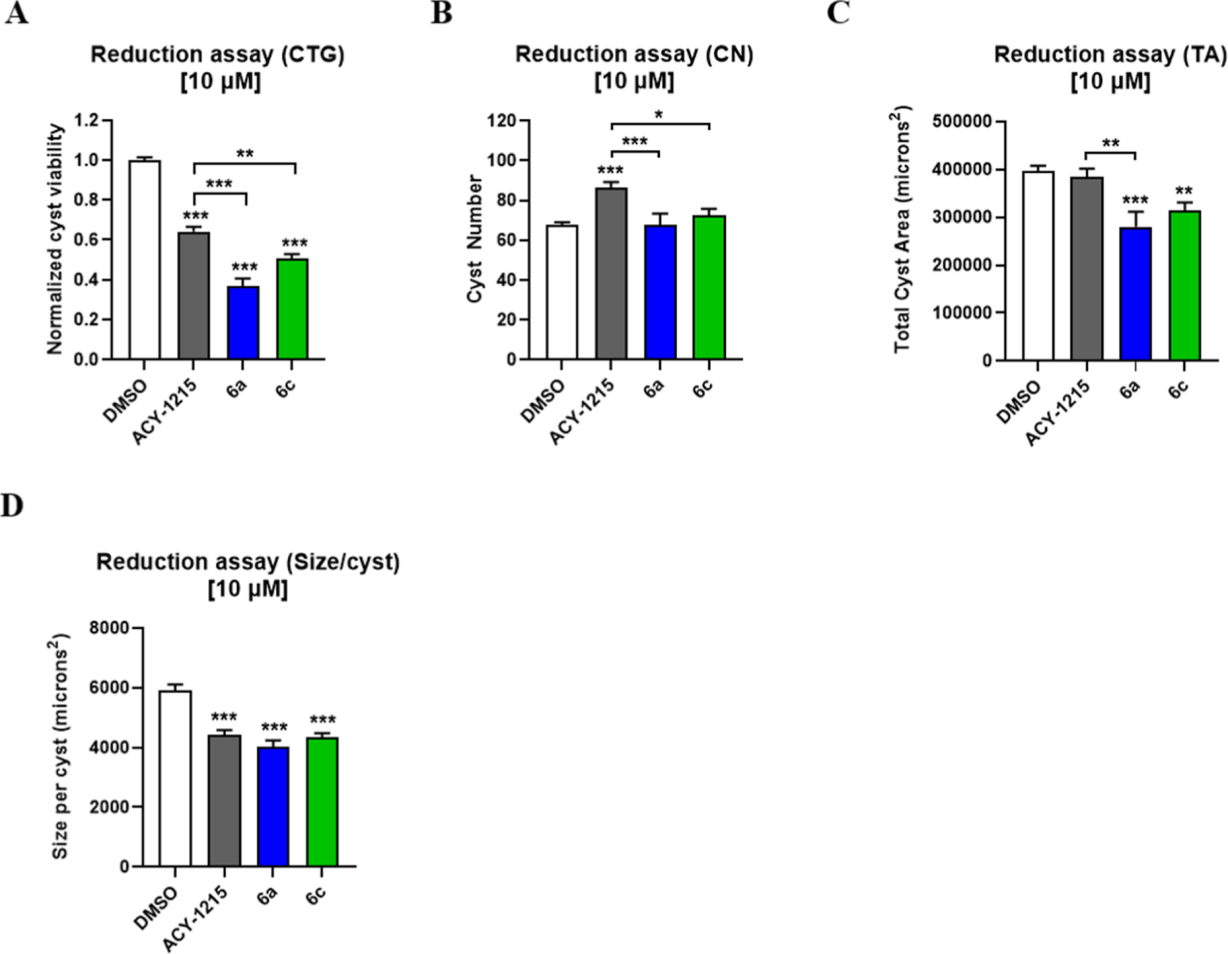
Effects of 10 μM compound **6a** and **6c** compared with ACY-1215 on a human ADPKD cyst model. (A) Cyst cell viability (measured via CTG), (B) cyst number (CN), (C) total cyst area (TA), and (D) average cyst size (size/cyst) were evaluated. All data are presented as mean ± S.E.M.; DMSO vs treated groups; *p < 0.05; **p < 0.01; ***p < 0.001; *n* = 10; one-way ANOVA followed by Turkey’s multiple comparisons test.

### *In Vitro* and *In Vivo* Pharmacokinetic Studies

Given the promising effects of compound **6a** in cell-based studies, *in vitro* PK properties were evaluated. Initially, we examined the thermodynamic solubility of compound **6a** under various conditions (Table 3). Compound **6a** exhibited higher solubility under acidic conditions (pH 2.0: 168.2 μg/mL), which was further enhanced upon dissolving in 0.5% methylcellulose (215.1 μg/mL). This thermodynamic solubility was practically high in the context of drug discovery, particularly prior to formulation development.^41^ Compound **6a** was subsequently assessed for its stability in liver microsomes, hepatocytes, and plasma across multiple species. As shown in Table 3, **6a** demonstrated high stability in liver microsomes (≥88%) and plasma (≥91%) from rat, mouse, and human. *In vitro* metabolic stability assays further revealed moderate intrinsic clearance in rat and mouse hepatocytes, with corresponding half-lives of 26.7 and 59.4 min, respectively. In contrast, incubation with human hepatocytes indicated a slower metabolic turnover, with a half-life of 259 min. Plasma protein binding analysis showed that **6a** exhibited moderate binding in rat and mouse plasma (51.9–56.1%), whereas a higher binding affinity was observed in human plasma (77.5–79.8%).

**Table 3:** Solubility and ADME Properties of Compound **6a**

thermodynamic solubility of compound **6a** (μg/mL)^a^			
pH 2.0	pH 6.8	H_2_O	0.5% methylcellulose
168.2	45.1	50.7	215.1

In vitro ADME parameters of compound **6a**				
exposure	parameter (unit)	rat	mouse	human
liver microsome^b^	% remaining at 60 min	88.3	90.3	96.6
hepatocyte^c^	*t*_1/2_ (min)	26.7	59.4	259
	CL_int(hep)_ (μL/min/10^6^cells)	26.0	11.7	2.67
	CL_int(liver)_ (mL/min/kg)	122	139	7.43
plasma	% remaining at 120 min^d^	91.0	93.9	95.1
	*t*_1/2_ (min)^d^	>289	>289	>289
	PPB (%)^e^	51.9–56.1	53.0–55.0	77.5–79.8

a Solubility was tested at rt (25 °C) once.

b Compounds were tested at 10 μM for 1 h.

c Compounds were tested at 1.0 μM for up to 120 min.

d Compounds were tested at 2.0 μM for up to 120 min.

e Compounds were tested at 0.1–10 μM in triplicate for 4 h.

In rat PK studies, compound **6a** was administered intravenously at 1 mg/kg and orally at 30 mg/kg (Table 4). The results showed that **6a** exhibited an apparently shorter half-life following intravenous administration (0.173 ± 0.008 h) compared to oral dosing (2.94 ± 1.36 h). The area under the plasma concentration–time curve (AUC_(0–∞)_) was 1087 ± 210 ng·h/mL for the oral route and 99.8 ± 15.2 ng·h/mL for IV administration. The oral bioavailability of **6a** was 36%, which is considered acceptable. In addition, plasma and kidney exposures were also determined following oral administration (Table 5). The relatively rapid plasma clearance (*t*_1/2_ = 3.90 h), along with the higher kidney AUC_(0–*t*)_ value (1790 ng·h/mL), indicated that compound **6a** preferentially accumulated and sustained in the kidney.

**Table 4:** Rat and Mouse PK Parameters^a^

	rat		mouse		
parameters (unit)	IV	PO	IV	PO	PO
dose (mg/kg)	1	30	1	30	60
*C*_max_ (ng/mL)	C_0_: 559 ± 62	480 ± 69	C_0_: 1222 ± 204	635 ± 168	834 ± 85
*t*_1/2_ (h)	0.173 ± 0.008	2.94 ± 1.36	0.200 ± 0.109	2.49 ± 1.37	5.70 ± 4.72
*t*_max_ (h)	-	0.250 ± 0.000	-	0.250 ± 0.000	0.250 ± 0.000
AUC_(0‑t)_ (ng·h/mL)	98.5 ± 15.0	1018 ± 271	184 ± 20	643 ± 65	1408 ± 130
AUC_(0‑∞)_ (ng·h/mL)	99.8 ± 15.2	1087 ± 210	184 ± 20	694 ± 59	2561 ± 1544
bioavailability, *F* (%)	-	36	-	13	13

a PK measured in male Sprague–Dawley rats and male CD-1 (ICR) mice, *n* = 3/group. Data are presented as mean ± SD.

**Table 5:** Rat Plasma and Tissue PK Parameters^a^

	*C*_max_ (ng/mL)	*t*_1/2_ (h)	AUC_(0–*t*)_ (ng·h/mL)	AUC_(0–∞)_ (ng·h/mL)
plasma	264	3.90	989	997
kidney	226	5.98	1790	1880

a PK measured in male Sprague–Dawley rats, 30 mg/kg PO, *n* = 3/time point, data were collected at six different time points.

For mouse PK studies, compound **6a** was given intravenously at 1 mg/kg and orally at 30 or 60 mg/kg (Table 4). Consistent with the results of PK profiles obtained in rats, compound **6a** exhibited a relatively long half-life following oral administration. Although the oral bioavailability of mice (13%) was slightly lower than that in rats (36%), an increase in systemic exposure was noted at the higher dose (60 mg/kg) without evidence of saturation, suggesting a dose-dependent trend. These encouraging PK data provide a strong basis for advancing *in vivo* investigations of therapeutic efficacy.

### Oral Administration of Compound **6a** Decreased Kidney Weight and Cyst Area in ADPKD Mouse Model

Bilateral renal cyst formation is a defining characteristic of ADPKD. Marine PKD models have been extensively characterized, with many exhibiting phenotypes closely resemble human PKD, including cyst morphology, cyst localization, and disease progression.^42–44^ While homozygous *Pkd1* knockout mice exhibit embryonic lethality, transgenic knockdown mice with a 60–70% reduction in *Pkd1* expression display relatively slower cyst progression, resembling the gradual development of chronic kidney disease observed in human ADPKD.^45^ In this reported model, cyst expansion and interstitial fibrosis progressed between postnatal days 14 and 60.^45–47^ This enables us to utilize a reliable platform for therapeutic evaluation that closely reflects the natural progression of ADPKD.

In this study, the aforementioned *Pkd1* knockdown mouse model was employed to assess the therapeutic efficacy of **6a** (GV-001, Figure 7). Mice were administered daily oral doses of GV-001 (0, 60, or 120 mg/kg) from postnatal day 15 to 28 in a randomized and blinded design. Dose selection was guided by a separate PK study using the same formulation in mice (Table S1). *In vitro* plasma protein binding in mouse plasma was 53.0–55.0% (Table 3), indicating a substantial unbound fraction of GV-001. Based on the overall PK profile and *in vitro* potency for cyst inhibition, doses of 60 and 120 mg/kg were selected to ensure adequate systemic exposure. Following treatment, the mice were sacrificed and both kidneys were collected for analysis. Treatment with 60 and 120 mg/kg GV-001 (3.4 ± 0.5% and 3.2 ± 0.4%) led to a significant reduction in the average kidney-to-body weight ratio (KW/BW) compared to the vehicle group (5.5 ± 0.8%, Figure 7b). In addition, the cystic index was significantly decreased from 34.3 ± 3.4% in the vehicle-treated controls to 22.7 ± 3.3% (60 mg/kg) and 20.7 ± 2.9% (120 mg/kg) in the GV-001-treated groups (Figure 7c), suggesting effective suppression of disease progression. Representative hematoxylin and eosin (H&E)-stained images of kidney tissues are shown in the Figure 7d. Notably, there were no significant differences in body weight between the treatment and vehicle groups throughout the study period (Figure 7a). Altogether, the results indicate that **6a** attenuates the progression of cyst growth in the ADPKD mouse model, thereby supporting its potential as an oral therapeutic treatment.

**Figure 7:**
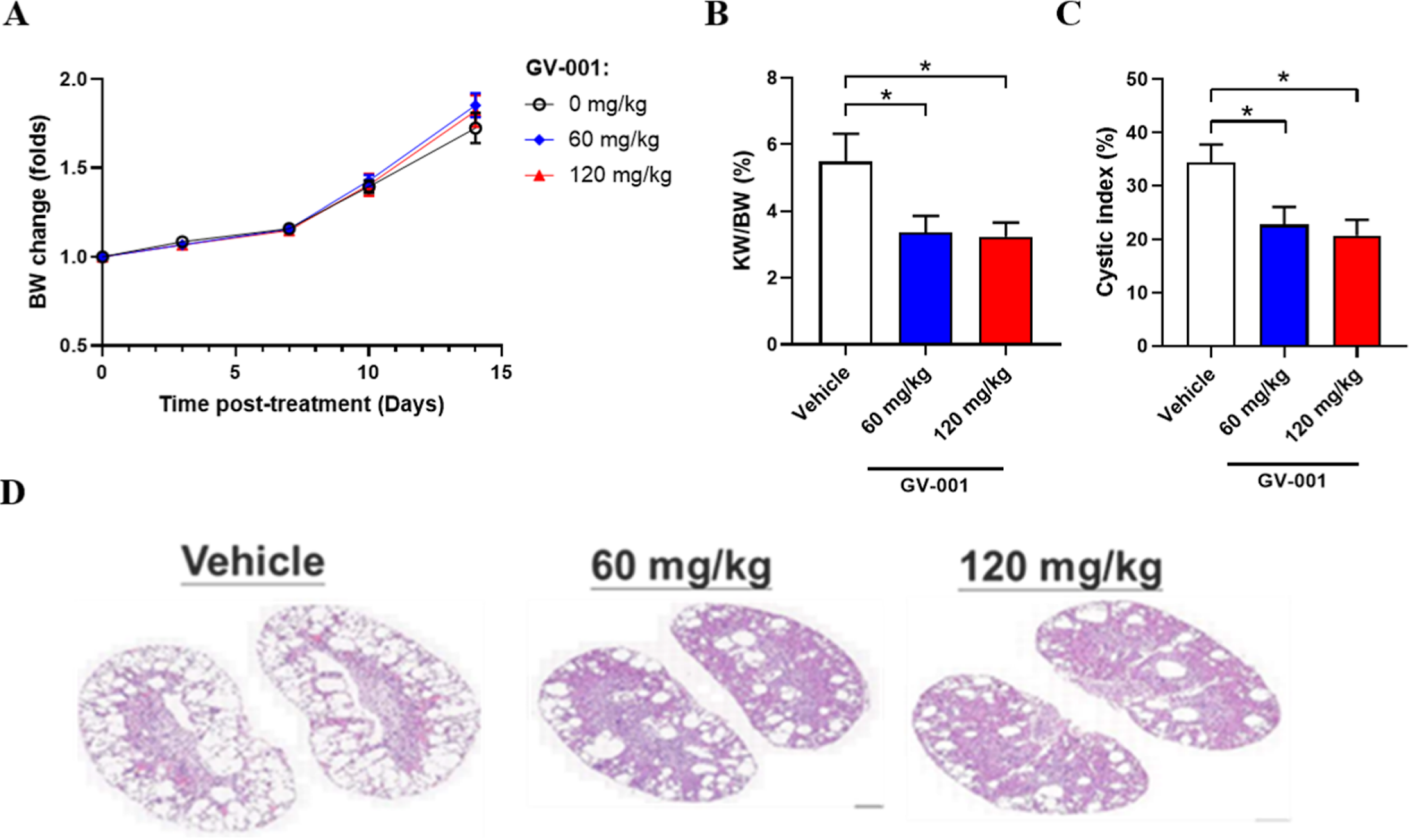
Effects of oral GV-001 treatment on *Pkd1* miRNA transgenic mice. (A) *Pkd1* miRNA transgenic mice were weighed before administration of **6a** twice per week for 14 days. (B) GV-001 treatment inhibited the kidney-to-body weight ratio (KW/BW) in *Pkd1* miRNA transgenic mice. KW/BW (%) = (total kidney weight/BW) × 100%. (C) GV-001 treatment inhibited the cystic index in *Pkd1* miRNA transgenic mice. Cystic index (%) = [Cyst area] Avg./[Kidney area] Avg. × 100%. (D) Representative images of H&E staining of kidney tissues from each group. All data are presented as mean ± S.E.M.; *p < 0.05; *n* = 14–15; one-way ANOVA followed by Dunnett’s multiple comparison test.

### PC1 Expression Was Increased after Oral Administration of **6a**

A reduction in *PKD1* or *PKD2* gene product levels below a critical point can trigger cyst formation in renal epithelial cells.^42^ Therefore, expression of PC1 and PC2 plays an important role in regulating disease progression. Following the experiment described above, PC1 expression in kidneys was analyzed by immunohistochemical (IHC) staining using a polycystin 1 polyclonal antibody, and examined microscopically by the veterinary pathologist in LASCO. Representative IHC images of the kidney tissues were shown in Figure 8. The results indicated that animals treated with 60 and 120 mg/kg of GV-001 exhibited a significant increase in PC1 expression compared to the control group. This upregulation may help explain the efficacy of orally administered GV-001 in the ADPKD mouse model.

**Figure 8:**
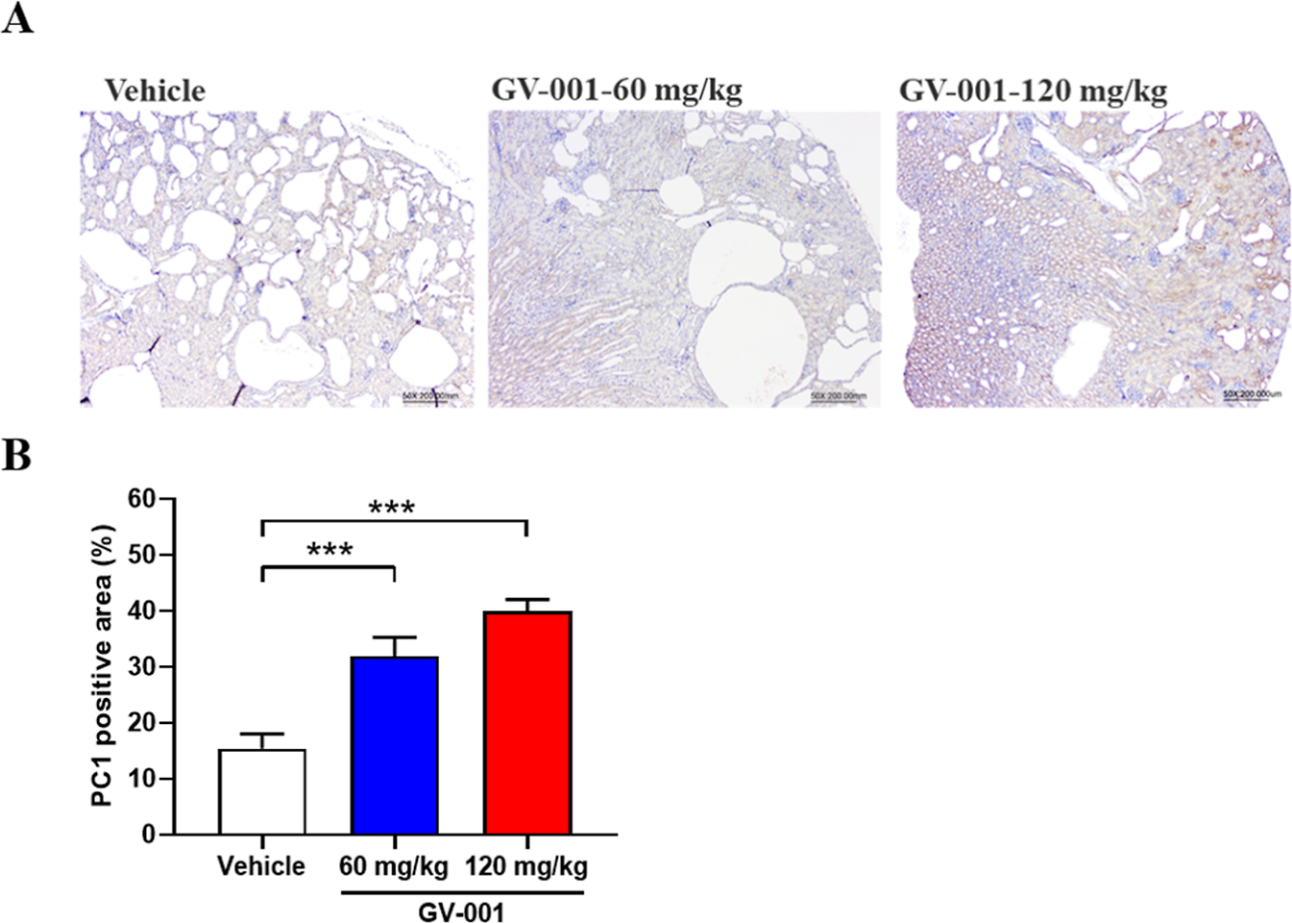
(A) Representative images of PC1 positive area in the ADPKD mouse kidneys, bar = 200 μm; (B) Percentage of PC1 positive area treated with vehicle, 60 mg/kg or 120 mg/kg GV-001 (PO). All data are presented as mean ± S.E.M.; vehicle vs GV-001 treatment group; ***p < 0.001; *n* = 14; one-way ANOVA followed by Dunnett’s multiple comparison test.

## Conclusions

In the field of ADPKD, numerous studies have demonstrated therapeutic effects in both cell-based and mouse models. However, as of the time of this work, no small-molecule drugs have advanced through clinical trials since the approval of tolvaptan in 2009, highlighting a significant unmet need and opportunity for drug discovery in ADPKD. In this study, a series of quinazolin-4-one derivatives were synthesized and evaluated for their HDAC activities. Among them, compound **6a** (GV-001) exhibited potent binding of HDAC6 (IC_50_ = 1.18 nM) with marked selectivity over HDAC1 (1991-fold) and HDAC8 (247-fold). Further investigation demonstrated that GV-001 selectively enhanced the acetylation of α-tubulin in a dose-dependent manner.

While GV-001 was identified as a selective HDAC6 inhibitor, its phenotypic profile was further characterized using the BioMAP Fibrosis Panel. The results indicated that GV-001 modulated key fibrosis-associated biomarkers (decreased collagen I, II; increased collagen IV), and reduced immune-related chemokines (decreased I-TAC and MCP-1). These findings suggest that HDAC6 inhibition by GV-001 exerts both antifibrotic and anti-inflammatory effects. In subsequent *in vitro* studies, cyst proliferation was suppressed without cytotoxicity following treatment with GV-001. Moreover, GV-001 demonstrated excellent *in vitro* metabolic stability and favorable oral PK profiles, supporting its application in *in vivo* efficacy studies. In the transgenic ADPKD mouse model, oral treatment of GV-001 led to a significant reduction in cystic index and increased PC1 expression, underscoring its therapeutic potential for ADPKD. In summary, GV-001 is a selective HDAC6 inhibitor that is orally active. Its antifibrotic and anti-inflammatory properties may also help slow down disease progression, supporting its potential as a novel therapeutic candidate for ADPKD.

## Experimental Sections

### Chemistry

All solvents used were purchased from Merck, JT Baker, Echo chemical, or Thermo Scientific; they were ACS grade and were used without further purification. Anhydrous solvents were prepared using an SP-1 stand-alone solvent purification system (LC Technology Solution) and contained <100 ppm water as determined by Karl Fischer moisture analysis. Chemicals were purchased from Acros, AK Scientific, Aldrich, Alfa Aesar, Carbosynth, Combi-Blocks, Fluka, KM3, Matrix, Thermo Scientific, and TCI and were used as supplied. Reaction progress was monitored by TLC on Merck Kieselgel 60 F_254_ plates. Microwave reactions were carried out using the CEM Discover platform. Flash column chromatography was carried out on Merck silica gel 40 (40–63 μm). ^1^H and ^13^C NMR spectra were obtained on a Bruker, AVIII-600 operating at 600 MHz. Chemical shifts are reported in ppm relative to dimethyl sulfoxide-*d*_6_ (DMSO-*d*_6_) and *d*-chloroform (CDCl_3_), with the central peaks assigned at 2.49 and 7.26 ppm in ^1^H NMR, and at 39.52 and 77.16 in ^13^C NMR. Multiplicity of peaks in ^1^H NMR are defined by the abbreviations s (singlet), d (doublet), t (triplet), q (quartet), m (multiplet), br (broad), doublet of doublet (dd), doublet of triplet (dt), etc. Melting points were determined using a MEL-TEMP instrument (Laboratory Devices, Inc.) and were uncorrected. Low-resolution mass spectrometry (MS) was performed on a Bruker Esquire 2000 using electrospray ionization (ESI) and was analyzed by an ion trap detector. High-resolution MS was performed on a Thermo Scientific Orbitrap QE Plus using ESI and an quadrupole-orbitrap detector. Purity was measured by HPLC, which was carried out on a Shimadzu HPLC system with an SPD-M20A UV–vis Detector and a Kinetex XB-C18 column (5 μm pore size; column dimensions, 100 × 4.60 mm) with a flow rate of 1.5 mL/min and a column temperature of 40 °C. The purity of all biologically tested compounds was ≥95%, and compounds were eluted by acetonitrile and water unless otherwise stated (0.1% formic acid in acetonitrile/0.1% formic acid in H_2_O = 10/90 to 100/0 in 12 min).

### General Procedure A for the Synthesis of Compounds **4a–4l**

To a solution of anthranilic acid (1.0 equiv) in pyridine (1 M) was added 4-bromophenyl acetic acid (1.0 equiv), P­(OPh)_3_ (1.2 equiv) and irradiated by microwave (closed vessel, 250 W) with refluxing for 15 min. Then, the solution was cooled down to rt and added the corresponding amine (1.0–1.4 equiv). The mixture was refluxed under microwave irradiation (closed vessel, 250 W) for another 10 min. Upon completion, the mixture was diluted with EtOAc (50 mL) and extracted with HCl_(aq.)_ (3%, 50 mL) for four times. The combined organic layers were dried over MgSO_4_, filtered, and concentrated *in vacuo*. The crude product was purified by flash column chromatography (silica gel, EtOAc/*n*-heptane = 1/4 to 1/2) and the desired fractions were collected to afford title compound as product.

#### 2-(4-Bromobenzyl)-3-methylquinazolin-4­(3*H*)-one (**4a**)

Following **General Procedure A**, anthranilic acid (10.000 g, 72.9 mmol), 4-bromophenylacetic acid (15.680 g, 72.9 mmol), P­(OPh)_3_ (23.0 mL, 87.5 mmol), and methylammonium chloride (4.920 g, 72.9 mmol) was reacted in pyridine (70 mL) to afford compound **4a** as a yellow solid (4.902 g, 20%). R_*f*_ = 0.33 (EtOAc/*n*-heptane = 1/1); mp 172–174 °C; ^1^H NMR (600 MHz, DMSO-*d*_6_): δ 8.10 (dd, *J* = 7.8, 1.2 Hz, 1H), 7.77 (m, 1H), 7.58 (d, *J* = 7.8 Hz, 1H), 7.52 (d, *J* = 8.4 Hz, 2H), 7.49 (m, 1H), 7.25 (d, *J* = 8.4 Hz, 2H), 4.26 (s, 2H), 3.45 (s, 3H); ^13^C NMR (150 MHz, DMSO-*d*_6_): δ 161.5, 155.9, 146.7, 135.2, 134.2, 131.5, 131.0, 126.8, 126.5, 126.1, 119.9, 119.8, 40.5, 30.4; ESIMS­(+) *m*/*z*: 329, 331 [M + H]^+^.

#### 2-(4-Bromobenzyl)-3-ethylquinazolin-4­(3*H*)-one (**4b**)

Following **General Procedure A**, anthranilic acid (1.508 g, 11.0 mmol), 4-bromophenylacetic acid (2.363 g, 11.0 mmol), P­(OPh)_3_ (3.5 mL, 13.3 mmol), and ethylamine hydrochloride (1.070 g, 13.1 mmol) was reacted in pyridine (11 mL) to afford compound **4b** as a yellow solid (1.318 g, 35%). R_*f*_ = 0.42 (EtOAc/*n*-heptane = 1/1); mp 155–156 °C; ^1^H NMR (600 MHz, DMSO-*d*_6_): δ 8.11 (dd, *J* = 8.1, 0.8 Hz, 1H), 7.81–7.78 (m, 1H), 7.61 (d, *J* = 8.1 Hz, 1H), 7.54–7.49 (m, 3H), 7.30 (d, *J* = 8.3 Hz, 2H), 4.28 (s, 2H), 4.02 (q, *J* = 7.0 Hz, 2H), 1.09 (t, *J* = 7.1 Hz, 3H); ^13^C NMR (150 MHz, DMSO-*d*_6_): δ 161.1, 155.4, 146.8, 135.6, 134.3, 131.5, 131.0, 126.9, 126.7, 126.1, 120.1, 120.0, 40.2, 13.5; ESIMS­(+) *m*/*z*: 343, 345 [M + H]^+^.

#### 2-(4-Bromobenzyl)-3-cyclopropylquinazolin-4­(3*H*)-one (**4c**)

Following **General Procedure A**, anthranilic acid (10.000 g, 72.9 mmol), 4-bromophenylacetic acid (15.680 g, 72.9 mmol), P­(OPh)_3_ (23.0 mL, 87.5 mmol), and cyclopropylamine (5.1 mL, 72.9 mmol) was reacted in pyridine (70 mL) to afford compound **4c** as a yellow solid (2.740 g, 37%). R_*f*_ = 0.37 (EtOAc/*n*-heptane = 1/1); mp 132–134 °C; ^1^H NMR (600 MHz, DMSO-*d*_6_): δ 8.05 (dd, *J* = 7.8, 1.2 Hz, 1H), 7.73 (m, 1H), 7.52 (d, *J* = 8.4 Hz, 1H), 7.50 (d, *J* = 8.4 Hz, 2H), 7.45 (m, 1H), 7.28 (d, *J* = 8.4 Hz, 2H), 4.37 (s, 2H), 2.71 (m, 1H), 1.18 (m, 2H), 0.90 (m, 2H); ^13^C NMR (150 MHz, DMSO-*d*_6_): δ 162.2, 157.8, 146.5, 135.9, 134.0, 131.4, 131.3, 126.6, 126.4, 126.0, 120.7, 119.8, 40.3, 27.1, 10.2; ESIMS­(+) *m*/*z*: 355, 357 [M + H]^+^.

#### 2-(4-Bromobenzyl)-3-(cyclopropylmethyl)­quinazolin-4­(3*H*)-one (**4d**)

Following **General Procedure A**, anthranilic acid (2.001 g, 14.6 mmol), 4-bromophenylacetic acid (3.134 g, 14.6 mmol), P­(OPh)_3_ (4.6 mL, 17.5 mmol), and cyclopropanemethanamine (1.140 g, 16.0 mmol) was reacted in pyridine (15 mL) to afford compound **4d** as a white solid (2.523 g, 47%). R_*f*_ = 0.53 (EtOAc/*n*-heptane = 1/1); mp 89–90 °C; ^1^H NMR (600 MHz, CDCl_3_): δ 8.26 (dd, *J* = 8.0, 1.3 Hz, 1H), 7.75–7.72 (m, 1H), 7.68 (d, *J* = 8.0 Hz, 1H), 7.48–7.45 (m, 1H), 7.45–7.42 (m, 2H), 7.14 (d, *J* = 8.3 Hz, 2H), 4.25 (s, 2H), 3.93 (d, *J* = 6.8 Hz, 2H), 1.12–1.05 (m, 1H), 0.55–0.52 (m, 2H), 0.47–0.44 (m, 2H); ^13^C NMR (150 MHz, CDCl_3_): δ 162.7, 154.7, 147.2, 134.5, 134.4, 132.2, 130.1, 127.1, 127.0, 126.9, 121.4, 120.9, 47.6, 41.7, 11.0, 4.4; ESIMS­(+) *m*/*z*: 369, 371 [M + H]^+^.

#### 2-(4-Bromobenzyl)-3-(phenyl)­quinazolin-4­(3*H*)-one (**4e**)

Following **General Procedure A**, anthranilic acid (0.996 g, 7.3 mmol), 4-bromophenylacetic acid (1.564 g, 7.3 mmol), P­(OPh)_3_ (2.3 mL, 8.8 mmol), and aniline (0.821 g, 8.8 mmol) was reacted in pyridine (8.0 mL) to afford compound **4e** as a white solid (1.977 g, 70%). R_*f*_ = 0.45 (EtOAc/*n*-heptane = 1/1); mp 134–136 °C; ^1^H NMR (600 MHz, CDCl_3_): δ 8.28–8.27 (m, 1H), 7.82–7.80 (m, 1H), 7.78 (d, *J* = 7.5 Hz, 1H), 7.52–7.49 (m, 1H), 7.48–7.45 (m, 1H), 7.44–7.41 (m, 2H), 7.28 (dt, *J* = 8.3, 2.3 Hz, 2H), 6.97 (d, *J* = 7.3 Hz, 2H), 6.74 (d, *J* = 8.3 Hz, 2H), 3.85 (s, 2H); ^13^C NMR (150 MHz, CDCl_3_): δ 162.5, 154.9, 147.2, 136.8, 134.9, 134.3, 131.6, 130.5, 129.7, 129.5, 128.8, 127.3, 127.3, 121.1, 121.0, 42.1; ESIMS­(+) *m*/*z*: 391, 393 [M + H]^+^.

#### 2-(4-Bromobenzyl)-3-(2-chlorophenyl)­quinazolin-4­(3*H*)-one (**4f**)

Following **General Procedure A**, anthranilic acid (2.000 g, 14.6 mmol), 4-bromophenylacetic acid (3.200 g, 14.9 mmol), P­(OPh)_3_ (4.5 mL, 17.2 mmol), and 2-chloroaniline (1.9 mL, 18.1 mmol) was reacted in pyridine (9 mL) to afford compound **4f** as a yellow solid (4.350 g, 80%). R_*f*_ = 0.44 (EtOAc/*n*-heptane = 1/1); mp 152–154 °C; ^1^H NMR (600 MHz, DMSO-*d*_6_): δ 8.13 (dd, *J* = 7.8, 1.2 Hz, 1H), 7.87 (m, 1H), 7.71 (d, *J* = 8.4 Hz, 1H), 6.61 (dd, *J* = 8.4, 1.2 Hz, 1H), 7.56–7.53 (m, 2H), 7.50–7.36 (m, 2H), 7.37 (d, *J* = 8.4 Hz, 2H), 6.85 (d, *J* = 8.4 Hz, 2H), 3.36 (s, 2H); ^13^C NMR (150 MHz, DMSO-*d*_6_): δ 160.5, 154.4, 146.9, 134.9, 134.2, 134.1, 131.9, 131.1, 130.96, 130.94, 130.0, 128.3, 127.1, 127.09, 126.4, 120.2, 120.0, 40.8; ESIMS­(+) *m*/*z*: 425, 427 [M + H]^+^.

#### 2-(4-Bromobenzyl)-3-(4-chlorophenyl)­quinazolin-4­(3*H*)-one (**4g**)

Following **General Procedure A**, anthranilic acid (2.000 g, 14.6 mmol), 4-bromophenylacetic acid (3.230 g, 15.0 mmol), P­(OPh)_3_ (4.5 mL, 17.2 mmol), and 4-chloroaniline (1.860 g, 14.6 mmol) was reacted in pyridine (9 mL) to afford compound **4g** as a yellow solid (2.348 g, 43%). R_*f*_ = 0.54 (EtOAc/*n*-heptane = 1/1); mp 138–140 °C; ^1^H NMR (600 MHz, DMSO-*d*_6_): δ 8.10 (d, *J* = 7.2 Hz, 1H), 7.84 (m, 1H), 7.67 (d, *J* = 7.8 Hz, 1H), 7.54 (m, 1H), 7.52 (d, *J* = 7.8 Hz, 2H), 7.39 (d, *J* = 7.8 Hz, 2H), 7.30 (d, *J* = 7.8 Hz, 2H), 6.93 (d, *J* = 7.2 Hz, 2H), 3.79 (s, 2H); ^13^C NMR (150 MHz, DMSO-*d*_6_): δ 161.4, 154.8, 147.0, 135.8, 135.0, 134.7, 133.6, 131.0, 130.9, 130.8, 129.2, 127.0, 126.9, 126.3, 120.5, 119.7, 41.1; ESIMS­(+) *m*/*z*: 425, 427 [M + H]^+^.

#### 2-(4-Bromobenzyl)-3-(2,6-dimethylphenyl)­quinazolin-4­(3*H*)-one (**4h**)

Following **General Procedure A**, anthranilic acid (2.000 g, 14.6 mmol), 4-bromophenylacetic acid (3.400 g, 15.8 mmol), P­(OPh)_3_ (4.5 mL, 17.2 mmol), and 2,6-dimethylaniline (2.2 mL, 17.9 mmol) was reacted in pyridine (9 mL) to afford compound **4h** as a white solid (3.673 g, 60%). R_*f*_ = 0.49 (EtOAc/*n*-heptane = 1/1); mp 100–104 °C; ^1^H NMR (600 MHz, DMSO-*d*_6_): δ 8.14 (dd, *J* = 7.8, 1.2 Hz, 1H), 7.88 (m, 1H), 7.75 (d, *J* = 7.8 Hz, 1H), 7.56 (m, 1H), 7.39 (d, *J* = 8.4 Hz, 2H), 7.34 (m, 1H), 7.21 (d, *J* = 7.8 Hz, 2H), 6.82 (d, *J* = 8.4 Hz, 2H), 3.64 (s, 2H), 1.73 (s, 6H); ^13^C NMR (150 MHz, DMSO-*d*_6_): δ 160.5, 154.9, 147.2, 135.5, 135.1, 135.0, 134.1, 131.3, 131.1, 129.4, 128.9, 127.32, 127.30, 126.6, 120.3, 120.2, 115.3, 40.7, 17.1; ESIMS­(+) *m*/*z*: 419, 421­[M + H]^+^.

#### 2-(4-Bromobenzyl)-3-(4-methoxyphenyl)­quinazolin-4­(3*H*)-one (**4i**)

Following **General Procedure A**, anthranilic acid (2.000 g, 14.6 mmol), 4-bromophenylacetic acid (3.210 g, 14.9 mmol), P­(OPh)_3_ (4.5 mL, 17.2 mmol), and 2,6-dimethylaniline (2.040 g, 16.6 mmol) was reacted in pyridine (9 mL) to afford compound **4i** as a yellow solid (3.800 g, 62%). R_*f*_ = 0.43 (EtOAc/*n*-heptane = 1/1); mp 95–97 °C; ^1^H NMR (600 MHz, DMSO-*d*_6_): δ 8.10 (dd, *J* = 7.8, 0.6 Hz, 1H), 7.82 (m, 1H), 7.65 (d, *J* = 8.4 Hz, 1H), 7.52 (m, 1H), 7.38 (d, *J* = 8.4 Hz, 2H), 7.14 (d, *J* = 9.0 Hz, 2H), 6.98 (d, *J* = 8.4 Hz, 2H), 6.92 (d, *J* = 8.4 Hz, 2H), 3.80 (s, 3H), 3.79 (s, 2H); ^13^C NMR (150 MHz, DMSO-*d*_6_): δ 161.7, 159.3, 155.7, 147.0, 135.2, 134.6, 131.0, 129.9, 129.4, 127.0, 126.8, 126.3, 120.7, 120.6, 119.7, 114.4, 55.4, 41.1; ESIMS­(+) *m*/*z*: 421, 423 [M + H]^+^.

#### 2-(4-Bromobenzyl)-6-chloro-3-methylquinazolin-4­(3*H*)-one (**4j**)

Following **General Procedure A**, 2-amino-5-chlorobenzoic acid (1.010 g, 5.9 mmol), 4-bromophenylacetic acid (1.310 g, 6.1 mmol), P­(OPh)_3_ (1.8 mL, 6.9 mmol), and methylammonium chloride (0.466 g, 6.9 mmol) was reacted in pyridine (6 mL) to afford compound **4j** as a yellow solid (1.443 g, 78%). R_*f*_ = 0.49 (EtOAc/*n*-heptane = 1/1); mp 139–142 °C; ^1^H NMR (600 MHz, DMSO-*d*_6_): δ 8.03 (d, *J* = 9.0 Hz, 1H), 7.79 (dd, *J* = 9.0, 3.0 Hz, 1H), 7.60 (d, *J* = 3.0 Hz, 1H), 7.52 (d, *J* = 8.4 Hz, 2H), 7.25 (d, *J* = 8.4 Hz, 2H), 4.26 (s, 2H), 3.46 (s, 3H); ^13^C NMR (150 MHz, DMSO-*d*_6_): δ 160.5, 156.6, 145.5, 134.9, 134.3, 131.5, 131.1, 130.7, 129.2, 125.1, 121.0, 120.0, 40.5, 30.6; ESIMS­(+) *m*/*z*: 363, 365 [M + H]^+^.

#### 2-(4-Bromobenzyl)-6-chloro-3-cyclopropylquinazolin-4­(3*H*)-one (**4k**)

Following **General Procedure A**, 2-amino-5-chlorobenzoic acid (3.000 g, 17.5 mmol), 4-bromophenylacetic acid (4.000 g, 18.6 mmol), P­(OPh)_3_ (5.4 mL, 20.6 mmol), and cyclopropylamine (1.6 mL, 23.0 mmol) was reacted in pyridine (15 mL) to afford compound **4k** as a yellow solid (2.172 g, 32%). R_*f*_ = 0.56 (EtOAc/*n*-heptane = 1/1); mp 160–162 °C; ^1^H NMR (600 MHz, DMSO-*d*_6_): δ 7.97 (d, *J* = 8.4 Hz, 1H), 7.74 (dd, *J* = 8.4, 2.4 Hz, 1H), 7.53 (d, *J* = 9.0 Hz, 1H), 7.50 (d, *J* = 8.4 Hz, 2H), 7.28 (d, *J* = 8.4 Hz, 2H), 4.37 (s, 2H), 2.74 (m, 1H), 1.18 (m, 2H), 0.91 (m, 2H); ^13^C NMR (150 MHz, DMSO-*d*_6_): δ 161.2, 158.5, 145.2, 135.7, 134.1, 131.4, 131.2, 130.6, 128.9, 124.9, 122.0, 119.8, 40.2, 27.2, 10.1; ESIMS­(+) *m*/*z*: 389, 391 [M + H]^+^.

#### 2-(4-Bromobenzyl)-6-fluoro-3-methylquinazolin-4­(3*H*)-one (**4l**)

Following **General Procedure A**, 2-amino-5-fluorobenzoic acid (2.000 g, 12.9 mmol), 4-bromophenylacetic acid (3.120 g, 14.5 mmol), P­(OPh)_3_ (4.0 mL, 15.3 mmol), and methylammonium chloride (1.210 g, 17.9 mmol) was reacted in pyridine (9 mL) to afford compound **4l** as a yellow solid (2.380 g, 53%). R_*f*_ = 0.38 (EtOAc/*n*-heptane = 1/1); mp 126–130 °C; ^1^H NMR (600 MHz, DMSO-*d*_6_): δ 7.74 (m, 1H), 7.65–7.64 (m, 2H), 7.51 (d, *J* = 8.4 Hz, 2H), 7.25 (d, *J* = 7.8 Hz, 2H), 4.25 (s, 2H), 3.45 (s, 3H); ^13^C NMR (150 MHz, DMSO-*d*_6_): δ 160.9, 159.9 (d, *J* = 243.5 Hz), 155.4, 143.6, 135.0, 131.5, 131.1, 129.7 (d, *J* = 8.3 Hz), 122.7 (d, *J* = 23.9 Hz), 120.9 (d, *J* = 8.4 Hz), 120.0, 110.6 (d, *J* = 23.1 Hz), 40.4, 30.5; ESIMS­(+) *m*/*z*: 347, 349 [M + H]^+^.

#### General Procedure B for the Synthesis of Compounds **5a–5l**

To a solution of bromo-substituted intermediate compound (1.0 equiv) in DMAC and MeOH (1:1, degassed) was added Mo­(CO)_6_ (1.5 equiv), Pd­(OAc)_2_ (8 mol %), Xantphos (16 mol %), DMAP (2.0 equiv), and DIPEA (2.0 equiv) under Ar. The reaction mixture was heated to reflux by hot plate for 16 h or by microwave (opened-vessel, 150 W) for 1 h. After the reaction was completed, the mixture was filtered through a short pad of Celite, and the filtrate was concentrated *in vacuo* to give black liquid. The liquid was diluted with water (20x volume of DMAC), and the resulting precipitate was filtered to afford brown solid. The crude solid was purified by flash column chromatography (silica gel, EtOAc/*n*-heptane = 0/1 to 1/1), and the desired fractions were collected to afford title compound as product.

#### Methyl 4-[(3-Methyl-4-oxo-3,4-dihydroquinazolin-2-yl)­methyl]­benzoate (**5a**)

Following **General Procedure B**, compound **4a** (4.000 g, 12.2 mmol) was reacted to afford compound **5a** as a yellow solid (2.120 g, 57%). R_*f*_ = 0.35 (EtOAc/*n*-heptane = 3/2); mp 142–144 °C; ^1^H NMR (600 MHz, DMSO-*d*_6_): δ 8.11 (dd, *J* = 7.8, 0.6 Hz, 1H), 7.92 (d, *J* = 7.8 Hz, 2H), 7.78 (m, 1H), 7.58 (d, *J* = 7.8 Hz, 1H), 7.49 (m, 1H), 7.43 (d, *J* = 8.4 Hz, 2H), 4.38 (s, 2H), 3.83 (s, 3H), 3.45 (s, 3H); ^13^C NMR (150 MHz, DMSO-*d*_6_): δ 166.0, 161.5, 155.7, 146.8, 141.4, 134.2, 129.5, 129.2, 128.2, 126.8, 126.6, 126.1, 119.8, 52.0, 41.2, 30.4; ESIMS­(+) *m*/*z*: 309 [M + H]^+^.

#### Methyl 4-[(3-Ethyl-4-oxo-3,4-dihydroquinazolin-2-yl)­methyl]­benzoate (**5b**)

Following **General Procedure B**, compound **4b** (0.503 g, 1.5 mmol) was reacted to afford compound **5b** as an orange solid (0.292 g, 62%). R_*f*_ = 0.33 (EtOAc/*n*-heptane = 1/1); mp 142–144 °C; ^1^H NMR (600 MHz, DMSO-*d*_6_): δ 8.12 (d, *J* = 7.8 Hz, 1H), 7.93 (d, *J* = 8.2 Hz, 2H), 7.79 (t, *J* = 7.3 Hz, 1H), 7.60 (d, *J* = 8.1 Hz, 1H), 7.52–7.47 (m, 3H), 4.39 (s, 2H), 4.02 (q, *J* = 7.0 Hz, 2H), 3.83 (s, 3H), 1.07 (t, *J* = 7.0 Hz, 3H); ^13^C NMR (150 MHz, DMSO-*d*_6_): δ 166.0, 161.1, 155.2, 146.8, 141.9, 134.3, 129.5, 129.2, 128.2, 126.9, 126.7, 126.1, 120.1, 52.1, 40.8, 13.5; ESIMS­(+) *m*/*z*: 323 [M + H]^+^.

#### Methyl 4-[(3-Cyclopropyl-4-oxo-3,4-dihydroquinazolin-2-yl)­methyl]­benzoate (**5c**)

Following **General Procedure B**, compound **4c** (2.000 g, 8.4 mmol) was reacted to afford compound **5c** as a white solid (1.920 g, 68%). R_*f*_ = 0.36 (EtOAc/*n*-heptane = 1/1); mp 141–144 °C; ^1^H NMR (600 MHz, DMSO-*d*_6_): δ 8.05 (d, *J* = 7.2 Hz, 1H), 7.91 (d, *J* = 7.8 Hz, 2H), 7.74 (m, 1H), 7.51 (d, *J* = 7.8 Hz, 1H), 7.46 (d, *J* = 7.8 Hz, 2H), 4.49 (s, 2H), 3.83 (s, 3H), 2.70 (m, 1H), 0.92 (m, 2H), 0.90 (m, 2H); ^13^C NMR (150 MHz, DMSO-*d*_6_): δ 166.1, 162.2, 157.7, 146.5, 142.2, 134.1, 129.5, 129.3, 126.6, 126.5, 126.0, 120.8, 52.0, 40.9, 27.1, 10.2; ESIMS­(+) *m*/*z*: 335 [M + H]^+^.

#### Methyl 4-{[3-(Cyclopropylmethyl)-4-oxo-3,4-dihydroquinazolin-2-yl]­methyl]}­enzoate (**5d**)

Following **General Procedure B**, compound **4d** (2.450 g, 6.3 mmol) was reacted to afford compound **5d** as a yellow solid (1.060 g, 49%). R_*f*_ = 0.42 (EtOAc/*n*-heptane = 1/1); mp 112–113 °C; ^1^H NMR (600 MHz, DMSO-*d*_6_): δ 8.12 (dd, *J* = 8.0, 1.3 Hz, 1H), 7.94–7.93 (m, 2H), 7.81–7.78 (m, 1H), 7.58 (d, *J* = 8.1 Hz, 1H), 7.52–7.50 (m, 1H), 7.47 (d, *J* = 8.3 Hz, 2H), 4.43 (s, 2H), 3.96 (d, *J* = 7.0 Hz, 2H), 3.84 (s, 3H), 1.19–1.14 (m, 1H), 0.45–0.41 (m, 2H), 0.39–0.37 (m, 2H); ^13^C NMR (150 MHz, DMSO-*d*_6_): δ 166.1, 161.7, 155.3, 146.8, 141.9, 134.5, 129.5, 129.3, 128.2, 126.9, 126.8, 126.3, 120.2, 52.1, 47.1, 40.7, 10.5, 3.9; ESIMS­(+) *m*/*z*: 349 [M + H]^+^.

#### Methyl 4-[(3-Phenyl-4-oxo-3,4-dihydroquinazolin-2-yl)­methyl]­benzoate (**5e**)

Following **General Procedure B**, compound **4e** (1.827 g, 4.7 mmol) was reacted to afford compound **5e** as an off-white solid (0.921 mg, 53%). R_*f*_ = 0.40 (EtOAc/*n*-heptane = 1/1); mp 179–182 °C; ^1^H NMR (600 MHz, CDCl_3_): δ 8.28 (d, *J* = 7.9 Hz, 1H), 7.84–7.81 (m, 4H), 7.53–7.50 (m, 1H), 7.46–7.44 (t, *J* = 7.5 Hz, 1H), 7.39 (t, *J* = 7.7 Hz, 2H), 6.95–6.93 (m, 4H), 3.97 (s, 2H), 3.89 (s, 3H); ^13^C NMR (150 MHz, DMSO-*d*_6_): δ 166.1, 161.5, 155.0, 147.1, 141.4, 136.9, 134.7, 129.2, 129.1, 129.0, 128.9, 127.9, 127.1, 126.9, 126.4, 120.7, 52.1, 41.9; ESIMS­(+) *m*/*z*: 371 [M + H]^+^.

#### Methyl 4-{[3-(2-Chlorophenyl)-4-oxo-3,4-dihydroquinazolin-2-yl]­methyl}­benzoate (**5f**)

Following **General Procedure B**, compound **4f** (2.350 g, 6.3 mmol) was reacted to afford compound **5f** as a yellow solid (1.460 g, 58%). R_*f*_ = 0.22 (EtOAc/*n*-heptane = 1/1); mp 167–169 °C; ^1^H NMR (600 MHz, DMSO-*d*_6_): δ 8.13 (d, *J* = 7.8 Hz, 1H), 7.89 (m, 1H), 7.77 (d, *J* = 7.8 Hz, 2H), 7.72 (d, *J* = 7.8 Hz, 1H), 7.57 (m, 3H), 7.51 (m, 2H), 7.04 (d, *J* = 7.8 Hz, 2H), 3.88 (dd, *J* = 19.8, 15.6, 2H), 3.82 (s, 3H); ^13^C NMR (150 MHz, DMSO-*d*_6_): δ 166.0, 160.6, 154.3, 147.0, 140.4, 135.1, 134.2, 132.0, 131.2, 131.0, 130.0, 129.1, 129.0, 128.3, 128.1, 127.24, 127.22, 126.4, 120.2, 52.0, 41.6; ESIMS­(+) *m*/*z*: 405 [M + H]^+^.

#### Methyl 4-{[3-(4-Chlorophenyl)-4-oxo-3,4-dihydroquinazolin-2-yl]­methyl}­benzoate (**5g**)

Following **General Procedure B**, compound **4g** (1.970 g, 5.3 mmol) was reacted to afford compound **5g** as a brown solid (1.340 g, 63%). R_*f*_ = 0.37 (EtOAc/*n*-heptane = 1/1); mp 169–171 °C; ^1^H NMR (600 MHz, DMSO-*d*_6_): δ 8.11 (d, *J* = 7.8 Hz, 1H), 7.84 (m, 1H), 7.79 (d, *J* = 7.2 Hz, 2H), 7.67 (d, *J* = 8.4 Hz, 1H), 7.55 (m, 1H), 7.48 (d, *J* = 7.8 Hz, 2H), 7.28 (d, *J* = 8.4 Hz, 2H), 7.10 (d, *J* = 7.8 Hz, 2H), 3.91 (s, 2H), 3.82 (s, 3H); ^13^C NMR (150 MHz, DMSO-*d*_6_): δ 166.1, 161.5, 154.6, 147.0, 141.2, 135.8, 134.8, 133.7, 130.8, 129.2, 128.4, 128.0, 127.1, 127.0, 126.4, 120.6, 52.0, 41.8; ESIMS­(+) *m*/*z*: 405 [M + H]^+^.

#### Methyl 4-{[3-(2,6-Dimethylphenyl)-4-oxo-3,4-dihydroquinazolin-2-yl]­methyl}­benzoate (**5h**)

Following **General Procedure B**, compound **4h** (2.000 g, 4.8 mmol) was reacted to afford compound **5h** as a yellow solid (1.303 g, 68%). R_*f*_ = 0.53 (EtOAc/*n*-heptane = 1/1); mp 120–123 °C; ^1^H NMR (600 MHz, DMSO-*d*_6_): δ 8.14 (dd, *J* = 7.8, 1.2 Hz, 1H), 7.89 (m, 1H), 7.79 (d, *J* = 7.8 Hz, 2H), 7.75 (d, *J* = 7.8 Hz, 1H), 7.56 (m, 1H), 7.35 (m, 1H), 7.20 (d, *J* = 7.2 Hz, 2H), 7.01 (8.4 Hz, 2H), 3.81 (s, 3H), 3.75 (s, 2H), 1.71 (s, 6H); ^13^C NMR (150 MHz, DMSO-*d*_6_): δ 165.9, 160.3, 154.6, 147.1, 140.2, 135.4, 135.0, 134.9, 129.4, 129.3, 129.0, 128.7, 128.3, 127.3, 127.2, 126.5, 120.2, 52.0, 41.2, 17.0; ESIMS­(+) *m*/*z*: 399.0 [M + H]^+^.

#### Methyl 4-{[3-(4-Methoxyphenyl)-4-oxo-3,4-dihydroquinazolin-2-yl]­methyl}­benzoate (**5i**)

Following **General Procedure B**, compound **4i** (2.000 g, 4.8 mmol) was reacted to afford compound **5i** as a white solid (1.255 g, 64%). R_*f*_ = 0.32 (EtOAc/*n*-heptane = 1/1); mp 160–162 °C; ^1^H NMR (600 MHz, DMSO-*d*_6_): δ 8.11 (d, *J* = 7.8 Hz, 1H), 7.83 (m, 1H), 7.79 (d, *J* = 7.8 Hz, 2H), 7.65 (d, *J* = 7.8 Hz, 1H), 7.53 (m, 1H), 7.12 (d, *J* = 8.4 Hz, 2H), 7.09 (d, *J* = 8.4 Hz, 2H), 7.82 (d, *J* = 8.4 Hz, 2H), 3.91 (s, 2H), 3.82 (s, 3H), 3.79 (s, 3H); ^13^C NMR (150 MHz, DMSO-*d*_6_): δ 166.1, 161.7, 159.3, 155.5, 147.0, 141.5, 134.6, 129.9, 129.4, 129.1, 127.9, 127.0, 126.8, 126.4, 120.6, 114.4, 55.4, 52.0, 41.8; ESIMS­(+) *m*/*z*: 401 [M + H]^+^.

#### Methyl 4-[(6-Chloro-3-methyl-4-oxo-3,4-dihydroquinazolin-2-yl)­methyl]­benzoate (**5j**)

Following **General Procedure B**, compound **4j** (1.000 g, 2.8 mmol) was reacted to afford compound **5j** as a white solid (0.460 g, 49%). R_*f*_ = 0.35 (EtOAc/*n*-heptane = 1/1); mp 173–175 °C; ^1^H NMR (600 MHz, DMSO-*d*_6_): δ 8.02 (d, *J* = 2.4 Hz, 1H), 7.92 (d, *J* = 8.4 Hz, 2H), 7.78 (dd, *J* = 9.0, 2.4 Hz, 1H), 7.58 (d, *J* = 9.0 Hz, 1H), 7.43 (d, *J* = 8.4 Hz, 2H), 4.37 (s, 2H), 3.83 (s, 3H), 3.45 (s, 3H); ^13^C NMR (150 MHz, DMSO-*d*_6_): δ 166.0, 160.5, 156.4, 145.5, 141.2, 134.3, 130.7, 129.5, 129.3, 129.1, 128.2, 125.1, 121.0, 52.1, 41.1, 30.6; ESIMS­(+) *m*/*z*: 343 [M + H]^+^.

#### Methyl 4-[(6-Chloro-3-cyclopropyl-4-oxo-3,4-dihydroquinazolin-2-yl)­methyl]­benzoate (**5k**)

Following **General Procedure B**, compound **4k** (2.170 g, 5.6 mmol) was reacted to afford compound **5k** as a white solid (1.255 g, 61%). R_*f*_ = 0.4 (EtOAc/*n*-heptane = 1/1); mp 161–163 °C; ^1^H NMR (600 MHz, DMSO-*d*_6_): δ 7.99 (d, *J* = 2.4 Hz, 1H), 7.91 (d, *J* = 7.8 Hz, 2H), 7.76 (dd, *J* = 9.0, 2.4 Hz, 1H), 7.53 (d, *J* = 9.0 Hz, 1H), 7.46 (d, *J* = 7.8 Hz, 2H), 4.48 (s, 2H), 3.83 (s, 3H), 2.74 (m, 1H), 1.17 (m, 2H), 0.92 (m, 2H); ^13^C NMR (150 MHz, DMSO-*d*_6_): δ 166.1, 161.3, 158.4, 145.2, 142.0, 134.2, 130.6, 129.6, 129.3, 129.0, 128.0, 125.0, 122.0, 52.1, 40.9, 27.3, 10.2; ESIMS­(+) *m*/*z*: 369 [M + H]^+^.

#### Methyl 4-[(6-Fluoro-3-methyl-4-oxo-3,4-dihydroquinazolin-2-yl)­methyl]­benzoate (**5l**)

Following **General Procedure B**, compound **4l** (2.000 g, 5.8 mmol) was reacted to afford compound **5l** as a yellow solid (1.550 g, 82%). R_*f*_ = 0.35 (EtOAc/*n*-heptane = 1/1); mp 155–157 °C; ^1^H NMR (600 MHz, DMSO-*d*_6_): δ 7.92 (d, *J* = 8.4 Hz, 2H), 7.77 (m, 1H), 7.66 (m, 2H), 7.43 (d, *J* = 8.4 Hz, 2H), 4.37 (s, 2H), 3.83 (s, 3H), 3.45 (s, 3H); ^13^C NMR (150 MHz, DMSO-*d*_6_): δ 166.0, 160.9, 159.9 (d, *J* = 243.6 Hz), 155.3, 143.7, 141.3, 129.8 (d, *J* = 8.3 Hz), 129.5, 129.2, 128.2, 122.8 (d, *J* = 23.9 Hz), 121.0 (d, *J* = 8.6 Hz), 110.7 (d, *J* = 23.3 Hz), 52.1, 41.0, 30.6; ESIMS­(+) *m*/*z*: 327 [M + H]^+^.

#### General Procedure C for the Synthesis of Compounds **6a–6l**

To a solution of ester intermediates (1.0 equiv) in NH_2_OH solution (2 N in MeOH, 20 equiv) was added NaOH (2.0 equiv) at 0 °C, and the reaction mixture was stirred at rt for 3 h. After the reaction was completed, the mixture was diluted with water and the precipitate was filtered. The filtrate was acidified with 3% HCl_(aq)_ to adjust pH to 7 and the resulting precipitate was filtered to give crude solid. The solid was purified by flash column chromatography (silica gel, MeOH/DCM = 0/1 to 1/25), and the desired fractions were collected to afford title compound as product.

#### 4-{[3-Methylquinazolin-4­(3*H*)-on-2-yl]­methyl}-*N*-hydroxybenzamide (**6a**)

Following **General Procedure C**, compound **5a** (2.000 g, 6.5 mmol) was reacted to afford compound **6a** as a white solid (1.400 g, 70%). R_*f*_ = 0.16 (MeOH/DCM = 1/25); mp 228–230 °C; ^1^H NMR (600 MHz, DMSO-*d*_6_): δ 11.19 (s, 1H), 9.02 (s, 1H), 8.11 (dd, *J* = 8.4, 1.2 Hz, 1H), 7.79–7.76 (m, 1H), 7.72 (d, *J* = 8.4 Hz, 2H), 7.59 (d, *J* = 7.8 Hz, 1H), 7.51–7.48 (m, 1H), 7.36 (d, *J* = 7.8 Hz, 2H), 4.33 (s, 2H), 3.45 (s, 3H); ^13^C NMR (150 MHz, DMSO-*d*_6_): δ 164.0, 161.5, 155.9, 146.8, 139.0, 134.2, 131.4, 128.7, 127.3, 126.8, 126.6, 126.2, 119.8, 41.1, 30.5; HR-ESIMS *m*/*z*: [M + H]^+^ calcd, 310.1186; found, 310.1177; HPLC purity = 95.9% (*t*_R_ = 2.8 min, eluted by Mobile Phase: B: 0.05% TFA in water, A: 0.05% TFA in ACN; Gradient­(T/%A): 0/3, 8.5/100, 9.0/100, 9.5/3, 10/3; Column Temp: 50 °C, Flow rate: 0.55 mL/min; Column: Acquity BEH C18 100 mm × 2.1 mm, 1.7 μm).

#### 4-[(3-Ethyl-4-oxo-3,4-dihydroquinazolin-2-yl)­methyl]-*N*-hydroxybenzamide (**6b**)

Following **General Procedure C**, compound **5b** (1.601 g, 5.0 mmol) was reacted to afford compound **6b** as a light pink solid (0.730 g, 45%). R_*f*_ = 0.32 (MeOH/DCM = 1/9); ^1^H NMR (600 MHz, DMSO-*d*_6_): δ 11.20 (br s, 1H), 9.02 (br s, 1H), 8.12 (d, *J* = 8.0 Hz, 1H), 7.80 (t, *J* = 7.4 Hz, 1H), 7.73 (d, *J* = 8.2 Hz, 2H), 7.61 (d, *J* = 8.0 Hz, 1H), 7.51 (t, *J* = 7.9 Hz, 1H), 7.40 (d, *J* = 8.3 Hz, 2H), 4.34 (s, 2H), 4.02 (q, *J* = 7.0 Hz, 2H), 1.09 (t, *J* = 7.0 Hz, 3H); ^13^C NMR (150 MHz, DMSO-*d*_6_): δ 164.5, 161.6, 155.8, 147.3, 139.9, 134.8, 131.9, 129.1, 127.8, 127.4, 127.1, 126.6, 120.6, 41.2, 14.0; HR-ESIMS *m*/*z*: [M + H]^+^ calcd, 324.1343; found, 324.1344; HPLC purity = 97.7% (*t*_R_ = 7.4 min).

#### 4-{[3-Cyclopropylquinazolin-4­(3*H*)-on-2-yl]­methyl}-*N*-hydroxybenzamide (**6c**)

Following **General Procedure C**, compound **5c** (2.420 g, 7.2 mmol) was reacted to afford compound **6c** as a yellow solid (1.890 g, 78%). *R*_f_ = 0.30 (MeOH/DCM = 1/9); mp 200–202 °C; ^1^H NMR (600 MHz, DMSO-*d*_6_): δ 11.18 (br s, 1H), 9.05 (br s, 1H), 8.05 (d, *J* = 8.4 Hz, 1H), 7.74 (m, 1H), 7.70 (d, *J* = 7.8 Hz, 2H), 7.53 (d, *J* = 7.8 Hz, 1H), 7.47–7.44 (m, 1H), 7.38 (d, *J* = 7.8 Hz, 2H), 4.44 (s, 2H), 2.69 (m, 1H), 1.18 (m, 2H), 0.91 (m, 2H); ^13^C NMR (150 MHz, DMSO-*d*_6_): δ 164.5, 162.8, 158.4, 147.0, 140.2, 134.6, 131.7, 129.5, 127.6, 127.1, 127.0, 126.5, 121.3, 41.3, 27.7, 10.8; HR-ESIMS *m*/*z*: [M + H]^+^ calcd, 336.1343; found, 336.1332; HPLC purity = 95.2% (*t*_R_ = 3.1 min, eluted by Mobile Phase: B: 0.05% TFA in water, A: 0.05% TFA in ACN; Gradient­(T/%A): 0/3, 8.5/100, 9.0/100, 9.5/3, 10/3; Column Temp: 50 °C, Flow rate: 0.55 mL/min; Column: Acquity BEH C18 100 mm × 2.1 mm, 1.7 μm).

#### 4-{[3-Cyclopropylmehtylquinazolin-4­(3*H*)-on-2-yl]­methyl}-*N*-hydroxybenzamide (**6d**)

Following **General Procedure C**, compound **5d** (0.229 g, 0.66 mmol) was reacted to afford compound **6d** as a yellow solid (47 mg, 20%). R_*f*_ = 0.20 (MeOH/DCM = 1/9); mp 217–219 °C; ^1^H NMR (600 MHz, DMSO-*d*_6_): δ 11.22 (br s, 1H), 9.05 (br s, 1H), 8.13–8.12 (m, 1H), 7.82–7.79 (m, 1H), 7.72 (d, *J* = 8.2 Hz, 2H), 7.60 (d, *J* = 8.0 Hz, 1H), 7.53–7.50 (m, 1H), 7.39 (d, *J* = 8.2 Hz, 2H), 4.38 (s, 2H), 3.95 (d, *J* = 6.9 Hz, 2H), 1.21–1.14 (m, 1H), 0.45–0.42 (m, 2H), 0.40–0.38 (m, 2H); ^13^C NMR (150 MHz, DMSO-*d*_6_): δ 164.0, 161.7, 155.5, 146.8, 139.5, 134.5, 131.4, 128.8, 127.3, 126.9, 126.8, 126.4, 120.2, 47.1, 40.7, 10.6, 3.9; HR-ESIMS *m*/*z*: [M + H]^+^ calcd, 350.1499; found, 350.1490; HPLC purity = 95.1% (*t*_R_ = 7.3 min).

#### *N*-Hydroxy-4-((4-oxo-3-phenyl-3,4-dihydroquinazolin-2-yl)­methyl)­benzamide (**6e**)

Following **General Procedure C**, compound **5e** (0.165 g, 0.44 mmol) was reacted to afford compound **6e** as a off-white solid (63 mg, 38%). R_*f*_ = 0.30 (MeOH/DCM = 1/9); mp 167–169 °C; ^1^H NMR (600 MHz, DMSO-*d*_6_): δ 11.17 (br s, 1H), 9.01 (br s, 1H), 8.12 (dd, *J* = 7.8, 1.2 Hz, 1H), 7.87–7.84 (m, 1H), 7.68 (d, *J* = 8.1 Hz, 1H), 7.58 (d, *J* = 8.2 Hz, 2H), 7.56–7.54 (m, 1H), 7.49–7.44 (m, 3H), 7.26–7.25 (m, 2H), 7.01 (d, *J* = 8.2 Hz, 2H), 3.86 (s, 2H); ^13^C NMR (150 MHz, DMSO-*d*_6_): δ 164.0, 161.5, 155.2, 147.1, 139.1, 137.0, 134.7, 131.0, 129.3, 129.0, 128.9, 128.7, 127.1, 126.9, 126.8, 126.4, 120.6, 41.6; HR-ESIMS *m*/*z*: [M + H]^+^ calcd, 372.1343; found, 372.1336; HPLC purity = 98.8% (*t*_R_ = 6.9 min).

#### 4-{[3-(2-Chlorophenyl)-4-oxo-3,4-dihydroquinazolin-2-yl]­methyl}-*N*-hydroxybenzamide (**6f**)

Following **General Procedure C**, compound **5f** (0.800 g, 2.0 mmol) was reacted to afford compound **6f** as an orange solid (0.297 g, 37%). R_*f*_ = 0.08 (MeOH/DCM = 5/95); mp 137–140 °C; ^1^H NMR (600 MHz, DMSO-*d*_6_): δ 11.17 (s, 1H), 8.99 (s, 1H), 8.13 (d, *J* = 7.2 Hz, 1H), 7.87 (m, 1H), 7.71 (m, 1H), 7.62–7.46 (m, 7H), 6.98 (d, *J* = 6.6 Hz, 2H), 3.82 (dd, *J* = 25.2, 15.0 Hz, 2H); ^13^C NMR (150 MHz, DMSO-*d*_6_): δ 163.9, 160.7, 154.6, 147.0, 138.1, 135.1, 134.3, 132.0, 131.2, 131.1, 130.1, 128.8, 128.4, 127.2, 126.8, 126.5, 120.2, 48.6, 41.3; HR-ESIMS *m*/*z*: [M + H]^+^ calcd, 406.0953; found, 406.0944; HPLC purity = 96.6% (*t*_R_ = 8.7 min).

#### 4-{[3-(4-Chlorophenyl)-4-oxo-3,4-dihydroquinazolin-2-yl]­methyl}-*N*-hydroxybenzamide (**6g**)

Following **General Procedure C**, compound **5g** (0.800 g, 2.0 mmol) was reacted to afford compound **6g** as an orange solid (0.200 g, 25%). R_*f*_ = 0.14 (MeOH/DCM = 5/95); mp 152–155 °C; ^1^H NMR (600 MHz, DMSO-*d*_6_): δ 11.16 (s, 1H), 8.98 (s, 1H), 8.11 (d, *J* = 7.8 Hz, 1H), 7.85 (m, 1H), 7.67 (d, *J* = 7.8 Hz, 1H), 7.59 (d, *J* = 8.4 Hz, 2H), 7.54 (m, 1H), 7.50 (d, *J* = 8.4 Hz, 2H), 7.29 (d, *J* = 8.4 Hz, 2H), 7.03 (d, *J* = 7.8 Hz, 2H), 3.87 (s, 2H); ^13^C NMR (150 MHz, DMSO-*d*_6_): δ 163.9, 161.5, 154.9, 147.0, 138.9, 135.9, 134.7, 133.6, 131.0, 130.8, 129.2, 128.7, 127.1, 127.0, 126.8, 126.3, 120.5, 54.9, 41.6; HR-ESIMS *m*/*z*: [M + H]^+^ calcd, 406.0953; found; 406.0938; HPLC purity = 97.4% (*t*_R_ = 8.8 min).

#### 4-((3-(2,6-Dimethylphenyl)-4-oxo-3,4-dihydroquinazolin-2-yl)­methyl)-*N*-hydroxybenzamide (**6h**)

Following **General Procedure C**, compound **5h** (0.800 g, 2.0 mmol) was reacted to afford compound **6h** as a yellow solid (0.335 g, 42%). R_*f*_ = 0.09 (MeOH/DCM = 5/95); mp 133–136 °C; ^1^H NMR (600 MHz, DMSO-*d*_6_): δ 11.16 (s, 1H), 8.97 (s, 1H), 8.14 (d, *J* = 7.8 Hz, 1H), 7.89 (m, 1H), 7.75 (d, *J* = 7.8 Hz, 1H), 7.59 (d, *J* = 8.4 Hz, 2H), 7.56 (m, 1H), 7.35 (m, 1H), 7.21 (7.8 Hz, 2H), 6.93 (d, *J* = 8.4 Hz, 2H), 3.71 (s, 2H), 1.71 (s, 6H); ^13^C NMR (150 MHz, DMSO-*d*_6_): δ 163.8, 160.3, 154.9, 147.2, 137.8, 135.5, 135.0, 131.4, 129.3, 129.0, 128.7, 127.2, 127.1, 126.7, 126.5, 120.1, 54.9, 41.0, 17.0; HR-ESIMS *m*/*z*: [M + H]^+^ calcd, 400.1656; found, 400.1645; HPLC purity = 99.3% (*t*_R_ = 8.9 min).

#### *N*-Hydroxy-4-{[3-(4-methoxyphenyl)-4-oxo-3,4-dihydroquinazolin-2-yl]­methyl}­benzamide (**6i**)

Following **General Procedure C**, compound **5i** (0.800 g, 2.0 mmol) was reacted to afford compound **6i** as an orange solid (0.152 g, 19%). R_*f*_ = 0.11 (MeOH/DCM = 5/95); mp 214–215 °C; ^1^H NMR (600 MHz, DMSO-*d*_6_): δ 11.15 (s, 1H), 8.97 (s, 1H), 8.10 (dd, *J* = 7.8, 1.2 Hz, 1H), 7.83 (m, 1H), 7.65 (d, *J* = 7.8 Hz, 1H), 7.59 (d, *J* = 8.4 Hz, 2H), 7.53 (m, 1H), 7.14 (d, *J* = 9.0 Hz, 2H), 7.03 (d, *J* = 8.4 Hz, 2H), 6.97 (d, *J* = 9.0 Hz, 2H), 3.86 (s, 2H), 3.80 (s, 3H); ^13^C NMR (150 MHz, DMSO-*d*_6_): δ 164.0, 161.7, 159.3, 155.7, 147.0, 139.1, 134.6, 131.0, 129.9, 129.5, 128.68, 128.67, 127.0, 126.7, 126.4, 120.6, 114.4, 55.4, 41.5; HR-ESIMS *m*/*z*: [M + H]^+^ calcd, 402.1448; found, 402.1436; HPLC purity = 99.0% (*t*_R_ = 8.2 min).

#### 4-[(6-Chloro-3-methyl-4-oxo-3,4-dihydroquinazolin-2-yl)­methyl]-*N*-hydroxybenzamide (**6j**)

Following **General Procedure C**, compound **5j** (0.420 g, 1.2 mmol) was reacted to afford compound **6j** as an orange solid (0.184 g, 44%). R_*f*_ = 0.31 (MeOH/DCM = 1/9); mp 214–216 °C; ^1^H NMR (600 MHz, DMSO-*d*_6_): δ 11.19 (s, 1H), 9.01 (s, 1H), 8.01 (d, *J* = 2.4 Hz, 1H), 7.78 (dd, *J* = 8.4, 2.4 Hz, 1H), 7.72 (d, *J* = 8.4 Hz, 2H), 7.60 (d, *J* = 9.0 Hz, 1H), 7.36 (d, *J* = 8.4 Hz, 2H), 4.33 (s, 2H), 3.45 (s, 3H); ^13^C NMR (150 MHz, DMSO-*d*_6_): δ 164.0, 160.6, 156.6, 145.5, 138.8, 134.3, 131.4, 130.7, 129.1, 128.8, 127.2, 125.1, 121.0, 41.0, 30.6; HR-ESIMS *m*/*z*: [M + H]^+^ calcd, 344.0796; found, 344.0787; HPLC purity = 95.6% (*t*_R_ = 8.2 min).

#### 4-[(6-Chloro-3-cyclopropyl-4-oxo-3,4-dihydroquinazolin-2-yl)­methyl]-*N*-hydroxybenzamide (**6k**)

Following **General Procedure C**, compound **5k** (0.800 g, 2.2 mmol) was reacted to afford compound **6k** as an orange solid (0.090 g, 11%). R_*f*_ = 0.2 (MeOH/DCM = 1/9); mp 240–243 °C (dec.); ^1^H NMR (600 MHz, DMSO-*d*_6_): δ 11.18 (s, 1H), 9.01 (s, 1H), 7.98 (s, 1H), 7.76 (d, *J* = 8.4 Hz, 1H), 7.70 (d, *J* = 7.2 Hz, 2H), 7.55 (d, *J* = 7.8 Hz, 1H), 7.38 (d, *J* = 7.2 Hz, 2H), 4.44 (s, 2H), 2.71 (s, 1H), 1.19 (s, 1H), 0.92 (s, 1H); ^13^C NMR (150 MHz, DMSO-*d*_6_): δ 164.1, 161.3, 158.6, 145.2, 139.5, 134.2, 131.2, 130.6, 129.1, 128.9, 127.1, 125.0, 122.0, 40.8, 27.3, 10.2; HR-ESIMS *m*/*z*: [M + H]^+^ calcd, 370.0953; found, 370.0942; HPLC purity = 95.6% (*t*_R_ = 8.6 min).

#### 4-[(6-Fluoro-3-methyl-4-oxo-3,4-dihydroquinazolin-2-yl)­methyl]-*N*-hydroxybenzamide (**6l**)

Following **General Procedure C**, compound **5l** (0.800 g, 2.5 mmol) was reacted to afford compound **6l** as a white solid (0.280 g, 35%). R_*f*_ = 0.07 (MeOH/DCM = 5/95); mp 226–228 °C; ^1^H NMR (600 MHz, DMSO-*d*_6_): δ 11.18 (s, 1H), 9.01 (s, 1H), 7.77 (d, *J* = 8.4 Hz, 1H), 7.71 (d, *J* = 8.4 Hz, 2H), 7.67 (m, 2H), 7.36 (d, *J* = 8.4 Hz, 2H), 4.33 (s, 2H), 3.45 (s, 3H); ^13^C NMR (150 MHz, DMSO-*d*_6_): δ 164.1, 161.0, 160.0 (d, *J* = 243.8 Hz), 155.5, 143.8, 139.0, 131.4, 129.8 (d, *J* = 8.1 Hz), 128.8, 127.3, 122.9 (d, *J* = 24.0 Hz), 121.0 (d, *J* = 8.4 Hz), 110.7 (d, *J* = 23.3 Hz), 41.0, 30.7; HR-ESIMS *m*/*z*: [M + H]^+^ calcd, 328.1092; found, 328.1086; HPLC purity = 98.0% (*t*_R_ = 7.3 min).

#### 4-[(6-Cyano-3-methyl-4-oxo-3,4-dihydroquinazolin-2-yl)­methyl]-*N*-hydroxybenzamide (**6m**)

To a solution of **5e** (0.703 g, 2.1 mmol) in NMP (6 mL) was added NaCN (0.200 g, 4.1 mmol) and NiBr_2_ (0.448 g, 2.1 mmol). The mixture was irradiated with microwave (closed-vessel, 120 W) at 200 °C for 10 min. Upon completion, the reaction mixture was partitioned between EA/H_2_O (50/30 mL) and washed with H_2_O (50 × 2 mL). The EA layer was dried over MgSO_4_ and concentrated in vacuo. The residue was purified by flash column chromatography (silica gel, EA/*n*-heptane = 0/1 to 1/1), and the desired fractions were collected to afford the acid intermediate as a pink solid (0.210 g, 32%). To a solution of the resulting acid (0.210 g, 0.7 mmol) in DMF (5 mL) was added EDCI·HCl (0.420 g, 2.2 mmol), HOBt hydrate (0.154 mg, 1.0 mmol), and stirred at ambient temperature for 10 min. Then, NH_2_OBn·HCl (0.327 g, 2.1 mmol) and DIPEA (0.36 mL, 2.1 mmol) was added to the mixture and stirred at rt for another 2 h. Upon completion, the reaction mixture was poured into H_2_O (100 mL) and the resulting precipitate was filtered to afford the OBn protected intermediate as an orange solid (0.214 g, 77%). To a suspension of the OBn protected compound (0.214 g, 0.5 mmol) in DCM (anhydrous, 10 mL) was added BBr_3_ dimethyl sulfide complex (1 M in DCM, 1 mL) at 0 °C, and the reaction mixture was stirred for 30 min. Upon completion, the reaction mixture was quenched by water (50 mL), extracted with DCM (20 × 3 mL). The combined organic layers were dried over MgSO_4_ and concentrated in vacuo. The crude product was purified by flash column chromatography (silica gel, MeOH/DCM = 0/1 to 1/25), and the desired fractions were collected to afford compound **6m** as a pink solid (0.024 g, 21%). R_*f*_ = 0.08 (MeOH/DCM = 5/95); ^1^H NMR (600 MHz, DMSO-*d*_6_): δ 11.19 (s, 1H), 9.02 (s, 1H), 8.50 (s, 1H), 8.12 (d, *J* = 8.4 Hz, 1H), 7.71 (m, 3H), 7.37 (d, *J* = 7.8 Hz, 2H), 4.36 (s, 2H), 3.47 (s, 3H); ^13^C NMR (150 MHz, DMSO-*d*_6_): δ 164.0, 160.5, 159.3, 149.4, 138.5, 136.4, 131.9, 131.4, 128.9, 128.3, 127.3, 120.3, 118.2, 108.7, 41.2, 30.8; HR-ESIMS *m*/*z*: [M + H]^+^ calcd, 335.1139; found, 335.1129; HPLC purity = 95.2% (*t*_R_ = 7.1 min).

### HDAC Enzyme Activity Assays

The HDACs inhibition assays were performed by Reaction Biology Corporation (Malvern, PA) using full length recombinant hHDACs that had been expressed in Sf9 cells via baculovirus vectors. The test compounds were dissolved in DMSO to make a 30 μM stock, followed by being tested in 10-dose IC50 mode with 3-fold serial dilutions starting at 10 or 30 μM. The enzyme was diluted in the reaction buffer (50 nM Tris–HCl of pH 8.0, 137 mM NaCl, 2.7 mM KCl, 1 mM MgCl_2_, 1 mg/mL BSA, 1% DMSO), and then the test compound and specific substrate were delivered into the reaction mixture by sequence. The reaction was stopped after 2 h-incubation at 30 °C. Trichostatin A was used as the internal control. The substrate I, a fluorogenic peptide from p53 residues 379–382 (RHKKAc, 50 μM) is used for all HDAC1 to 11 but HDAC8, which used substrate II, a fluorogenic diacylpeptide based on residues 379–382 of p53 (RHKAcKAc, 50 μM).

### Immunoblotting

Cell-based HDACs inhibition assays were performed by Reaction Biology Corporation (Malvern, PA). HepG2 cells (American Type Culture Collection, Manassas, VA) were grown in Eagle’s Minimum Essential Medium (EMEM). All media were supplemented with 10% FBS, 100 μg/mL penicillin, and 100 μg/mL streptomycin. Cultures were maintained at 37 °C in a humidified atmosphere of 5% CO_2_ and 95% air, and the procedures were described as follow. Briefly, 5 × 10^5^ cells/well of HepG2 cells were seeded in 24-well plates with complete culture media overnight. The media was removed and 950 μL of fresh culture media and 50 μL of tested compounds were added to the cells. After culturing for 16 h, the cells were washed with ice cold PBS and lysed with 1x SDS buffer supplemented with 50 mM DTT. The resulting samples were homogenized, spun down, and heated to 95 °C for 5 min. Cell lysate samples were then separated by SDS-PAGE on 12% Bis-Tris gel (Thermo Fisher Scientific), transferred onto nitrocellulose membranes (iBlot Dry Blotting system, Life Technologies), and then blocked in a blocking buffer (3% milk solution) for 1 h. Membranes were incubated overnight with Acetyl-H3K9 (Cell Signaling Technology, CST#9649S), Histone H3 (Cell Signaling Technology, CST#3638S), Acetyl-Tubulin (Sigma-Aldrich, #T7451), and α-Tubulin (Sigma-Aldrich, #T9026) antibodies. Blots were developed using LI-COR antirabbit IgG IRDye 680RD and antimouse IgG IRDye 800CW secondary antibodies (LI-COR Biosciences). The membranes were scanned with LI-COR Odyssey Fc Imaging System, and the specific bands of interest were quantified by LI-COR Image Studio Lite software.

### BioMAP Fibrosis Panel

BioMAP Fibrosis Panel was commercially available service (Eurofins DiscoverX Corporation, Freemont, CA, USA). GV-001 was tested in these assays at four concentrations: 10 μM, 3.3 μM, 1.1 μM and 370 nM. Human primary cells in BioMAP systems are used at early passage (passage 4 or earlier) to minimize adaptation to cell culture conditions and preserve physiological signaling responses. All human primary cells were obtained under protocols that were reviewed by Institutional Review Board(s) (IRB) that operate in accordance with the requirement of the EPA Regulation 40 CFR 26 and HHS Regulation 45 CFR 46 of the US Department of Health and Human Resources for the protection of human research subjects. All primary cells are pooled from multiple donors (*n* = 3–6), commercially purchased and handled according to the recommendations of the manufacturers. The three systems in the Fibrosis panel are stimulated for 48 h with a cocktail of TNF-α and TGF-β. Cell types used in each system are as follows: MyoF system [differentiated lung myofibroblasts], and REMyoF system [renal proximal tubule epithelial cells and differentiated lung myofibroblasts]. Adherent cell types are cultured in 96-well plates until confluent, followed by the addition of PBMC. Test agents prepared in DMSO (final concentration ≤0.1%) are added at the specified concentrations 1 h before stimulation and remain in culture for 48 h. Each plate contains drug controls (e.g., legacy control test agent colchicine), negative controls (e.g., nonstimulated condition) and vehicle controls (e.g., 0.1% DMSO). Direct ELISA is used to measure biomarker levels of cell-associated and cell membrane targets. Soluble factors from supernatants are quantified using either HTRF detection, multiplex electrochemiluminescence assay or capture ELISA. Effects of test agents on cell viability (cytotoxicity) are measured by sulforhodamine B (SRB) for adherent cells (48 h). All test agents are tested in standardized formats at 4 concentrations in triplicate. Colchicine stimulated wells, vehicle control treated wells, and wells without stimulation are included as controls on each plate (*n* = 3–8). Data acceptance criteria are based on plate performance (% CV of controls) and the performance of controls across assays with a comparison to historical controls. Additional information can be found in previous descriptions.

### *In Vitro* 3D ADPKD Cyst Assay

The ADPKD cyst assays were performed by Discovery BioMed (DBM, now Eurofins Discovery). Human ADPKD kidney tissues and primary cells were obtained from DBM, which sources organs not suitable for transplantation through commercial tissue procurement networks. All specimens are provided in a deidentified form, with only limited demographic and infectious disease screening data available to the vendor. According to U.S. federal regulation 45 CFR 46 and associated guidance, research using these deidentified human materials is exempt from Institutional Review Board (IRB) review. The efficacy of compound **6a** and **6c** in reducing cyst formation and growth was evaluated using a DBM Prevention Assay in a 3D in vitro model of ADPKD. Both compounds were evaluated at seven concentrations: 0.1, 0.3, 1, 3, 10, 30, and 90 μM. The experimental design included a DMSO control at 0.1% and stimulation with 1 μM Forskolin. Cells were seeded in 384-well plates and treated with the compounds over a 12 day period with end points including imaging and assays performed on day 14. For the prevention assay, cells will be seeded on day 0 and treated on day 1, whereas additional treatments will be added every 3–4 days throughout the study. For the reduction assay, drug treatments and precise experimental timing will be based on cyst growth, and compounds will be added after cyst have begun to form, approximately on day 4–7. Additional treatments will be added every 2 days throughout the study. Cell viability was measured using CellTiterGlo (CTG) to assess ATP content, while lactate dehydrogenase (LDH) levels were measured to evaluate cytotoxicity. The impact on cystogenesis was determined by imaging to analyze cyst number and size.

### ADPKD Mouse Model

C57BL/6 Tg­(Ubi-emGFP Pkd1miRNA)­1SJi/Narl mice were obtained from National Rodent Model Resource Center (Tainan, Taiwan) and were housed on a regular 12 h light/dark cycle and had free access to food and water. The study procedures and protocols were approved by National Laboratory Animal Center for Institutional Animal Care and Use Committee (IACUC approved number NLAC­(TN)-110-D-006-R4). Animals were quarantined and acclimated for at least 3 days prior to dose initiation. The mutant mice express miRNA hairpins specific to *Pkd1* transcript, resulting in stable and heritable *Pkd1* knockdown and progressive renal cystogenesis as described in detail previously. ^45–47^ Fourteen-day-old *Pkd1* miRNA transgenic mice of males and females were randomized into three groups (*n* = 14–15 in each group). Mice received oral administration of GV-001 at 60 mg/kg (Group 2) or 120 mg/kg (Group 3) in 0.5% methylcellulose once daily for 14 days, from P15 to P28. The vehicle control group was treated with 0.5% Methocel A4M through daily oral administration (Group 1). Mice were anesthetized and sacrificed after 14 day dosing. Both right and left kidneys were harvested and the weights were measured.

### Histopathology

The semiquantitative analysis of histopathology was performed by LASCO (BioLasco Taiwan Co. Ltd., Taipei, Taiwan). During necropsy, the kidney tissue was preserved in 10% neutral buffered formalin (NBF). The kidney tissue was trimmed, embedded in paraffin, sectioned, stained with Masson’s trichrome staining, and examined microscopically by the veterinary pathologist. The cross-section was examined by the veterinary pathologist using optical microscope. The cyst area percentage was calculated in five consecutively selected fields at 100× magnification.

### *In Vitro* ADME Evaluations of Plasma, Hepatocyte, and Liver Microsome Stability

All in vitro ADME studies were performed at WuXi AppTec Laboratory Testing Division, New Jersey, US. For plasma stability study, compounds (2.0 μM) were incubated with CD-1 mouse, SD rat, beagle dog, cynomolgus monkey and human plasma at 37 °C for up to 120 min. Concentrations of remaining compounds in the incubation samples were determined by semiquantitation with LC–MS/MS. For hepatocyte stability study, compounds (1.0 μM) were incubated with mouse, rat, dog, monkey and human hepatocytes (1 × 10^6^ cells/mL) at 37 °C in a humidified incubator with 5% CO_2_ for 0, 15, 30-, 60-, 90- and 120 min. Remaining compound was analyzed by LC–MS/MS semiquantitatively. For liver microsome stability study, compounds (10 μM) were incubated with male mouse, rat, dog, monkey, and mixed gender human liver microsomes (ca. 1 mg/mL) at ca. 37 °C for 0 and 60 min. The incubations were terminated by the addition of ice-cold quench solution. Positive control, 7-ethoxycoumarin (7-EC) at 100 μM, was used to evaluate the functionality of liver microsomes. The supernatants (2 mL aliquot) of the liver microsomal incubations were evaporated to dryness under nitrogen. The dry residues were reconstituted in ca. 0.2 mL ethanol/water (1:1, v/v), followed by centrifugation at 10,000*g* and 4 °C for 10 min. The samples were analyzed using high resolution LC/MS for metabolite search and identification.

### Pharmacokinetics (PK) in Rats and Mice

Rat and mouse PK studies were performed at BioDuro (Shanghai) Co., Ltd. The study and all experimental procedures were reviewed and approved by the BioDuro (Shanghai) Institutional Animal Care and Use Committee (IACUC, protocol No. BD-202006221). During the in-life portion of the study, animals were housed in cages. The room(s) were controlled and monitored for relative humidity (targeted mean range 40% to 70%) and temperature (targeted mean range 20 to 26 °C) with no less than 15 times air changes/hour. The room(s) were on a 12 h light/dark cycle except when interruptions were necessitated by study activities. Rat or mice were dosed with compounds following a single IV administration at 1.00 mg/kg with a dosing volume of 2.00 mL/kg, formulated in the vehicle of 10% 1-methyl-2-pyrrolidone/10% Kolliphor HS-15/80% normal saline. Oral administration at 30 mg/kg or 60 mg/kg with a dosing volume of 10.0 mL/kg, using a vehicle consisting of (a) 0.5% benzyl alcohol/29.5% PEG-300/70% propylene glycol and 0.4% sucralose (Table 4), or (b) 0.5% methylcellulose (Table S1). Blood (∼0.6 mL per time point) was collected from Saphenous vein into K2EDTA tubes. The sampling time-points were predose, 0.0833, 0.25, 0.5, 1, 2, 4, 8, and 24 h for the IV group and predose, 0.25, 0.5, 1, 2, 4, 8, and 24 h for the PO group. Immediately following blood collection, the samples were inverted several times and put on wet ice prior to centrifugation. Within 30 min after collection, plasma was separated by centrifugation at 4600 rpm for 5 min at 4 °C. The measured plasma concentrations at each time-point, the nominal sample collection time, and dosages were used for PK analysis. The PK parameters were determined by noncompartmental analysis using WinNonlin Version 8.0. Pharmacokinetic profiles were determined for each individual animal.

## Supplementary Material





## Notes

Molecular formula string (CSV)
IC_50_ measurement of Ac-α-tubulin and Ac-H3 protein expression (Figure S1); preventive assay evaluating effects of compounds **6a** and **6c** (Figure S2); reductive assay evaluating effects of compounds **6a** and **6c** (Figure S3); ^1^H NMR and ^13^C NMR spectra; and HPLC chromatograms of biologically tested compounds (PDF)

## Abbreviations

ADPKD: autosomal dominant polycystic kidney disease
ARB: angiotensin II receptor blocker
ECM: extracellular matrix
EMT: epithelial–mesenchymal transition
ESRD: end-stage renal disease
H&E: hematoxylin and eosin
HDAC: histone deacetylase
IHC: immunohistochemical
LDH: lactate dehydrogenase
MyoF: modeling fibrosis
PC1: polycystin-1
PC2: polycystin-2
RAS: renin-angiotensin system
REMyoF: modeling kidney fibrotic disease
SRB: sulforhodamine B
TGF-β: transforming growth factor β
TNF-α: tumor necrosis factor α
TSA: trichostatin A
ZBG: zinc binding group.
