# Supplementary material for: GV-001, An Oral Available Histone Deacetylase 6 Inhibitor for the Treatment of Autosomal Dominant Polycystic Kidney Disease
Source: J Med Chem. 2026 Apr 6;69(9):10122–39. doi: 10.1021/acs.jmedchem.5c03139 (PMC13181768; doi:10.1021/acs.jmedchem.5c03139)
Supplement: Supplementary file 2 [file jm5c03139_si_002.pdf]

Supporting Information

GV-001, An Oral Available Histone Deacetylase 6 Inhibitor for the Treatment of Autosomal Dominant Polycystic Kidney Disease

*Sian-Siou Wu[a, #], Pei-Yun Hung[b, #], Tsung-Yu Yeh[a], Po-Jui Lin[a], Ji-Wang Chern[b], and Chao-Wu, Yu[a, *]*

[a]School of Pharmacy, College of Medicine, National Taiwan University, Taipei 100, Taiwan

[b]Gilva Therapeutics Co., Ltd., Taipei 100, Taiwan

**E-mail: stifenyu@ntu.edu.tw*

Table of Contents

Table S1. Mouse PK Parameters used for dose selection in the *in vivo* efficacy studies **S3**

Figure S1. IC_{50} measurement of Ac-α-tubulin and Ac-H3 protein expression **S4**

Figure S2. Preventive assay evaluating effects of compounds **6a** and **6c** **S5**

Figure S3. Reductive assay evaluating effects of compounds **6a** and **6c** **S6**

^{1}H NMR and ^{13}C NMR spectra **S7**

HPLC chromatograms of biologically tested compounds **S44**

Table S1. Mouse PK Parameters used for dose selection in the *in vivo* efficacy studies

Dose Route and Dosage	PO 60 mg/kg[a]
C_{max} (ng/mL)	115 ± 35
$T_{1/2}$ (h)	3.33 ± 2.94
T_{max} (h)	0.917 ± 0.946
AUC_{last} (h·ng/mL)	408 ± 24.0
AUC_{inf} (h·ng/mL)	574 ± 215

[a]Vehicle: 0.5% methylcellulose.

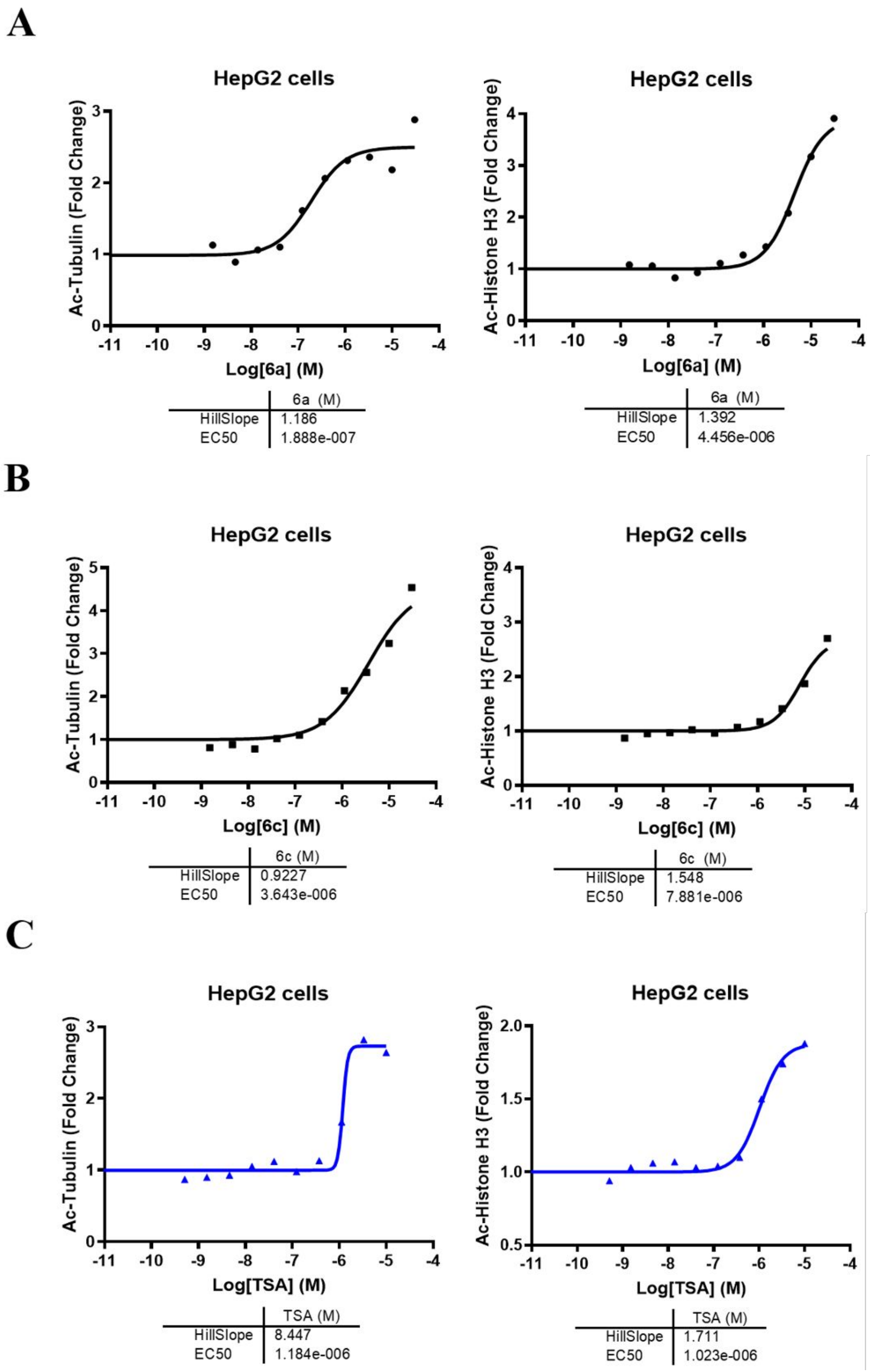

Figure S1. IC_{50} measurement of Ac-α-tubulin and Ac-H3 protein expression detected by Western blotting. HepG2 cells were treated with **6a** (A), **6c** (B), and TSA (C) at various concentrations. Statistical analysis was performed under GraphPad Prism with nonlinear regression.

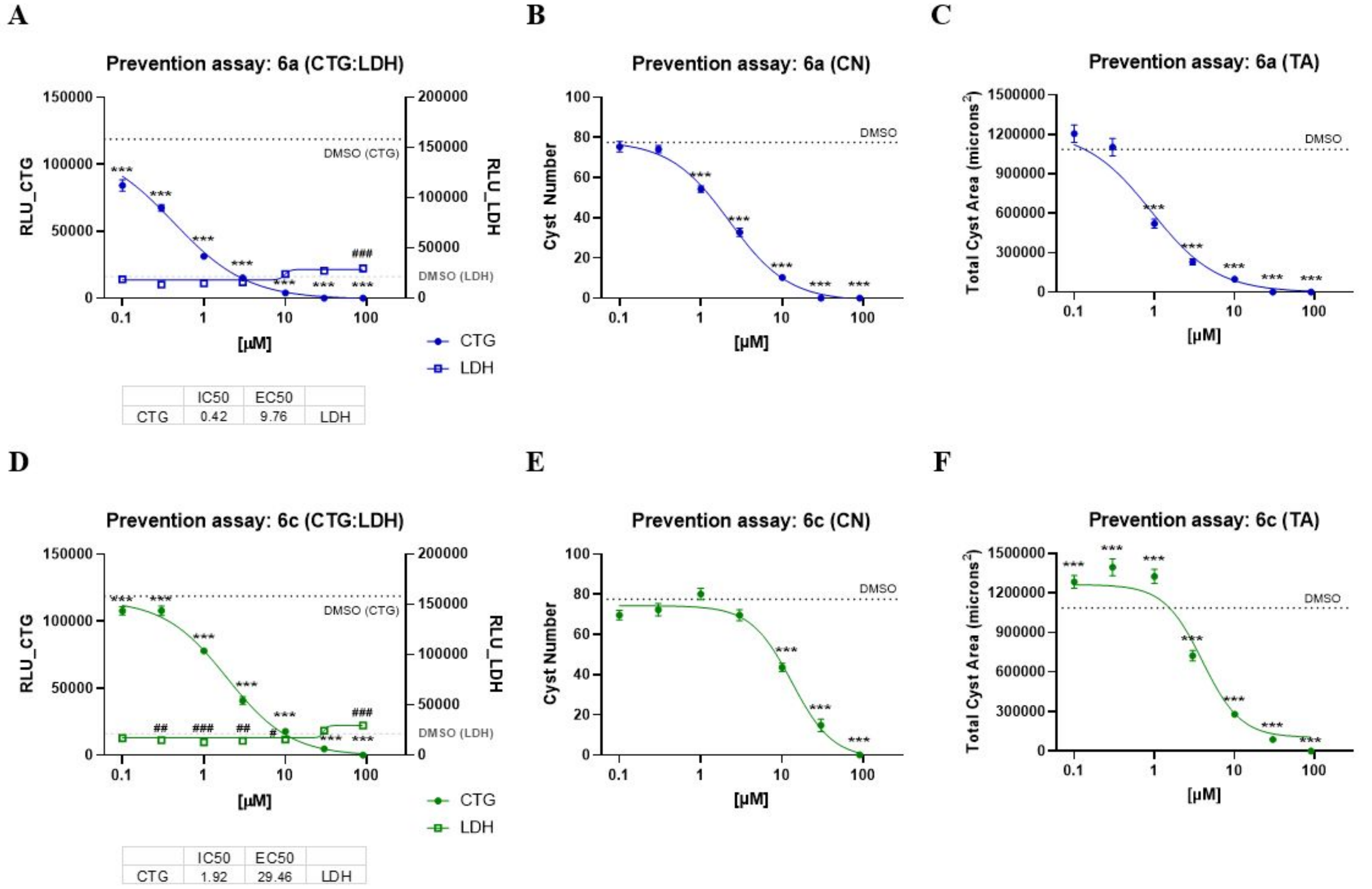

Figure S2. Prevention assay evaluating effects of compounds (A–C) **6a** and (D–F) **6c** on cyst cell viability (measured by CTG), cytotoxicity (measured by LDH), cyst number (CN), and total cyst area (TA) in a 3D ADPKD model, presented as concentration–response curves. CTG and LDH values were represented as raw luminescent units (RLU), where drug toxicity was observed after the curves intersect and when significance was indicated. All data are presented as mean ±S.E.M.; *$p < 0.05$; ***$p < 0.001$; n = 10; statistical significance between the DMSO control and treated groups was determined using one-way ANOVA followed by Dunnet's multiple comparisons test.

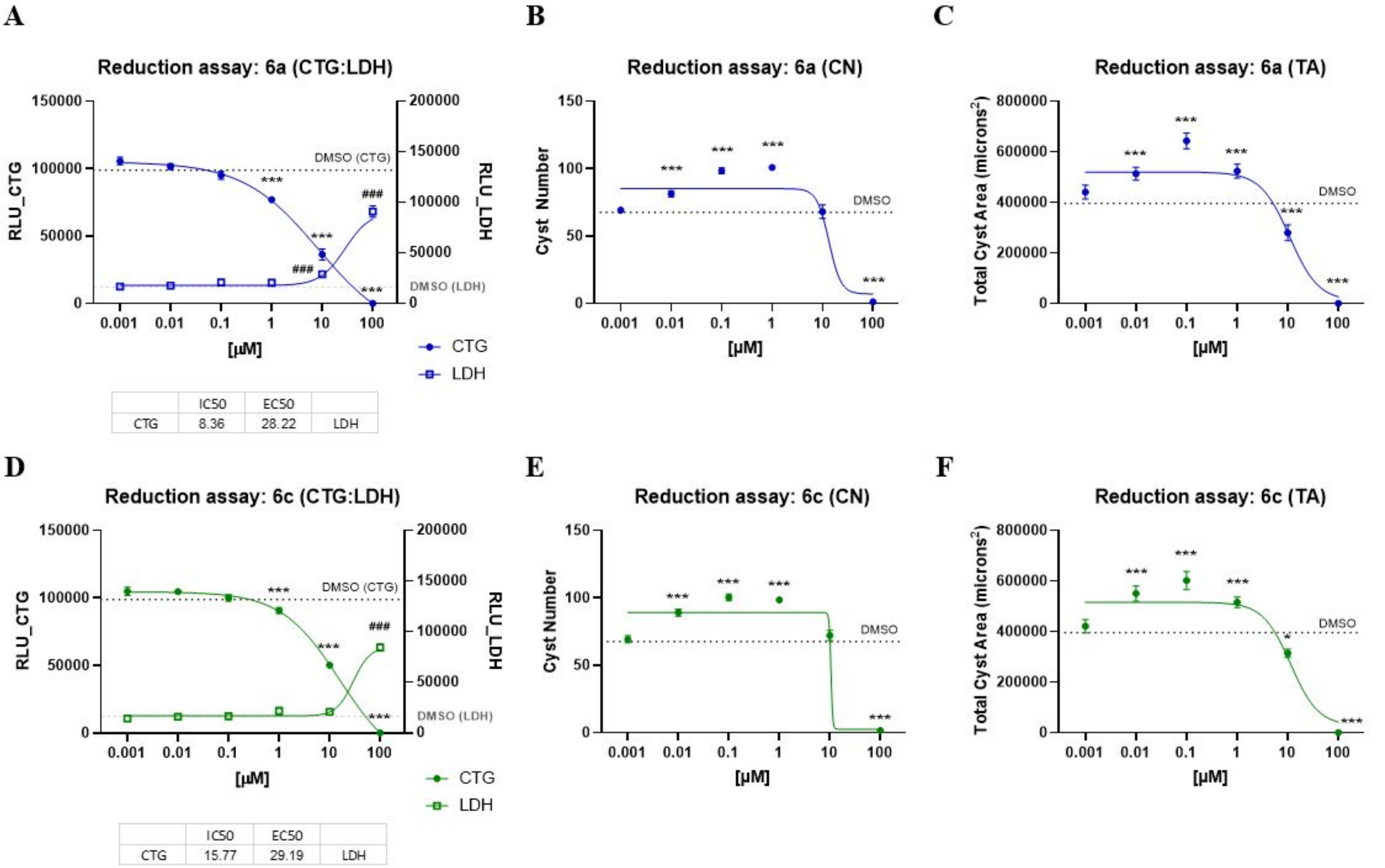

Figure S3. Reduction assay evaluating effects of compounds (A–C) **6a** and (D–F) **6c** on cyst cell viability (measured by CTG), cytotoxicity (measured by LDH), cyst number (CN), and total cyst area (TA) in a 3D ADPKD model, presented as concentration–response curves. CTG and LDH values were represented as raw luminescent units (RLU), where drug toxicity was observed after the curves intersect and when significance was indicated. All data are presented as mean ± S.E.M.; *$p < 0.05$; ***$p < 0.001$; n = 10; statistical significance between the DMSO control and treated groups was determined using one-way ANOVA followed by Dunnet's multiple comparisons test.

^{1}H NMR spectrum of compound **4a**

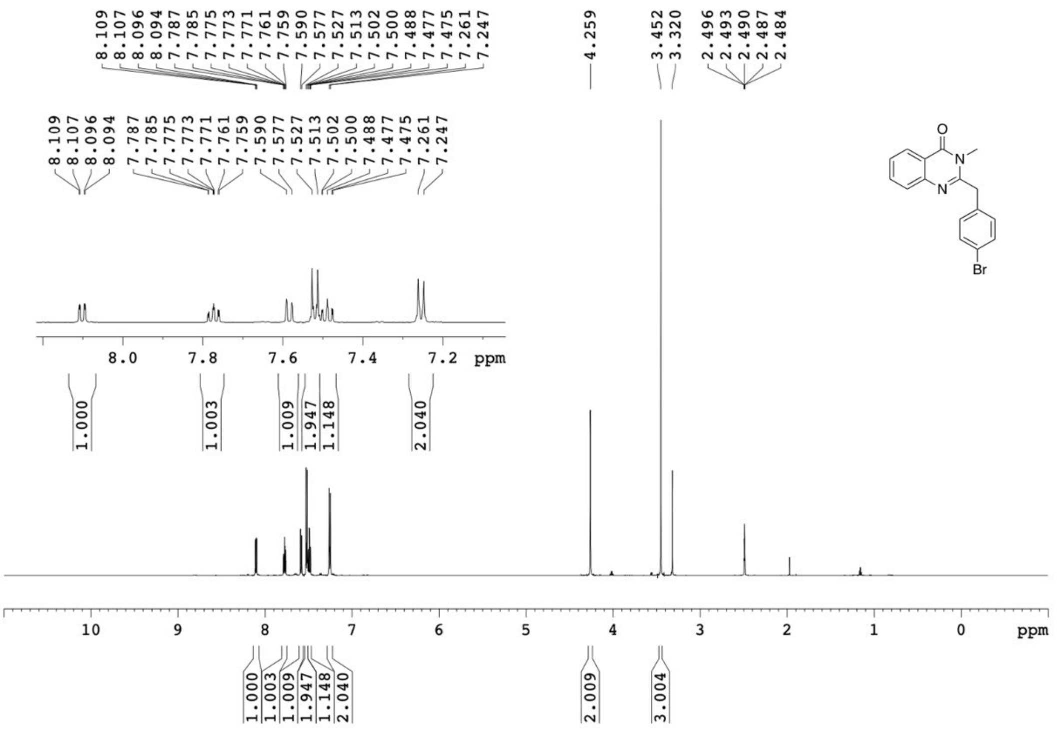

^{13}C NMR spectrum of compound **4a**

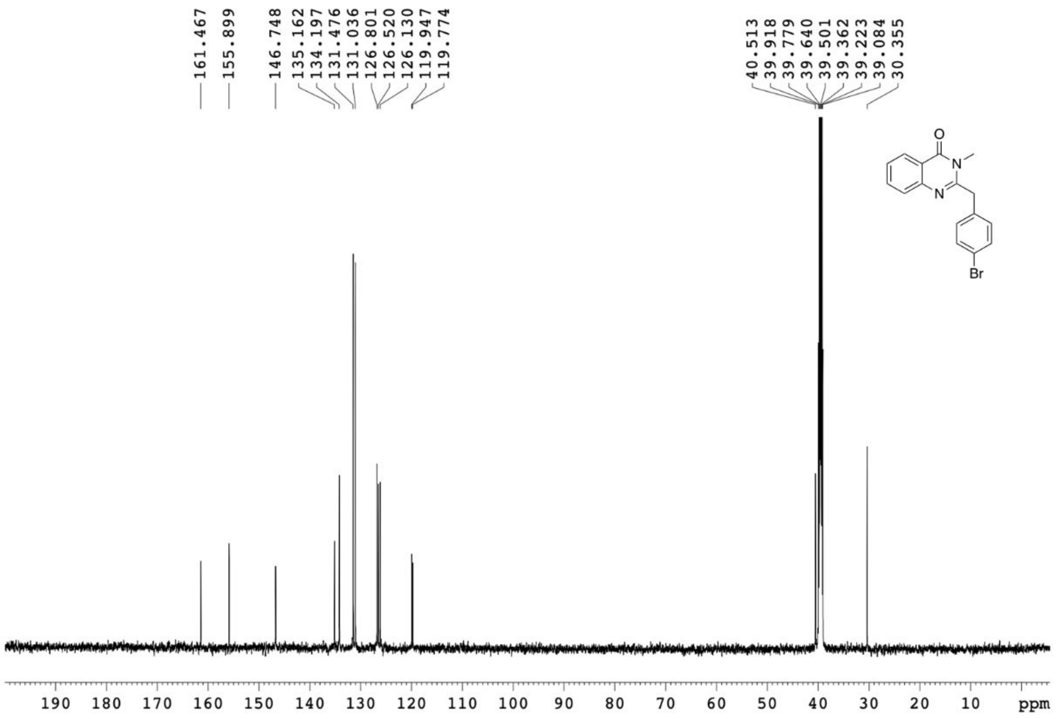

^{1}H NMR spectrum of compound **4b**

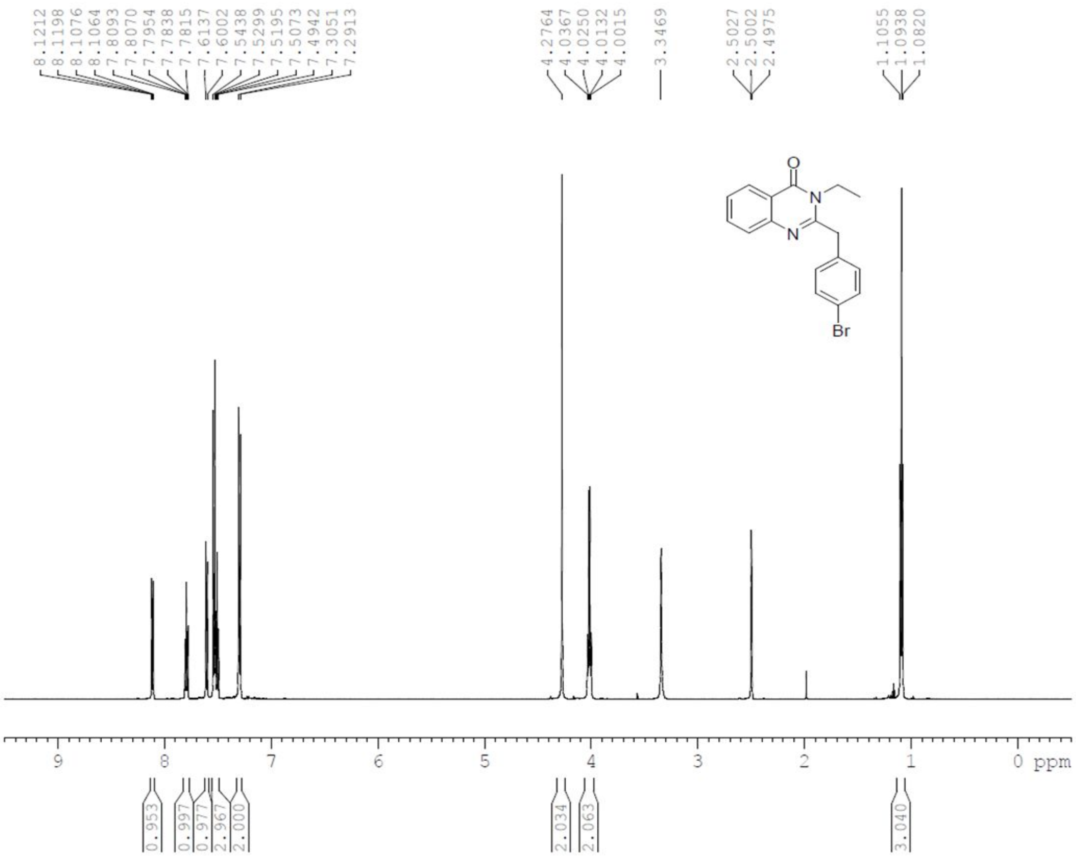

^{13}C NMR spectrum of compound **4b**

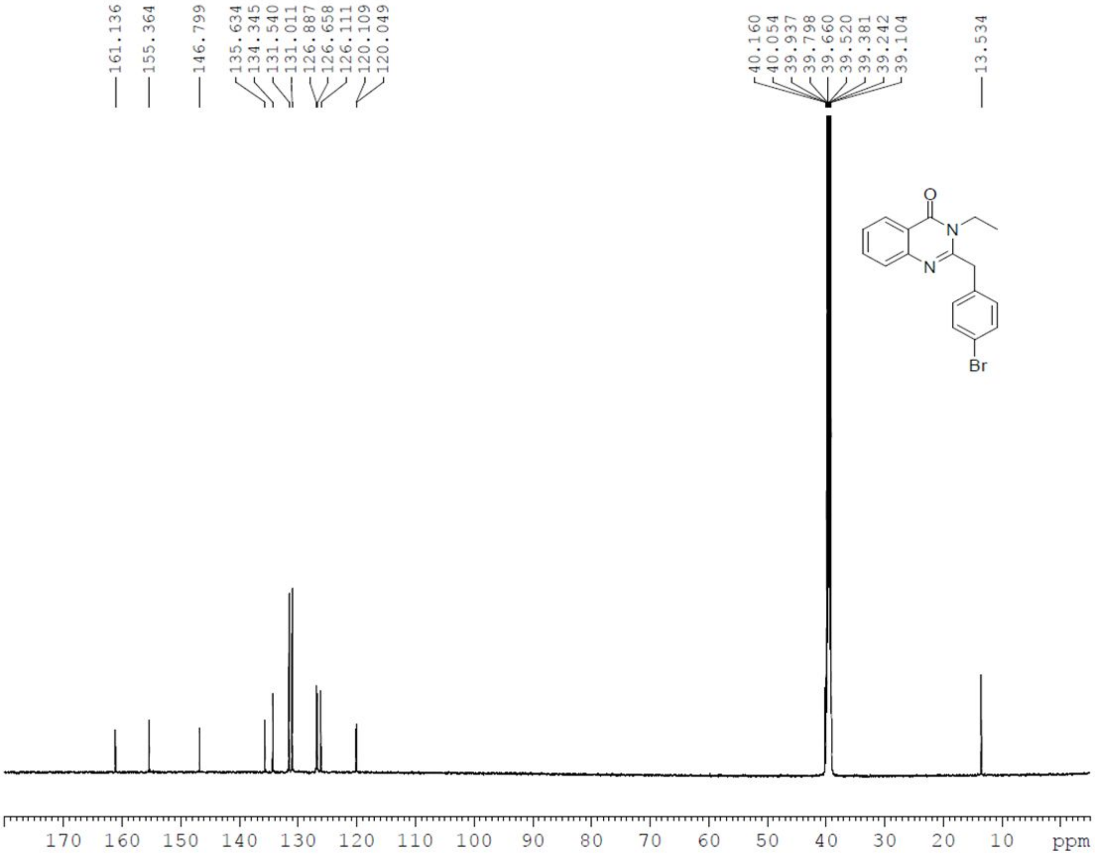

^{1}H NMR spectrum of compound **4c**

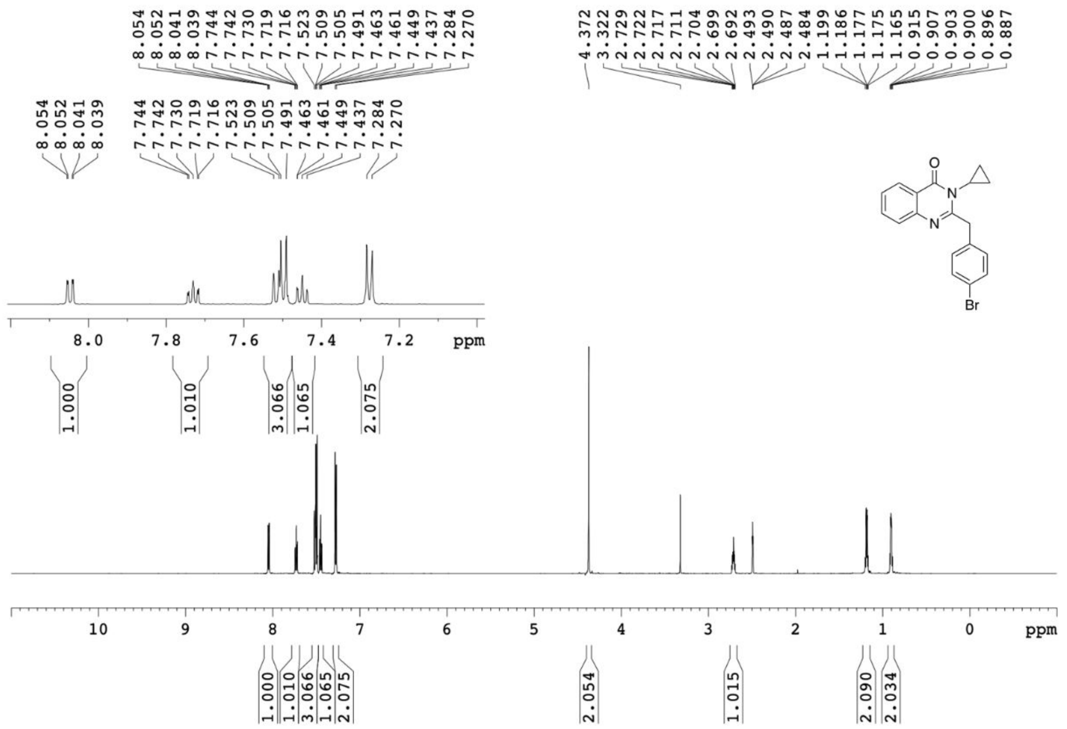

^{13}C NMR spectrum of compound **4c**

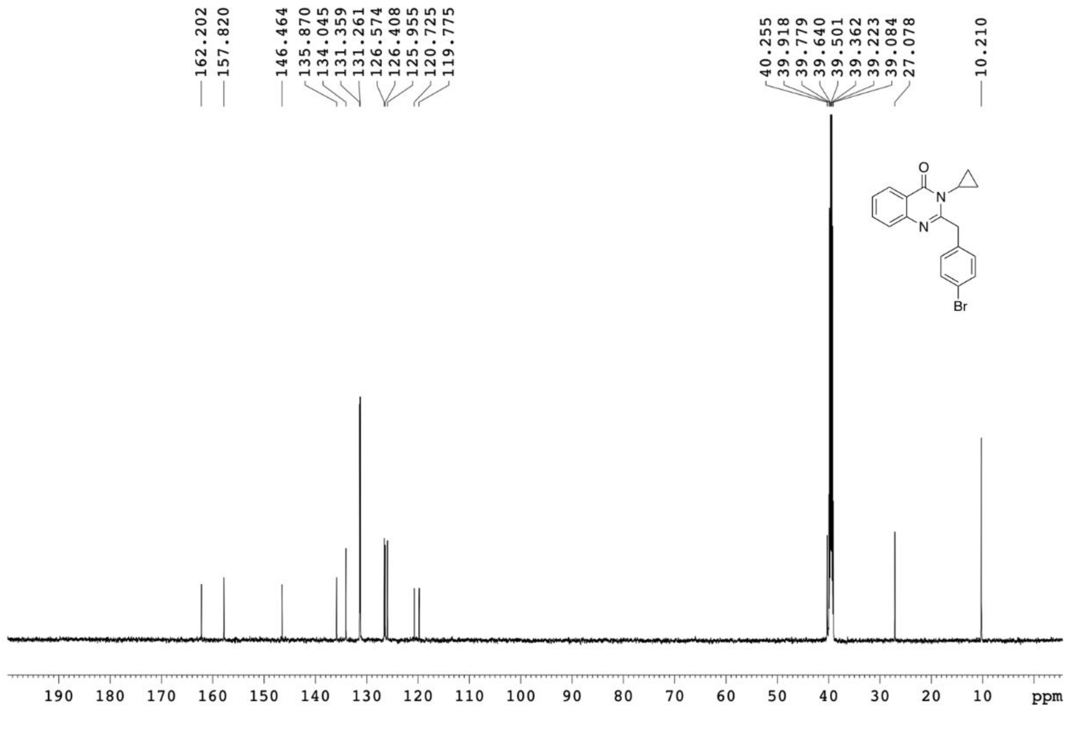

^{1}H NMR spectrum of compound **4d**

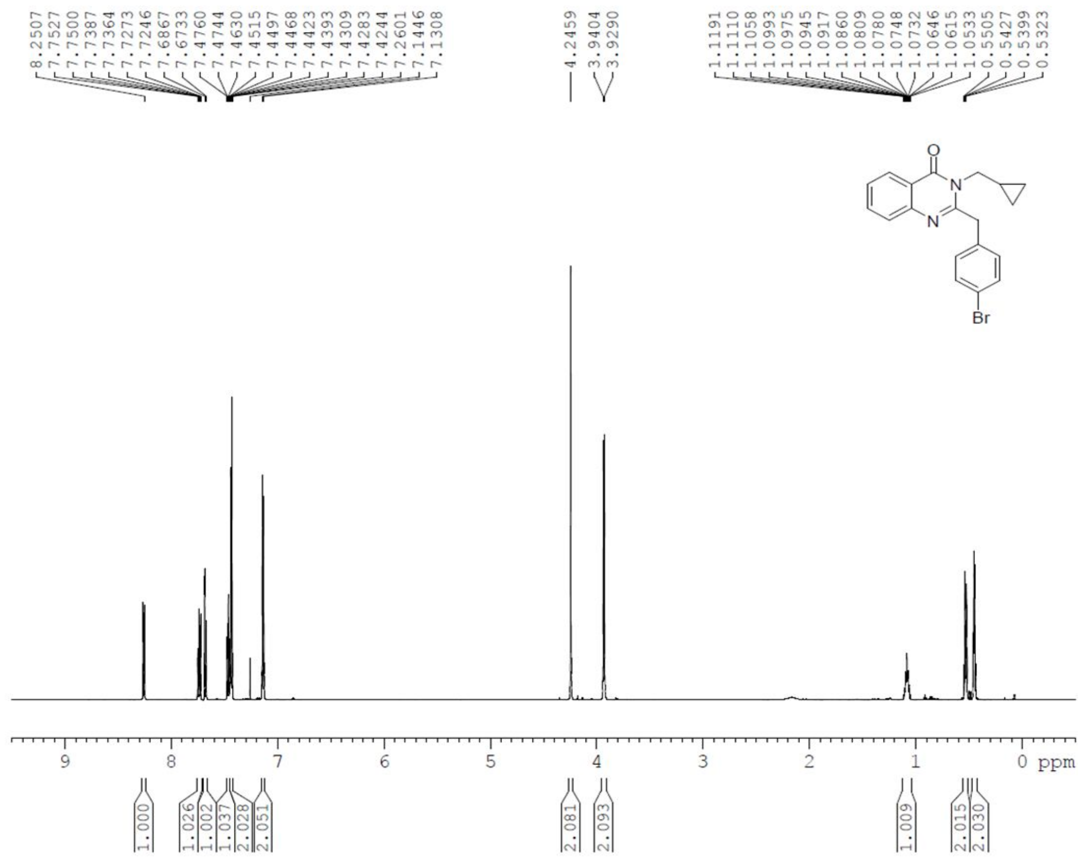

^{13}C NMR spectrum of compound **4d**

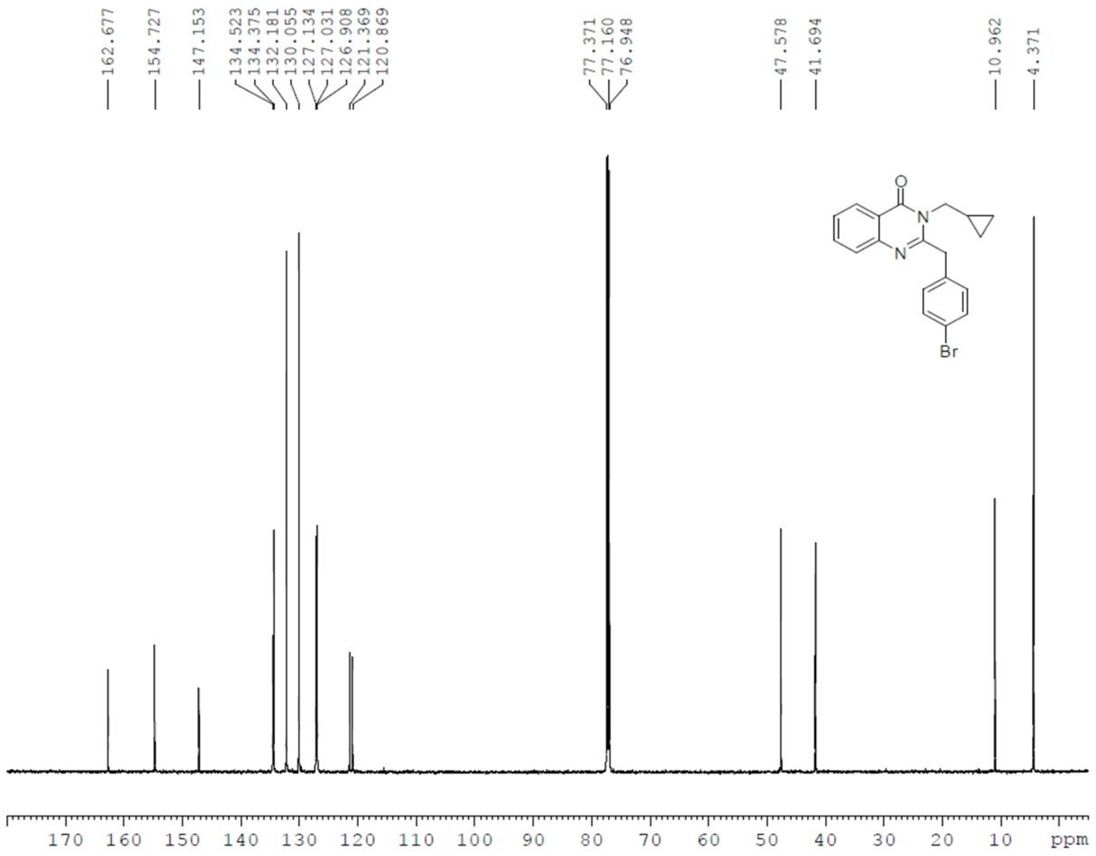

^{1}H NMR spectrum of compound **4e**

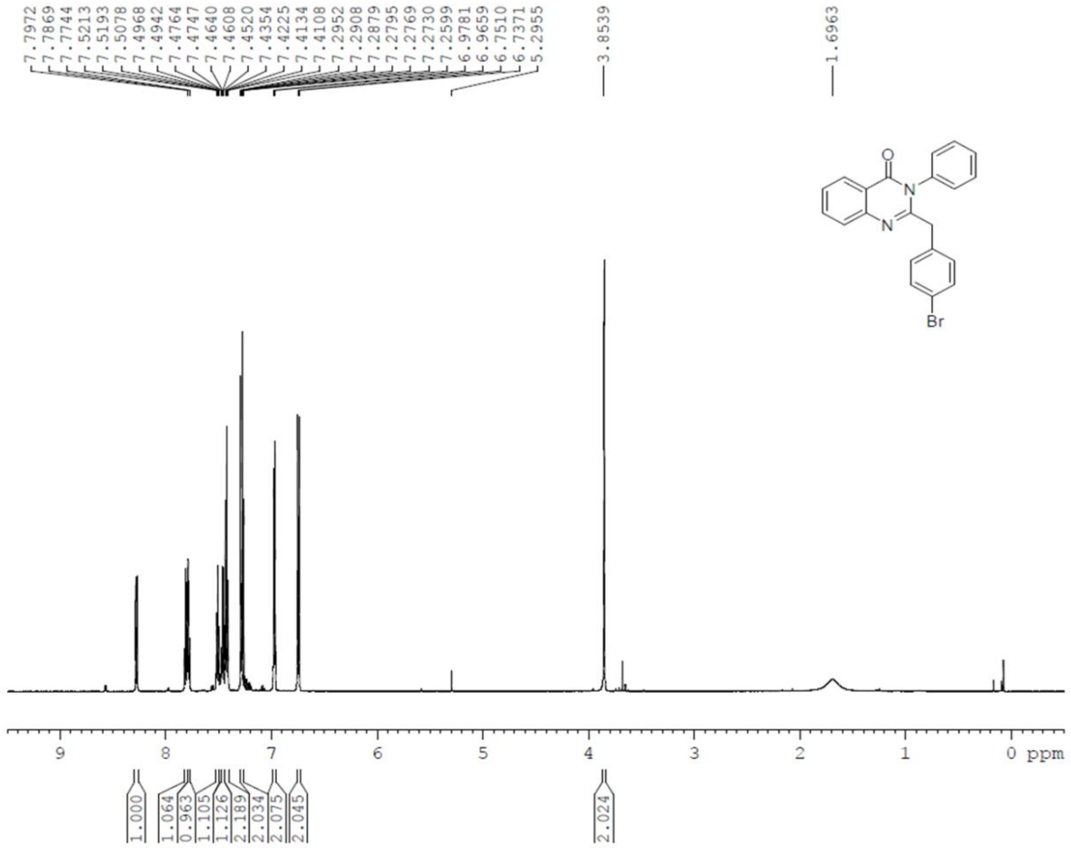

^{13}C NMR spectrum of compound **4e**

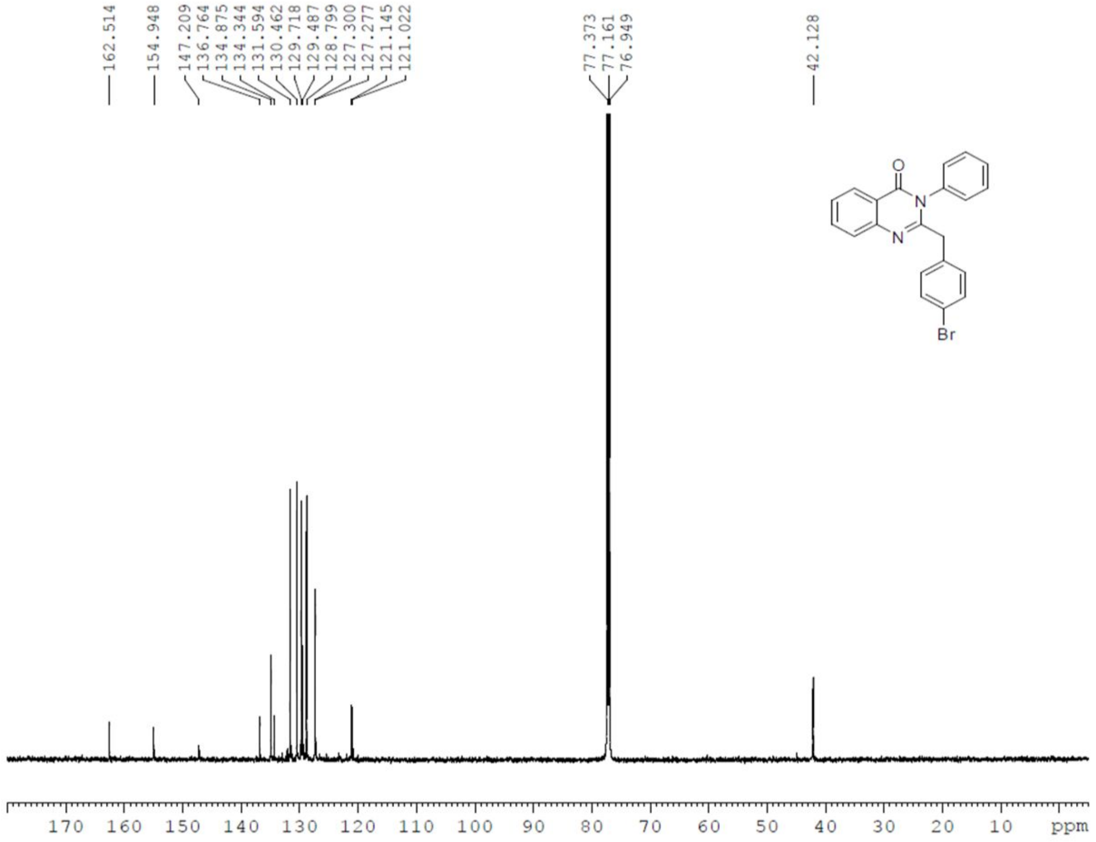

^{1}H NMR spectrum of compound **4f**

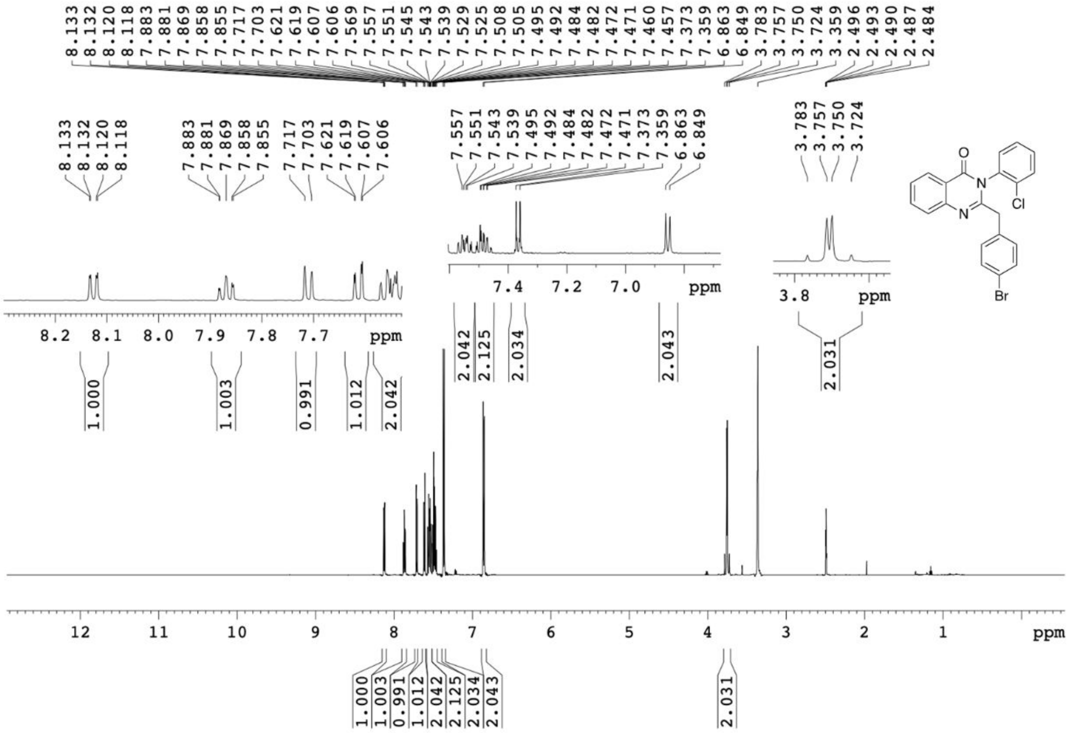

^{13}C NMR spectrum of compound **4f**

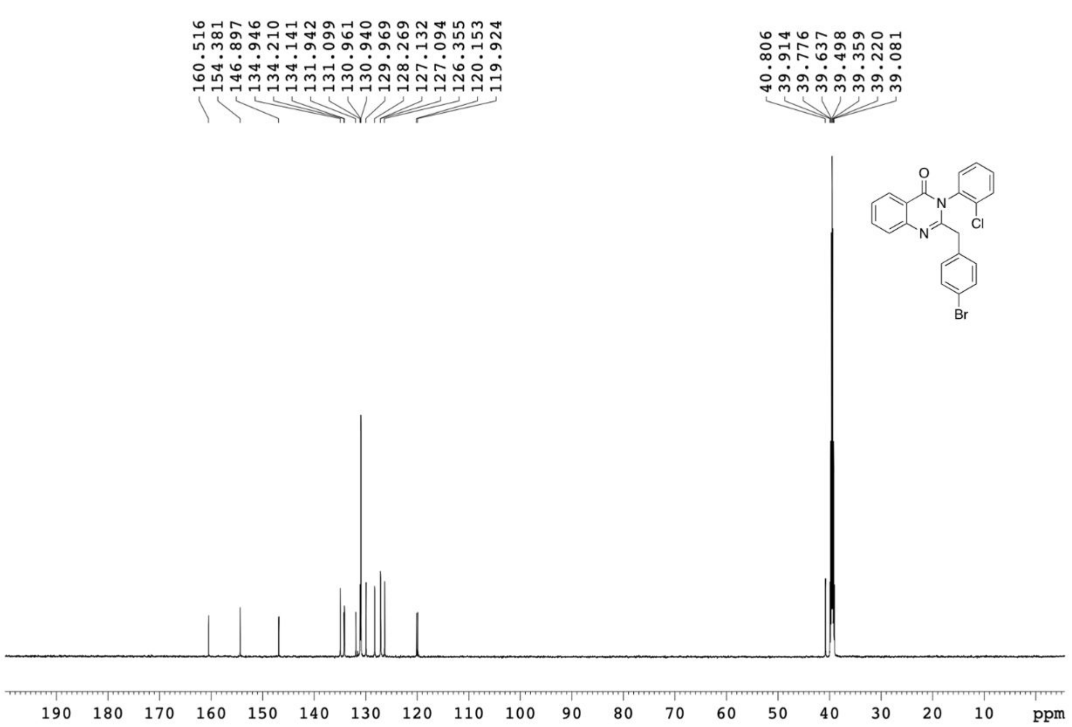

^{1}H NMR spectrum of compound **4g**

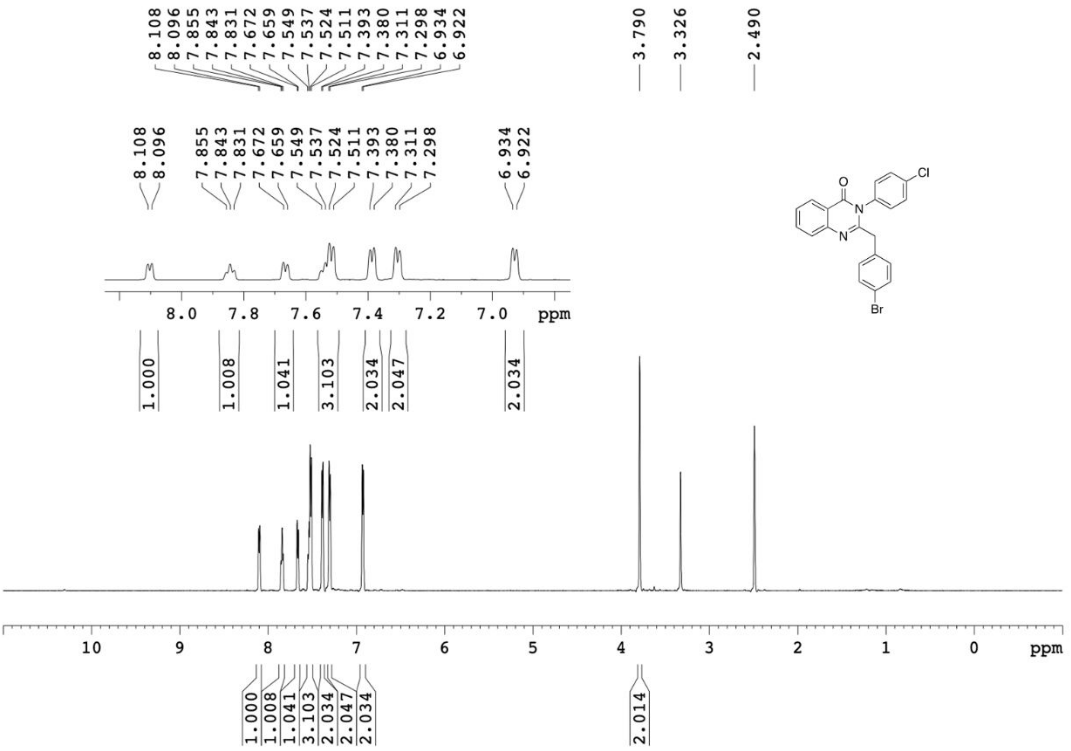

^{13}C NMR spectrum of compound **4g**

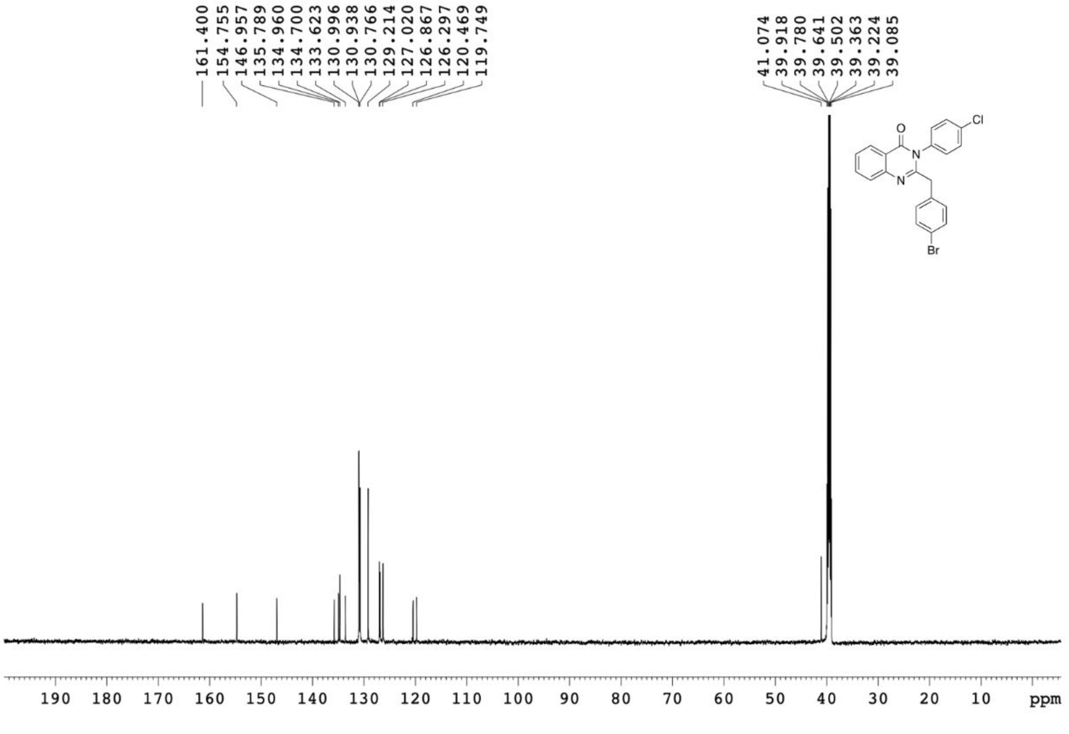

^{1}H NMR spectrum of compound **4h**

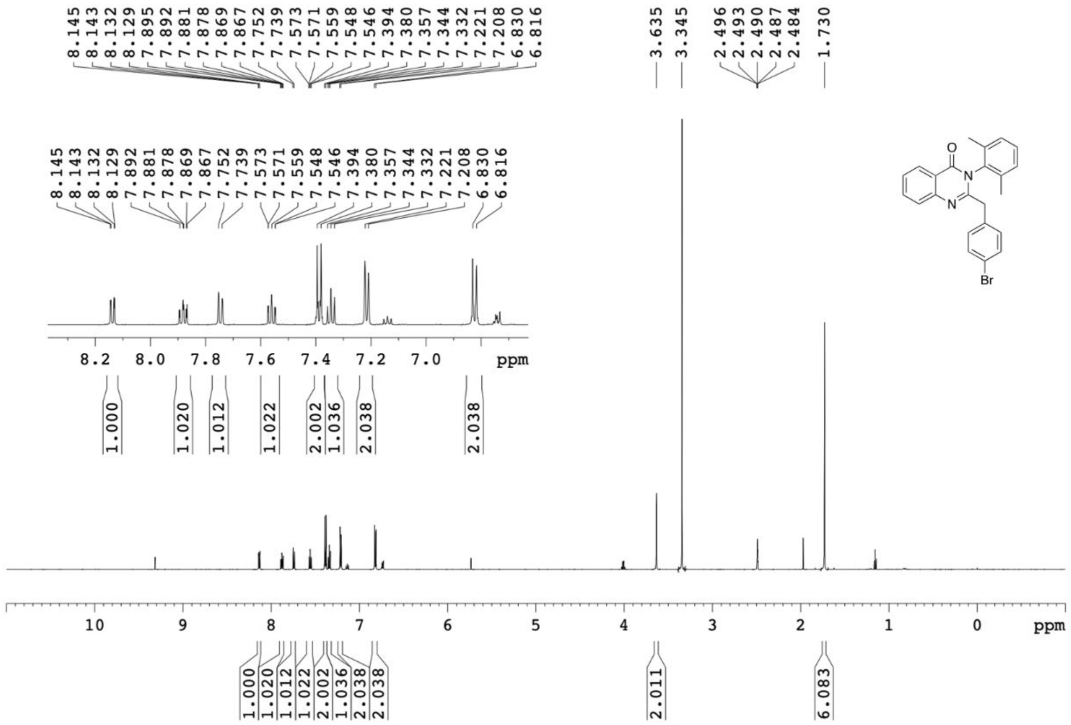

^{13}C NMR spectrum of compound **4h**

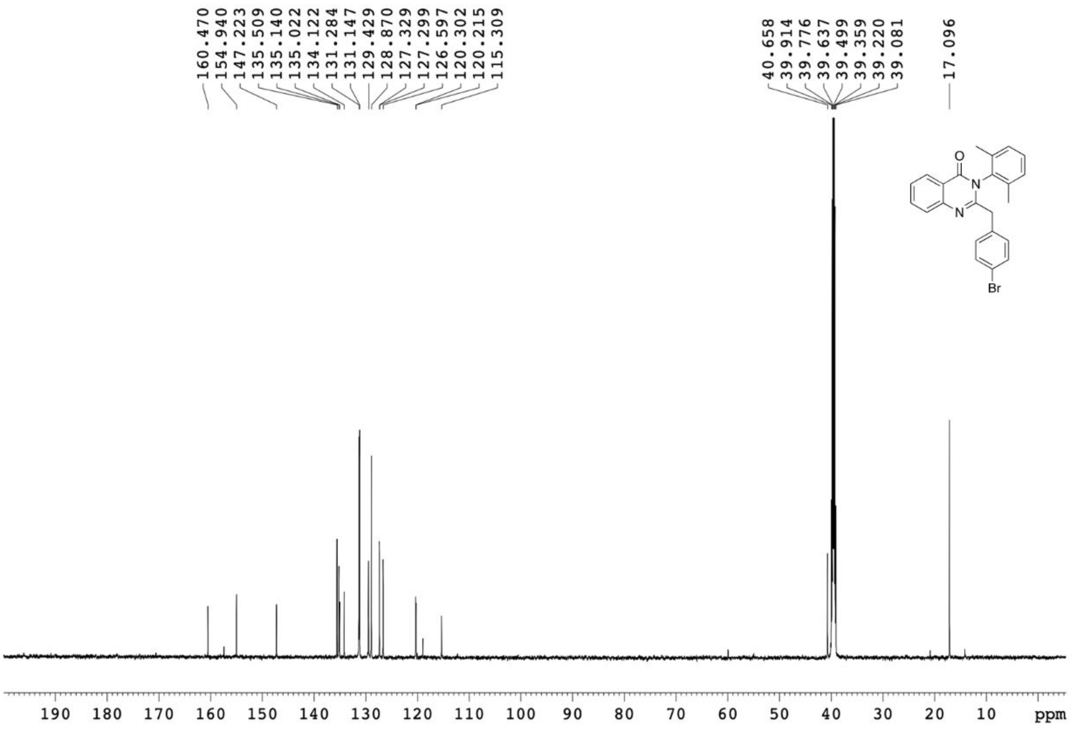

^{1}H NMR spectrum of compound **4i**

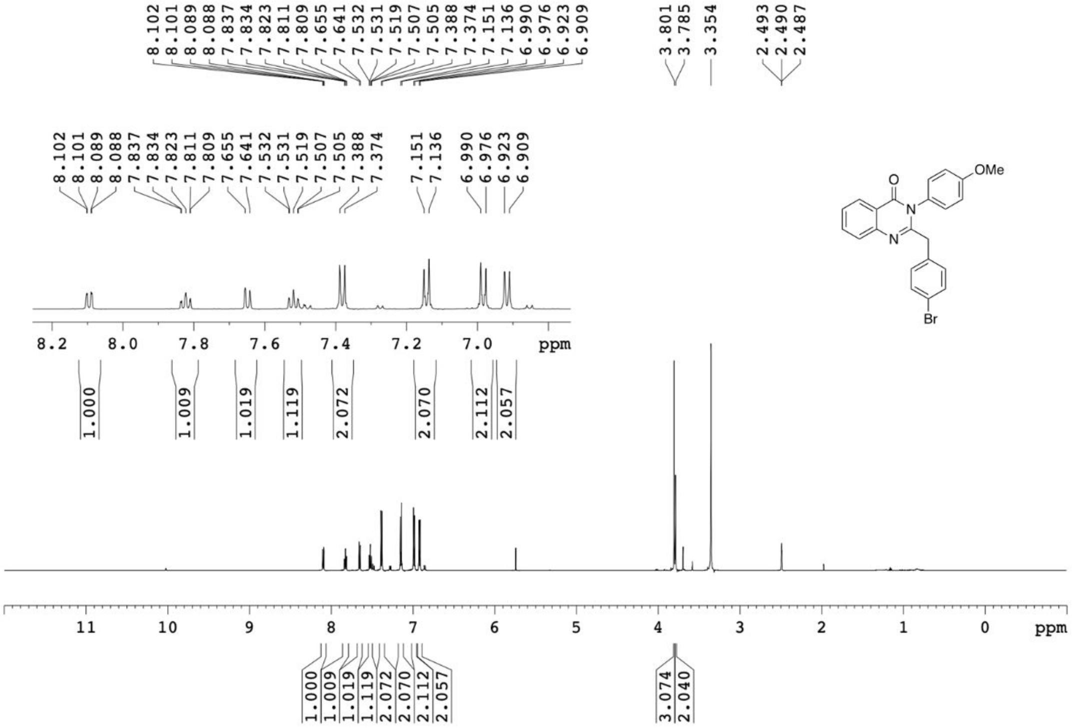

^{13}C NMR spectrum of compound **4i**

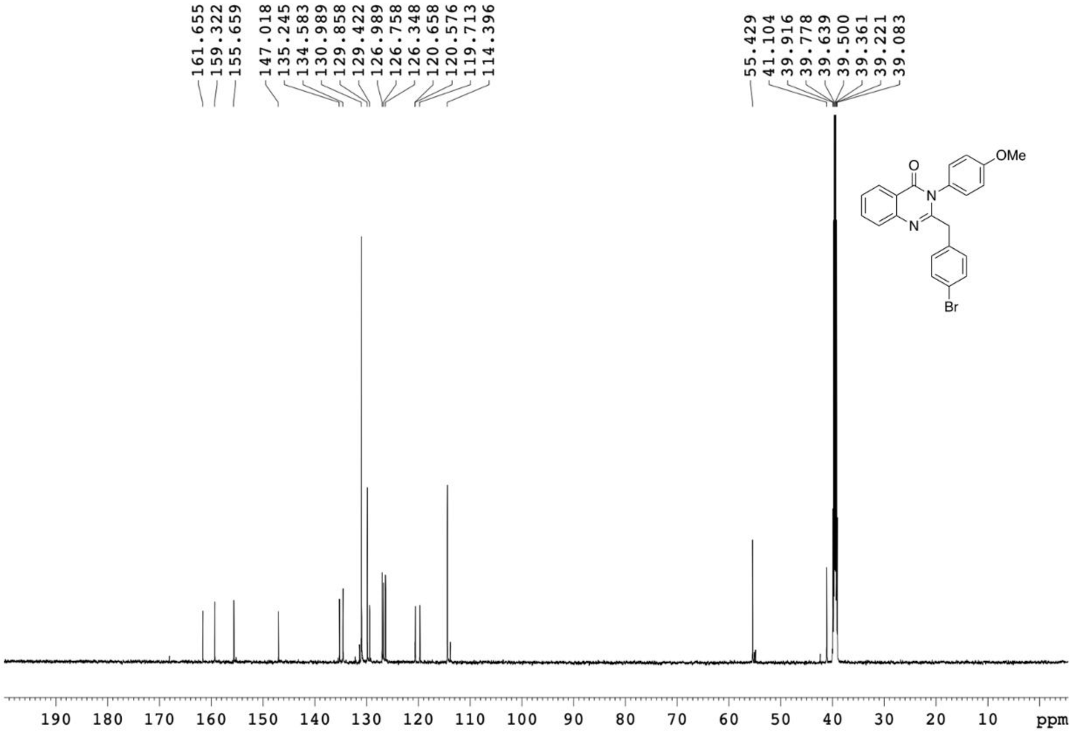

^{1}H NMR spectrum of compound **4j**

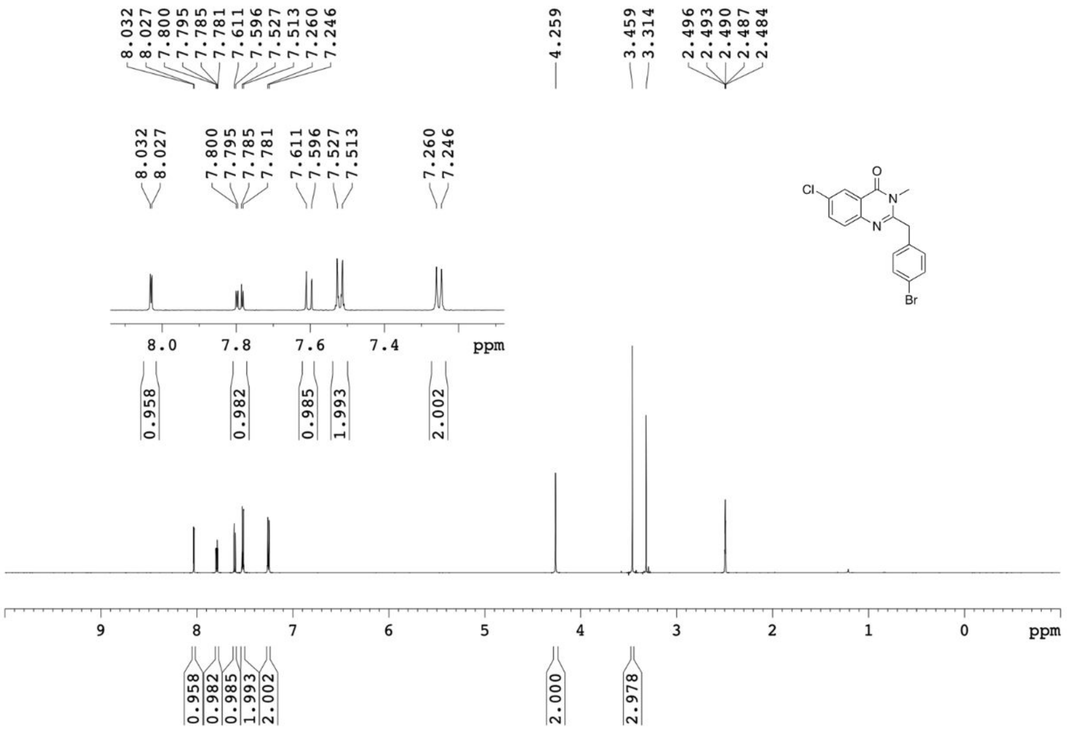

^{13}C NMR spectrum of compound **4j**

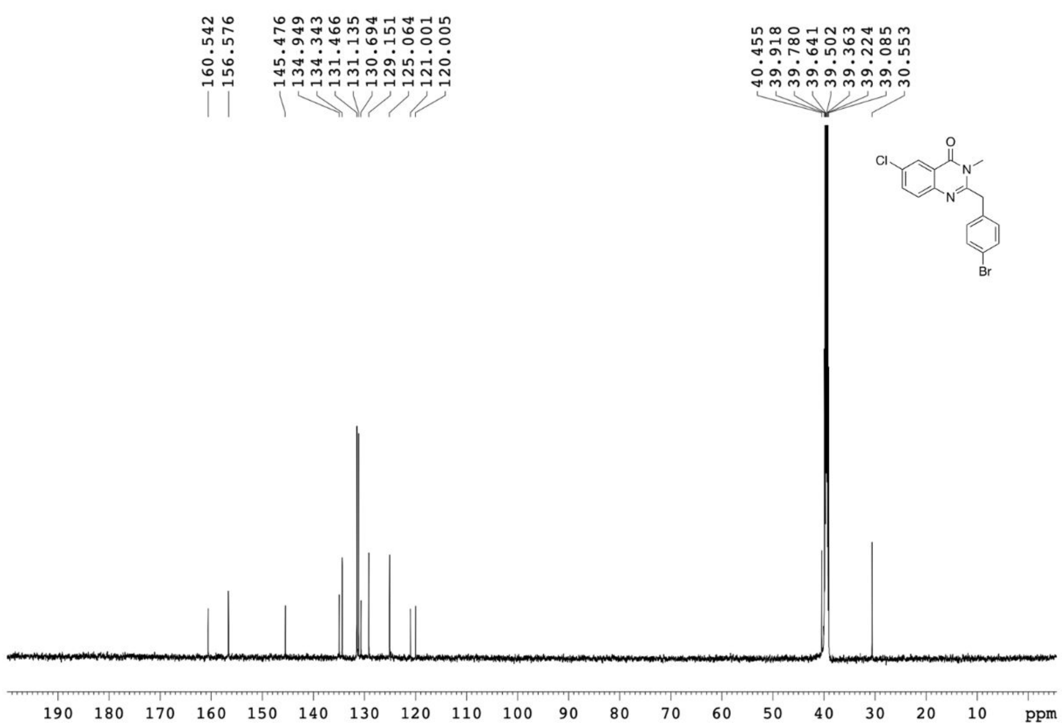

^{1}H NMR spectrum of compound **4k**

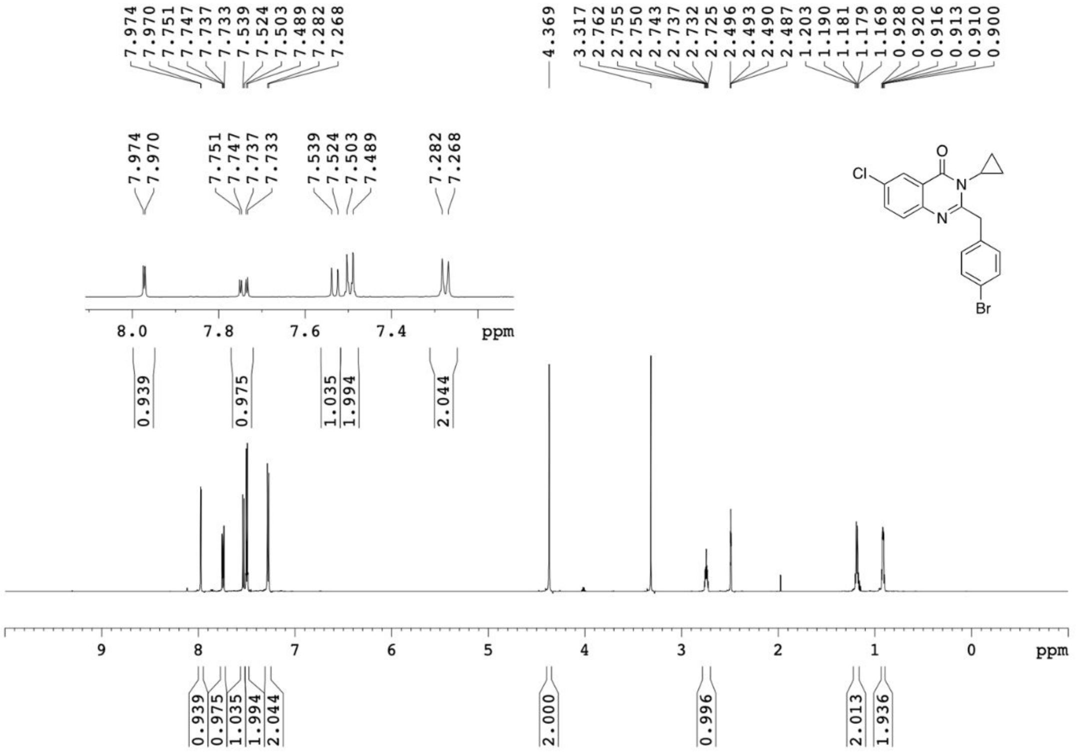

^{13}C NMR spectrum of compound **4k**

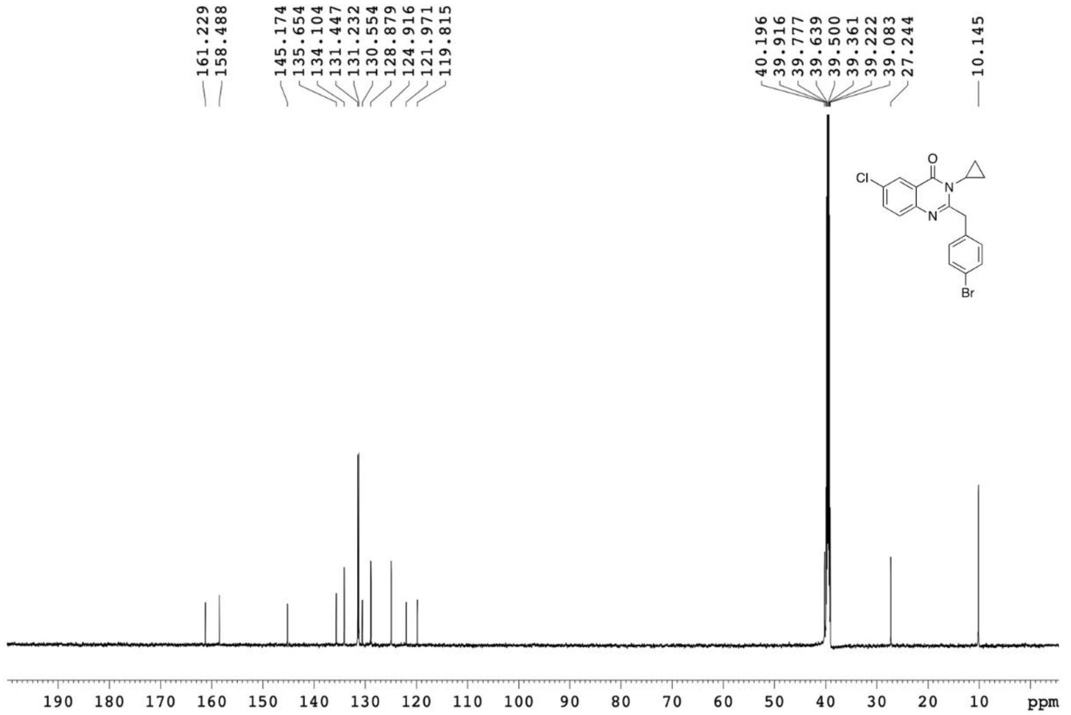

^{1}H NMR spectrum of compound **4l**

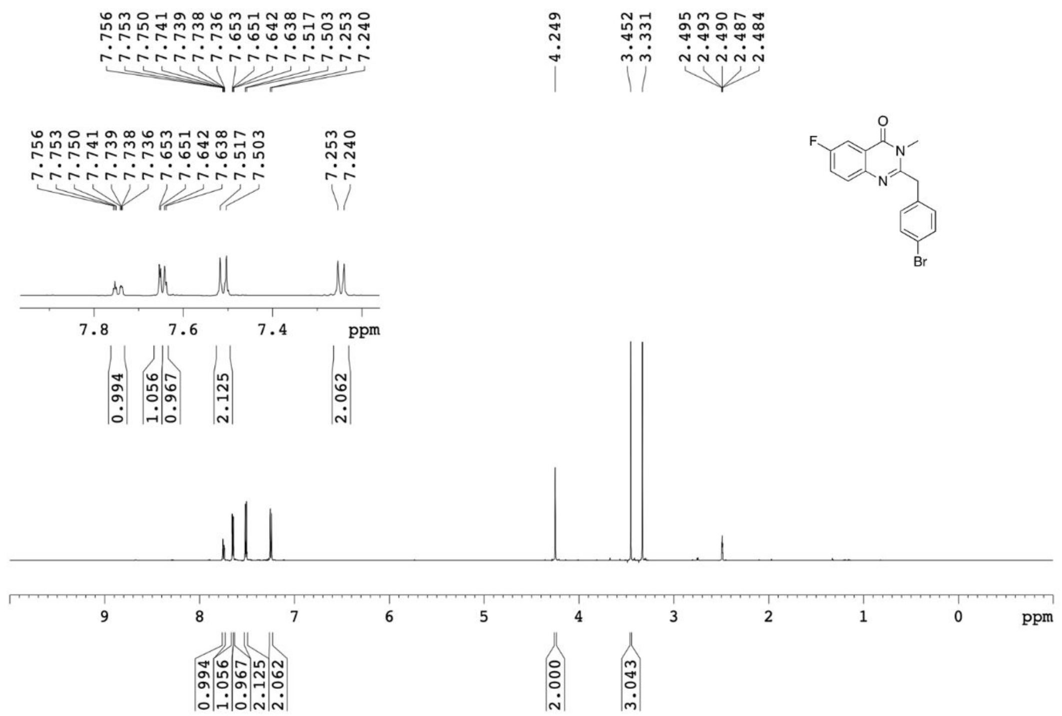

^{13}C NMR spectrum of compound **4l**

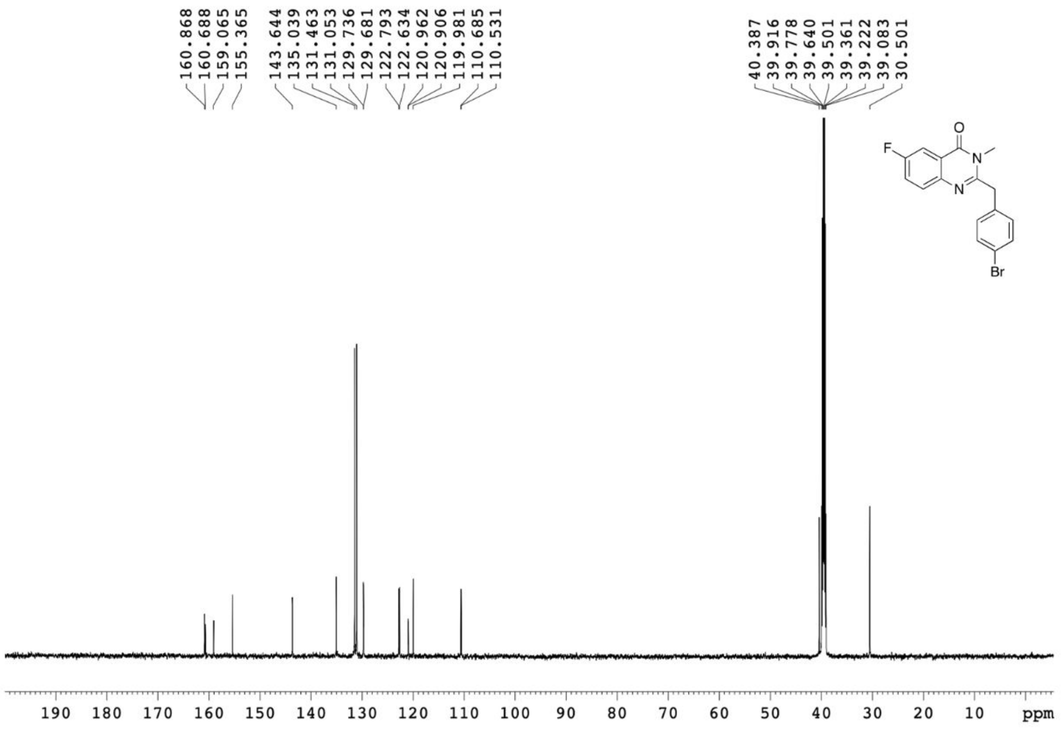

^{1}H NMR spectrum of compound **5a**

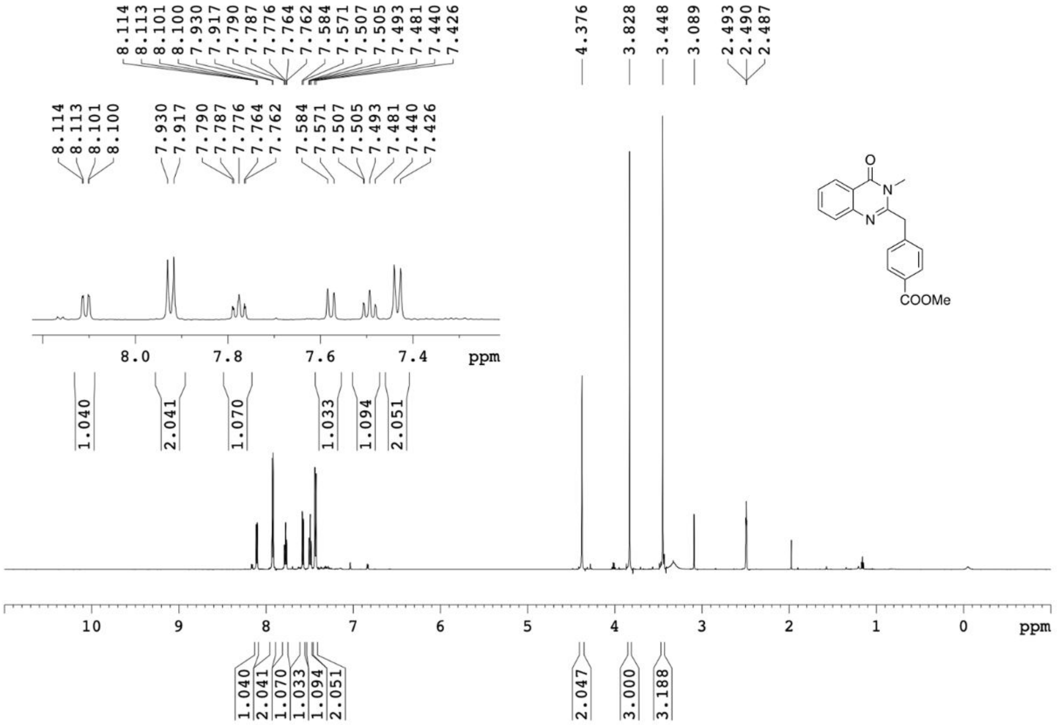

^{1}H NMR spectrum of compound **5a**

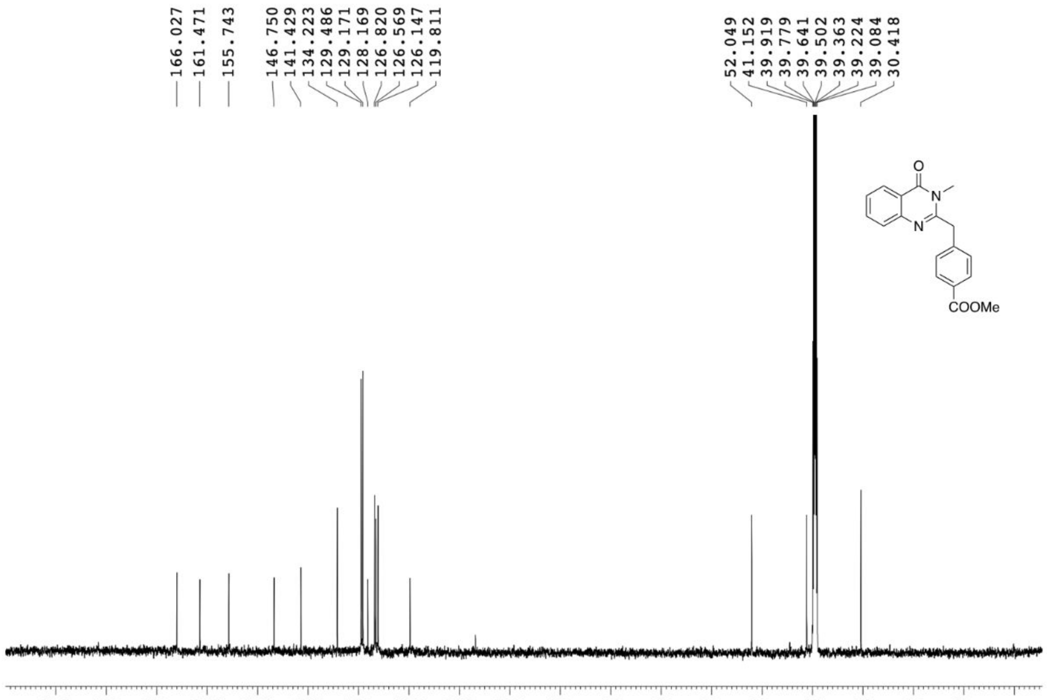

^{1}H NMR spectrum of compound **5b**

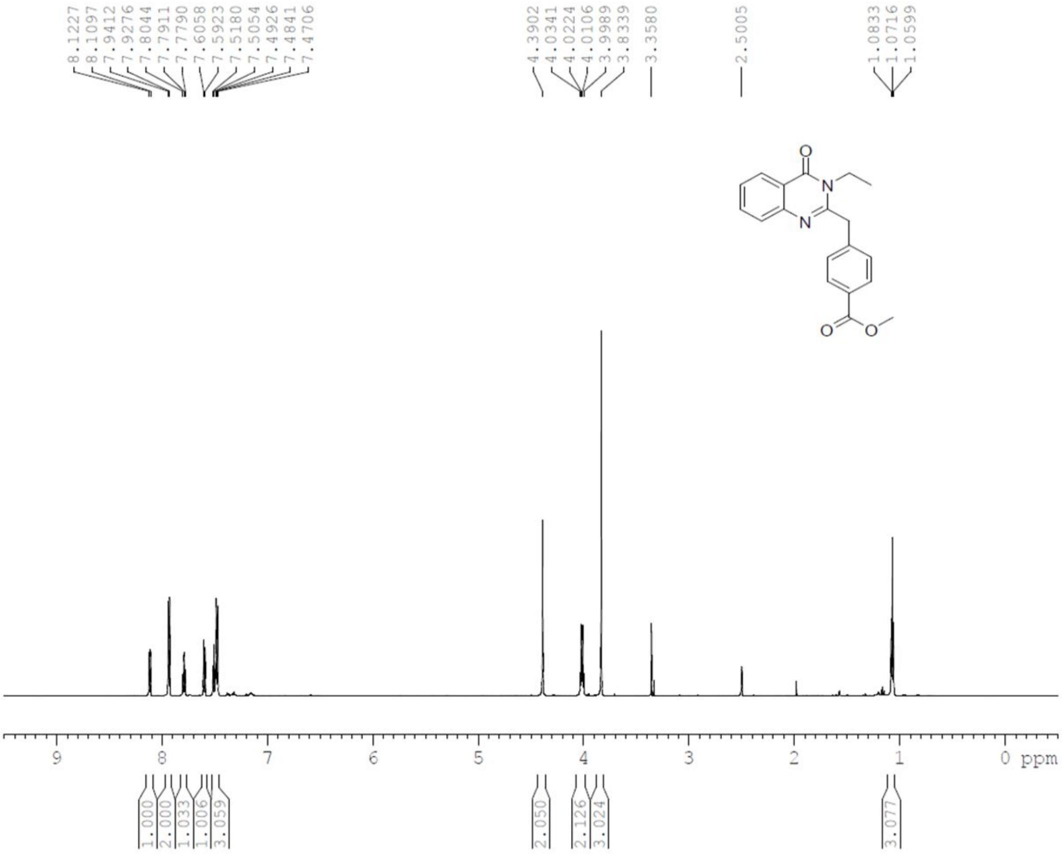

^{13}C NMR spectrum of compound **5b**

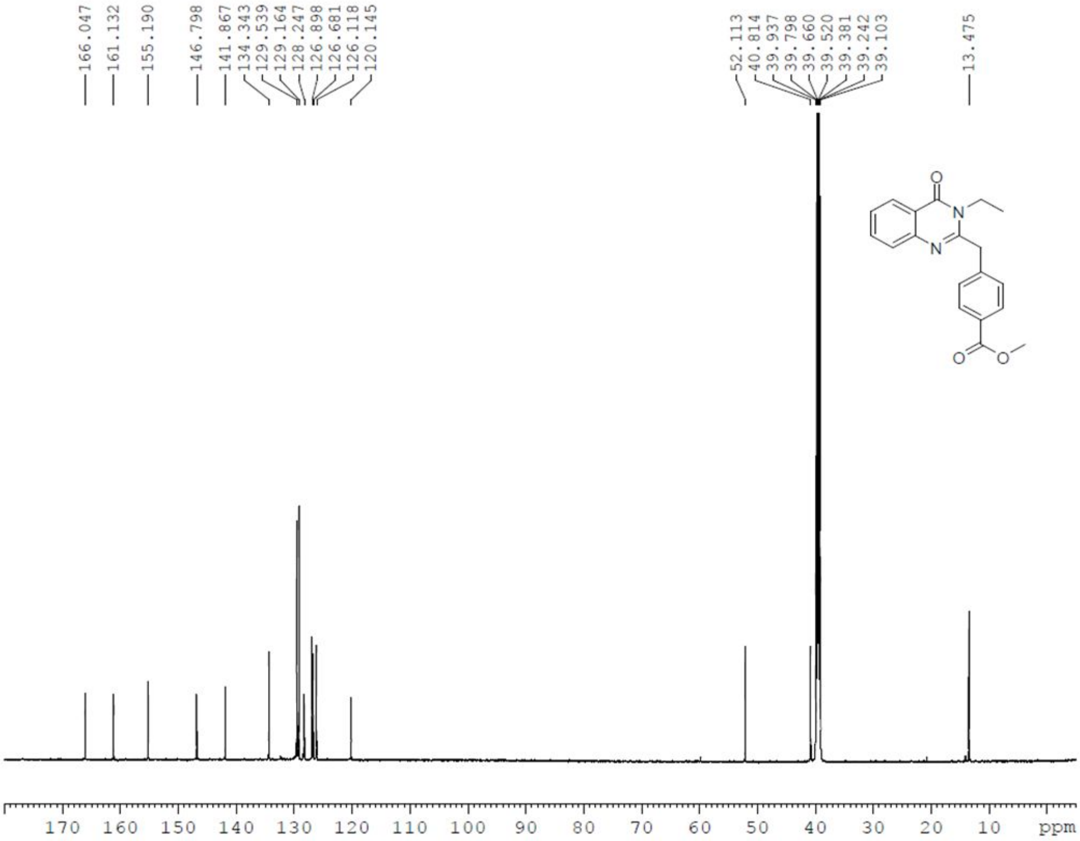

^{1}H NMR spectrum of compound **5c**

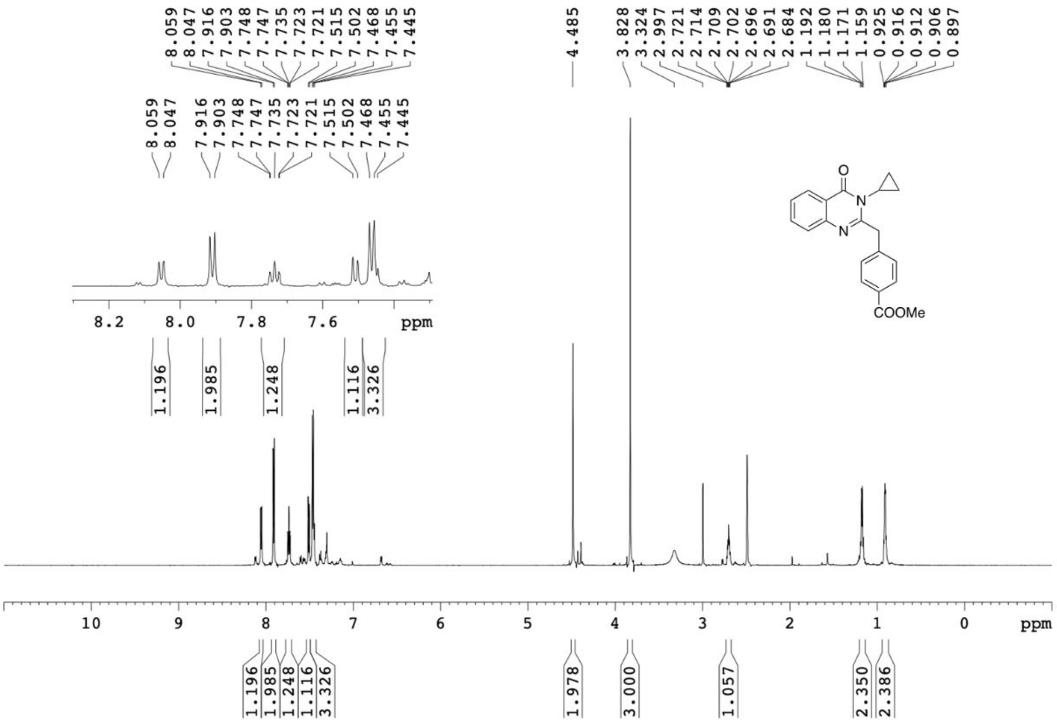

^{13}C NMR spectrum of compound **5c**

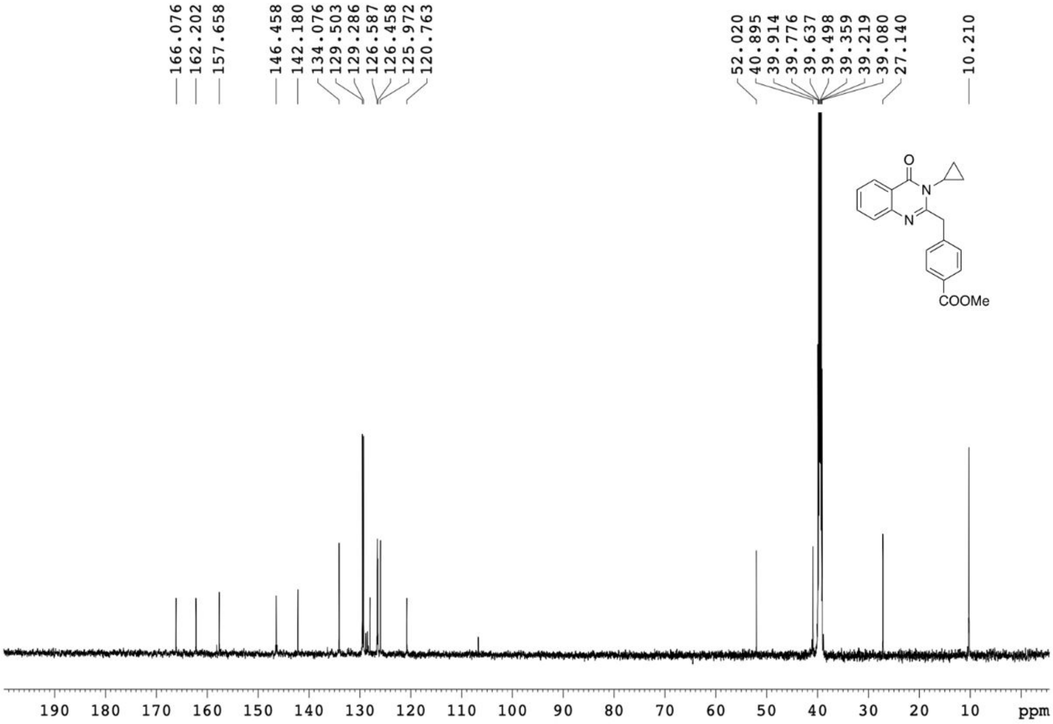

^{1}H NMR spectrum of compound **5d**

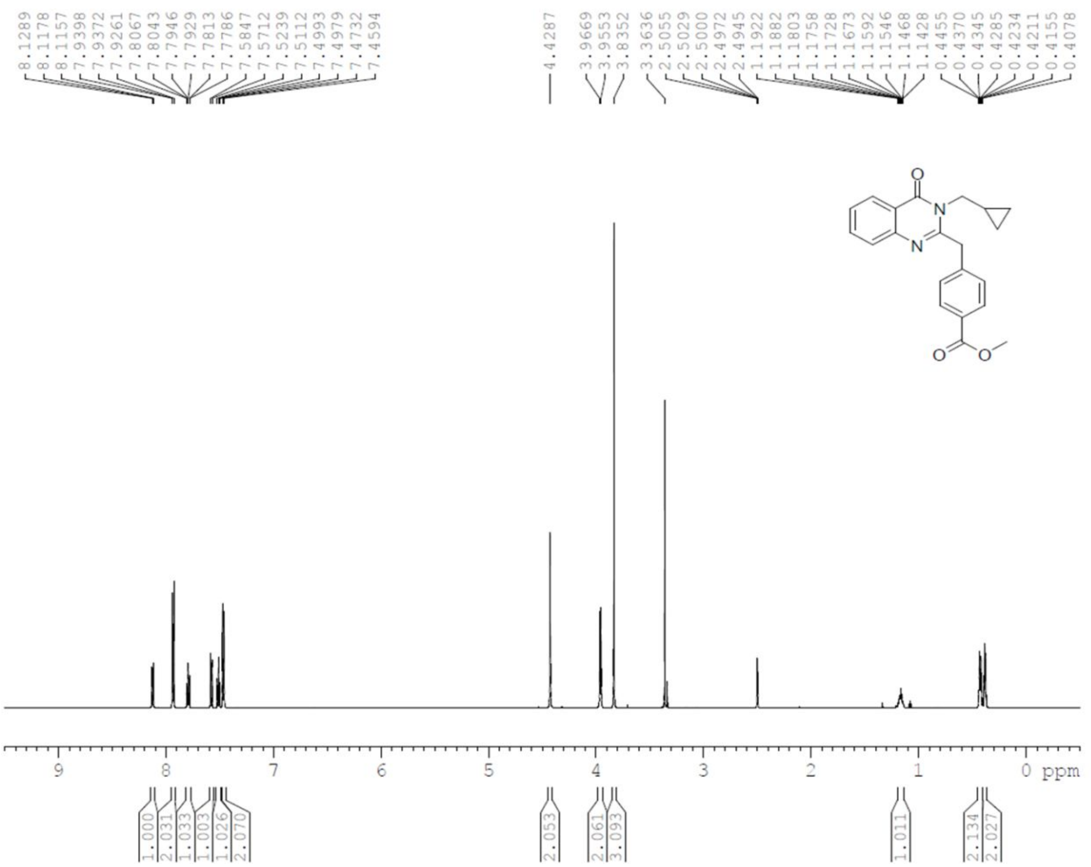

^{13}C NMR spectrum of compound **5d**

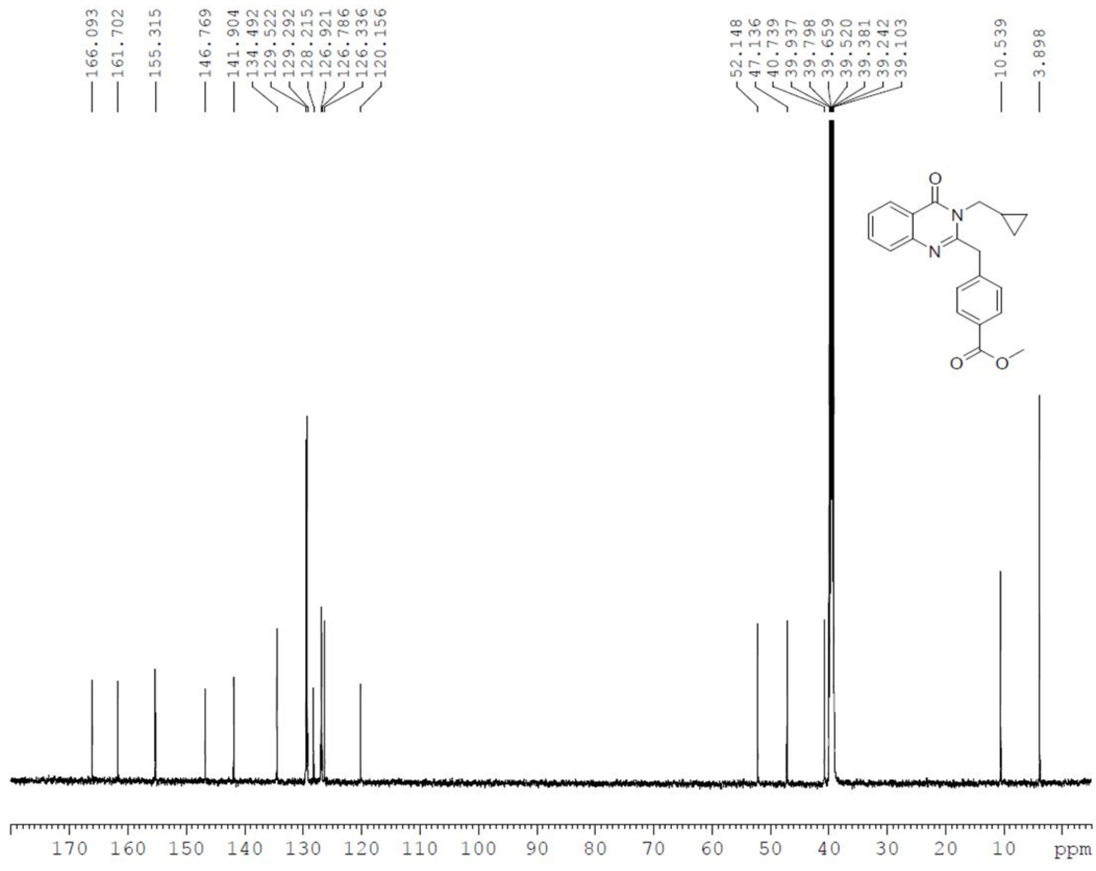

^{1}H NMR spectrum of compound **5e**

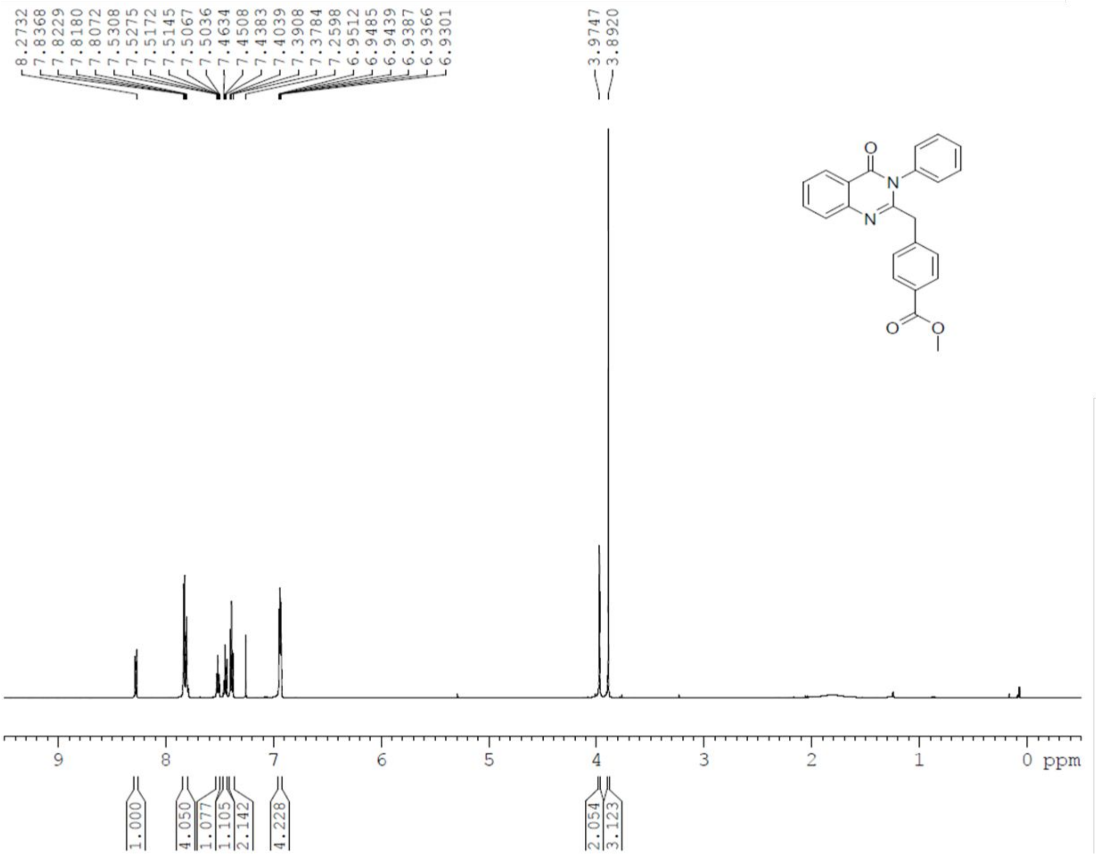

^{13}C NMR spectrum of compound **5e**

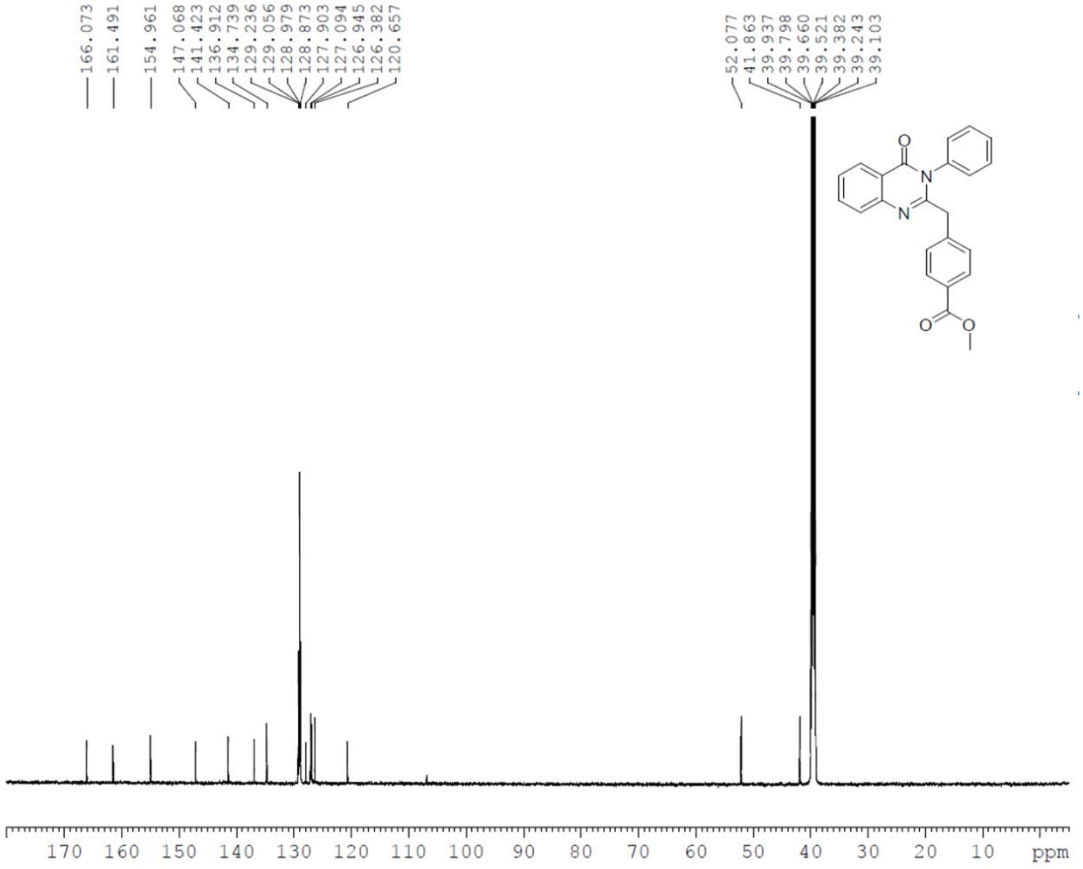

^{1}H NMR spectrum of compound **5f**

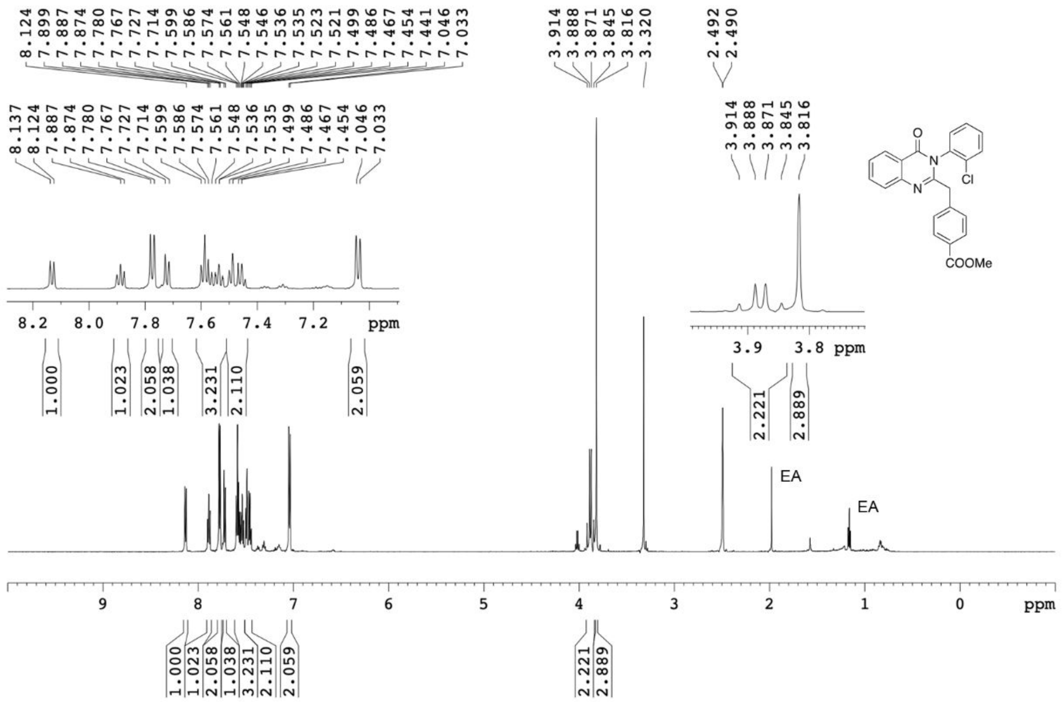

^{13}C NMR spectrum of compound **5f**

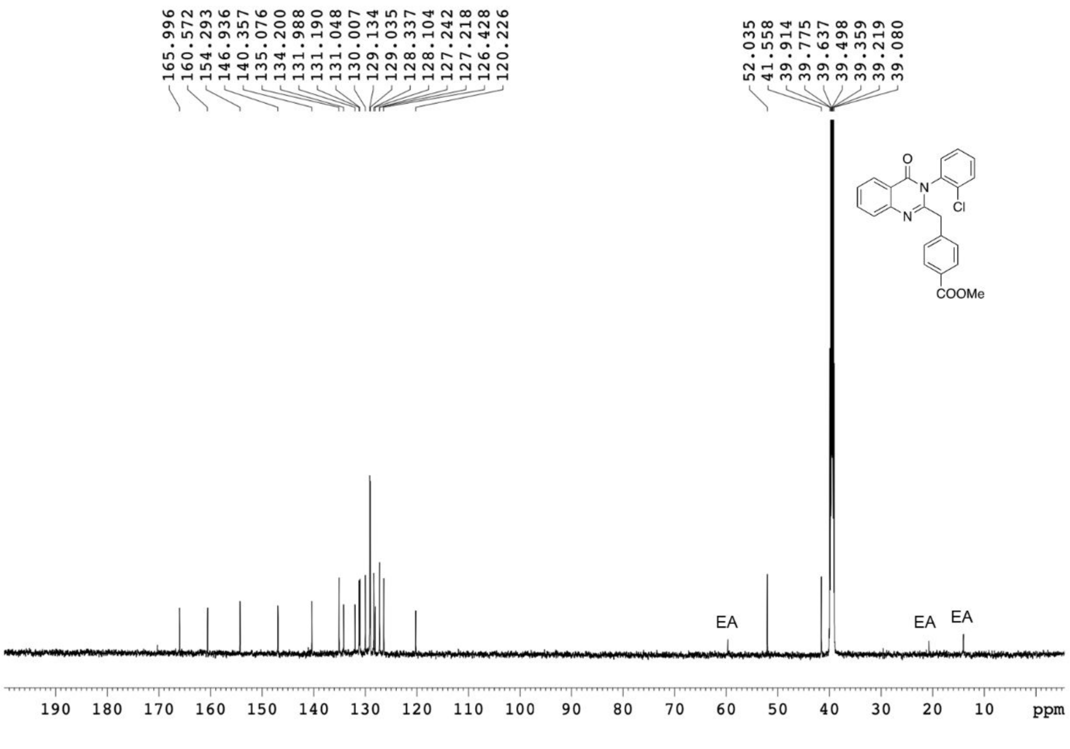

^{1}H NMR spectrum of compound **5g**

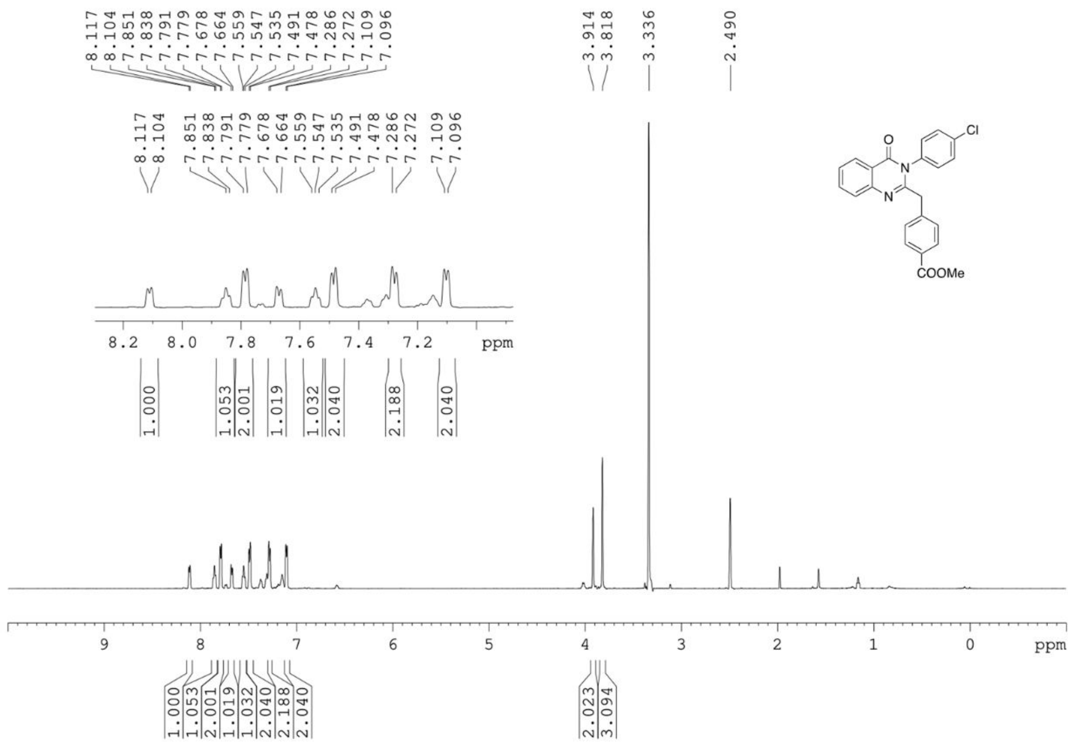

^{13}C NMR spectrum of compound **5g**

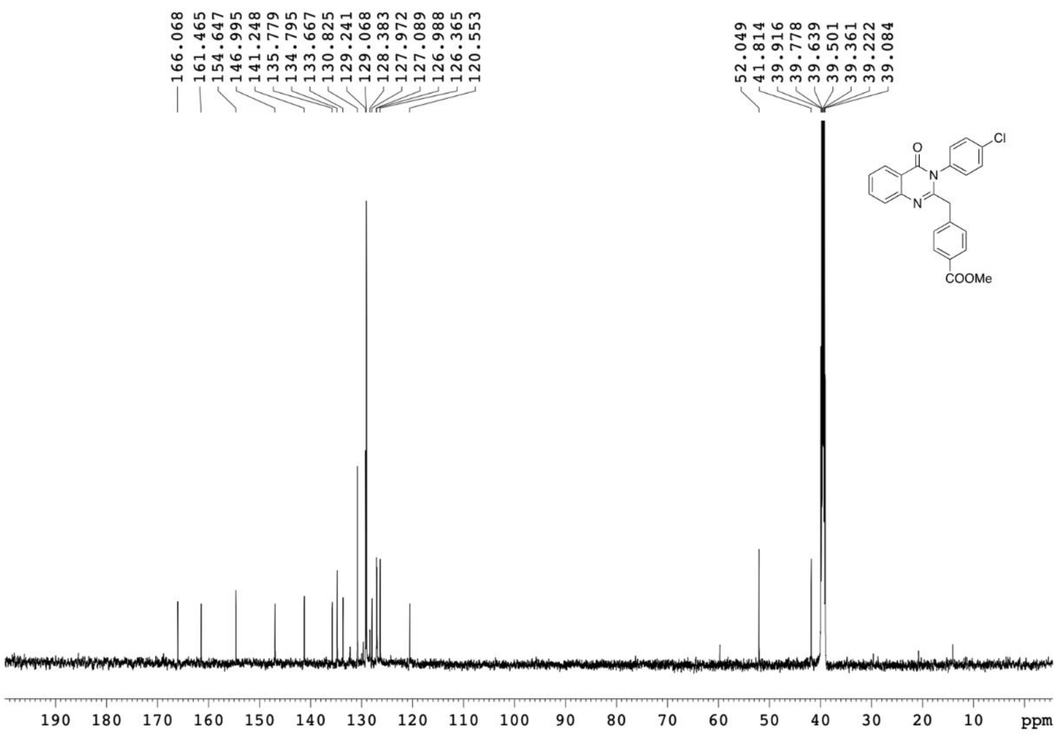

^{1}H NMR spectrum of compound **5h**

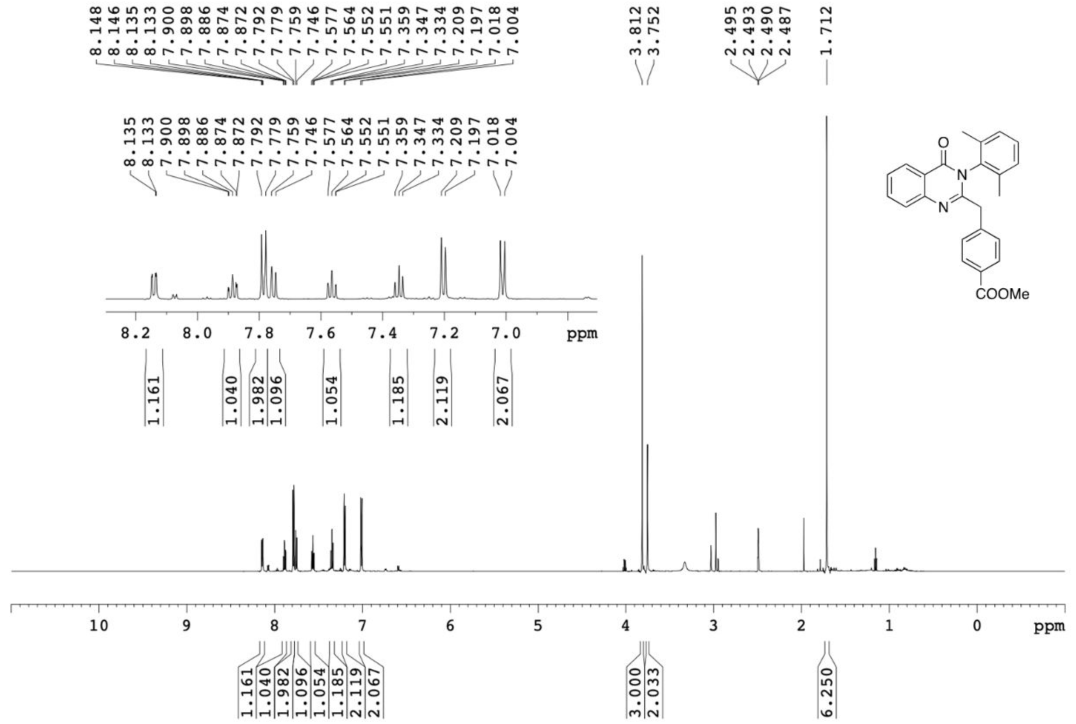

^{13}C NMR spectrum of compound **5h**

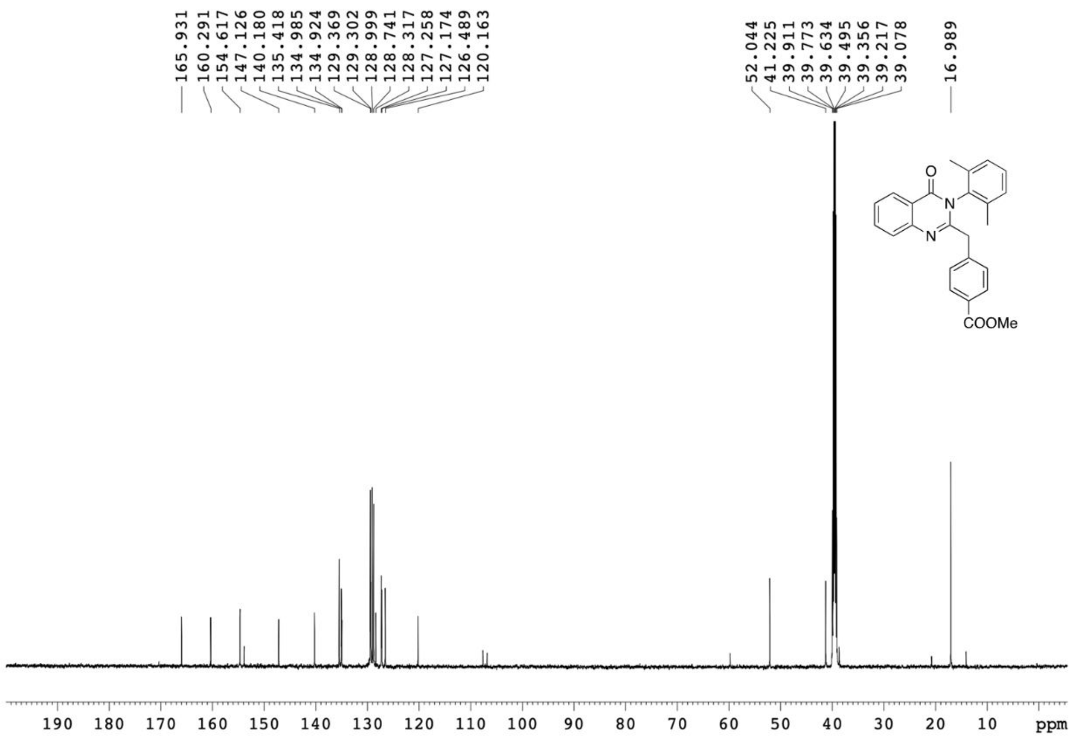

^{1}H NMR spectrum of compound **5i**

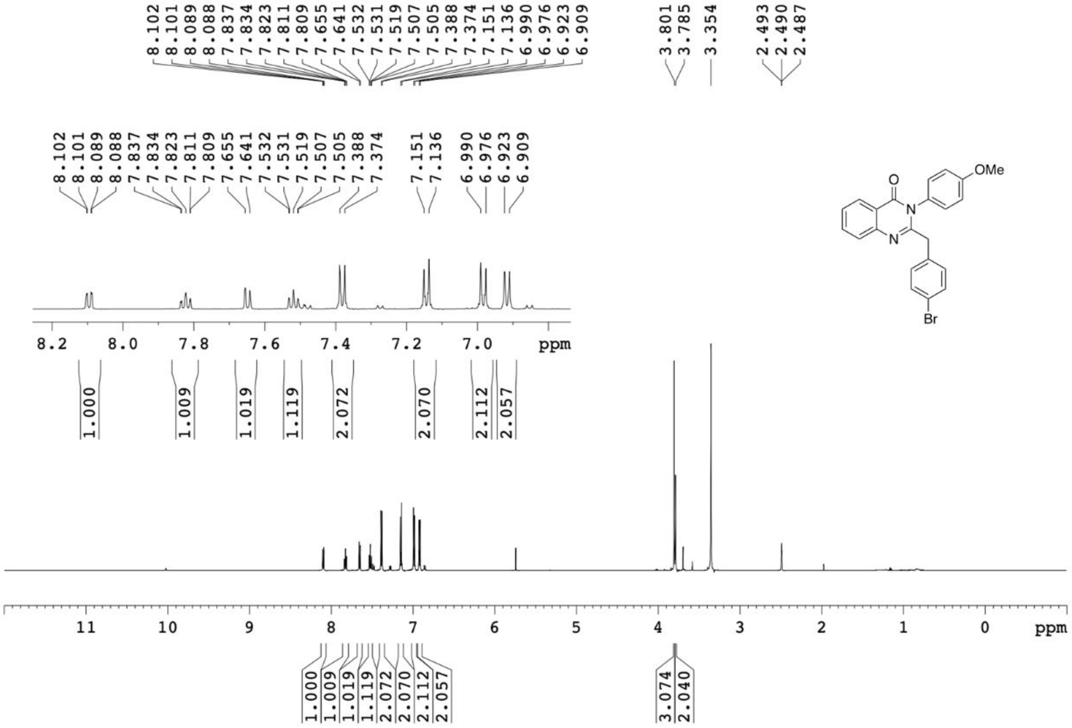

^{13}C NMR spectrum of compound **5i**

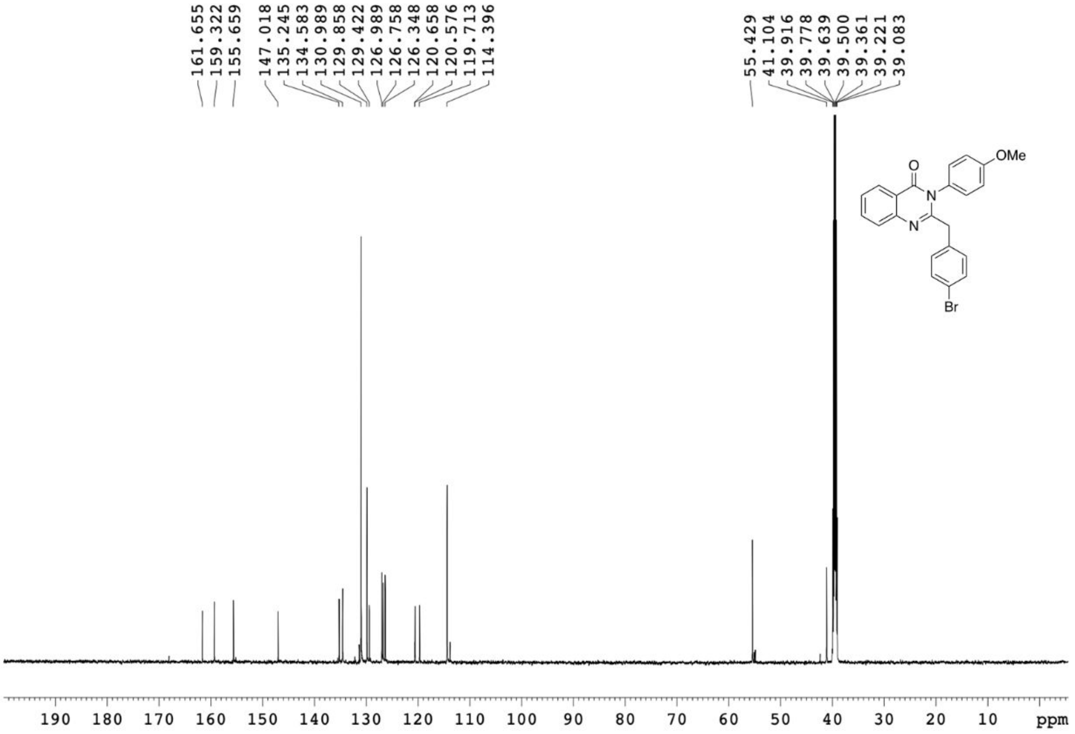

^{1}H NMR spectrum of compound **5j**

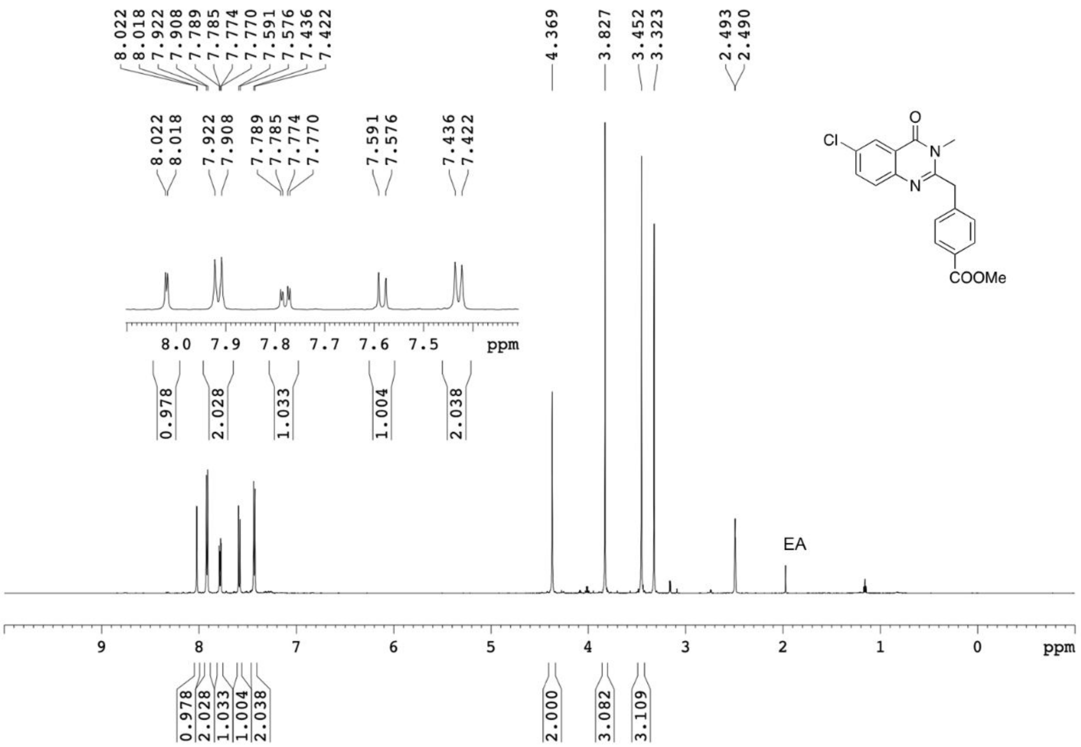

^{13}C NMR spectrum of compound **5j**

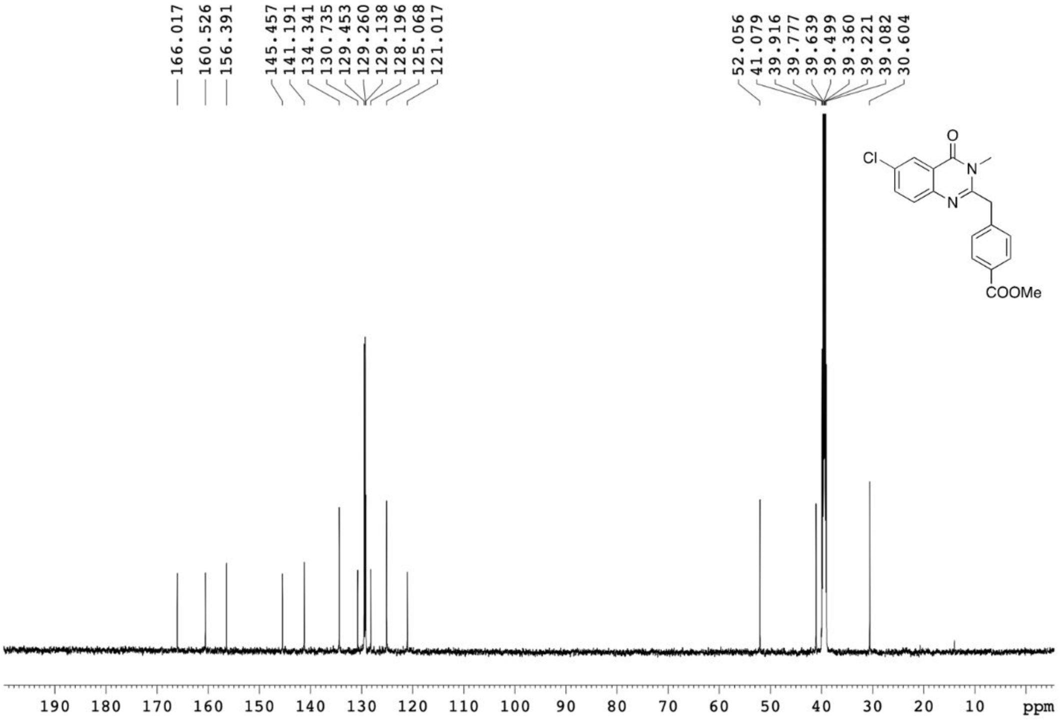

^{1}H NMR spectrum of compound **5k**

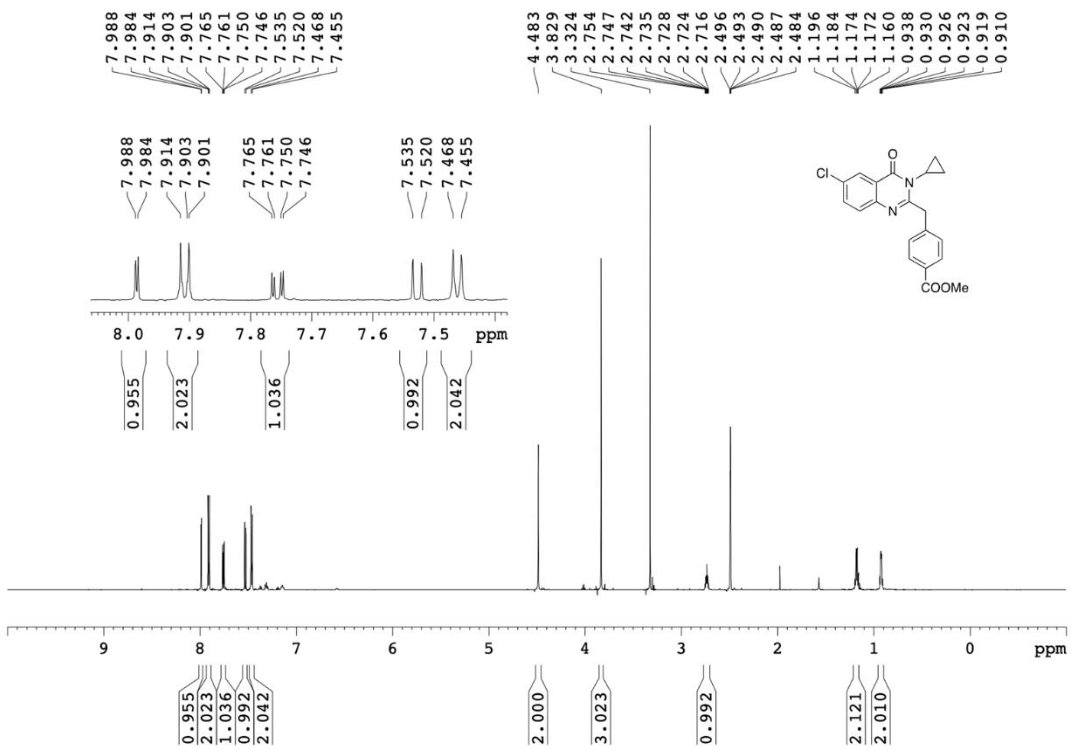

^{13}C NMR spectrum of compound **5k**

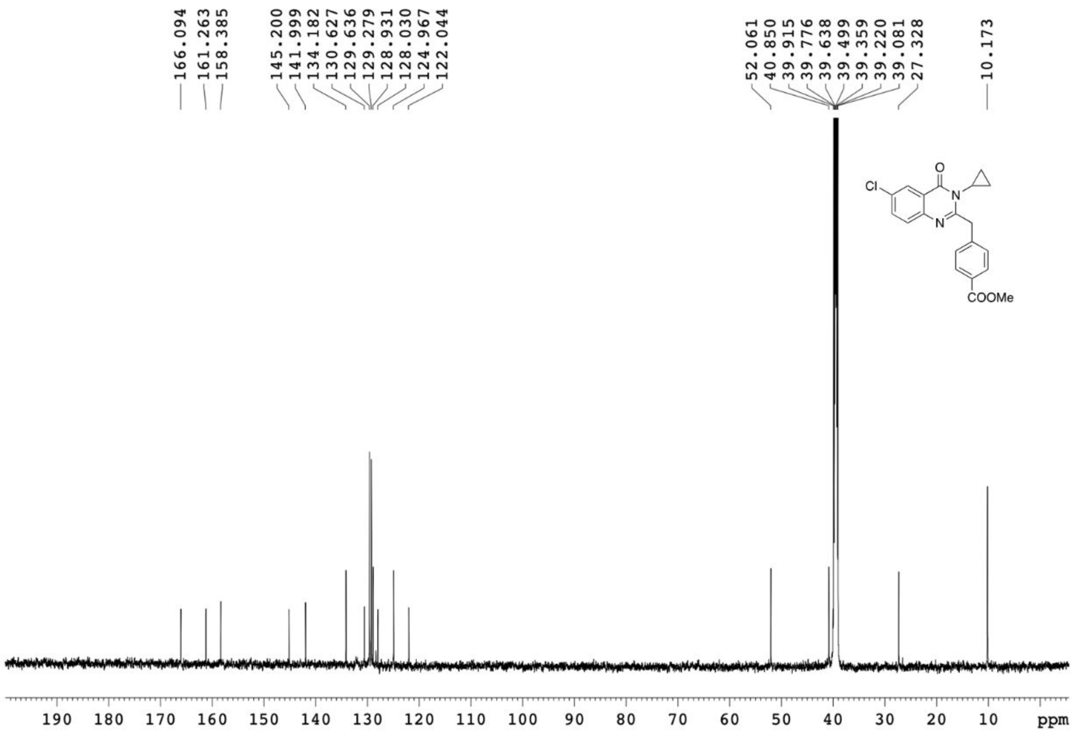

^{1}H NMR spectrum of compound **5l**

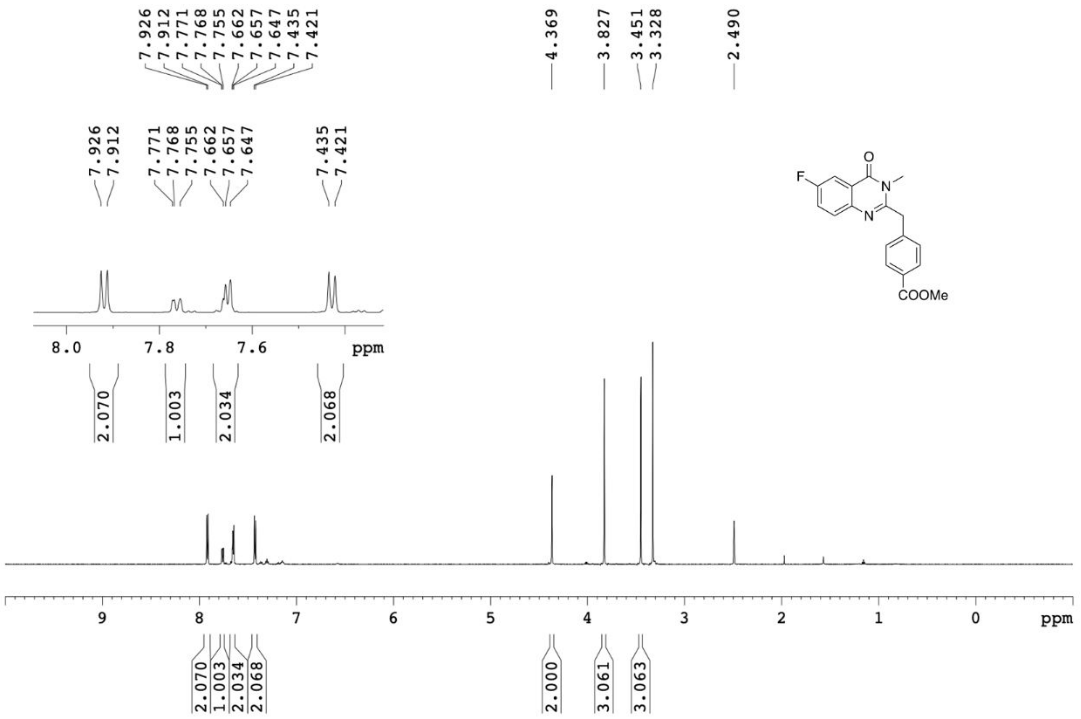

^{13}C NMR spectrum of compound **5l**

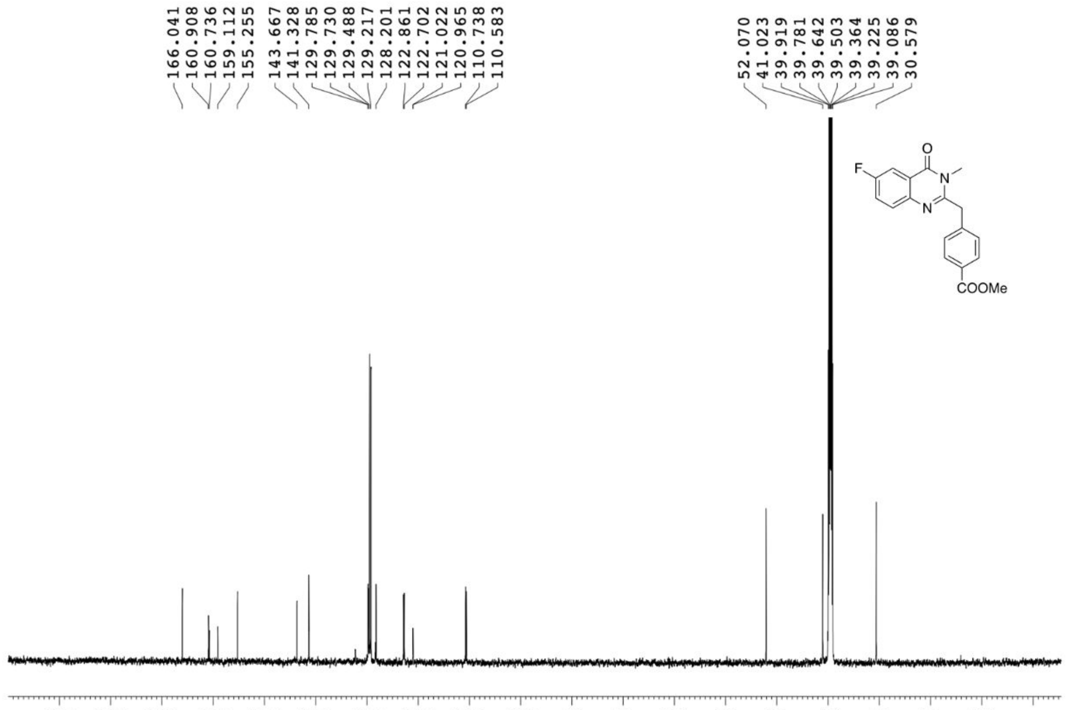

^{1}H NMR spectrum of compound **6a**

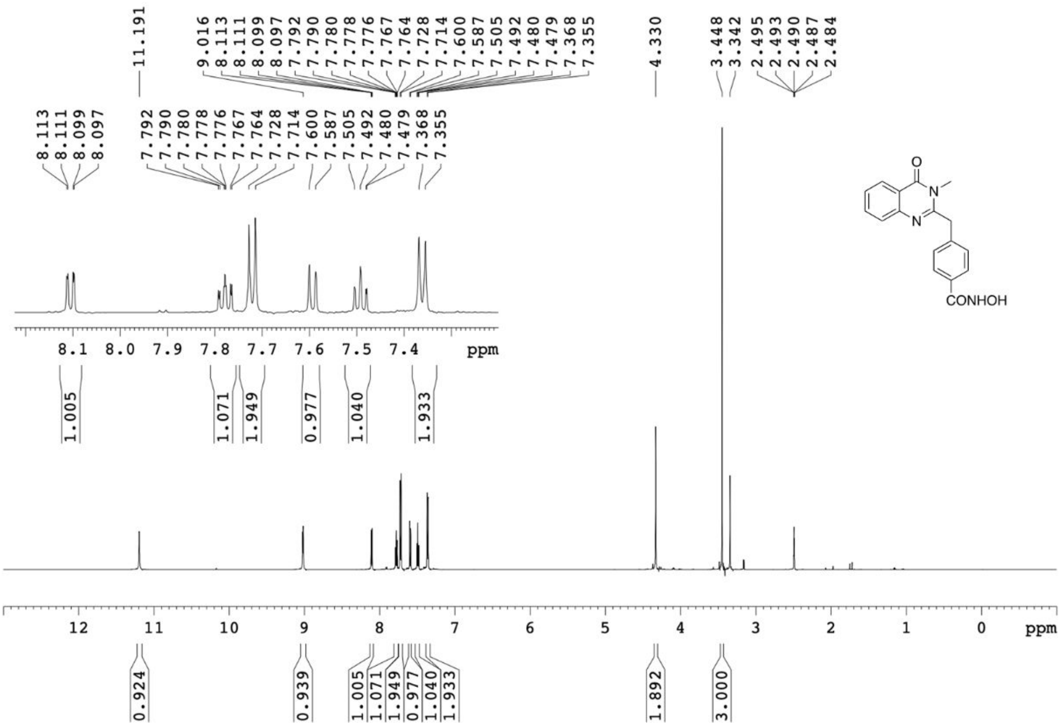

^{13}C NMR spectrum of compound **6a**

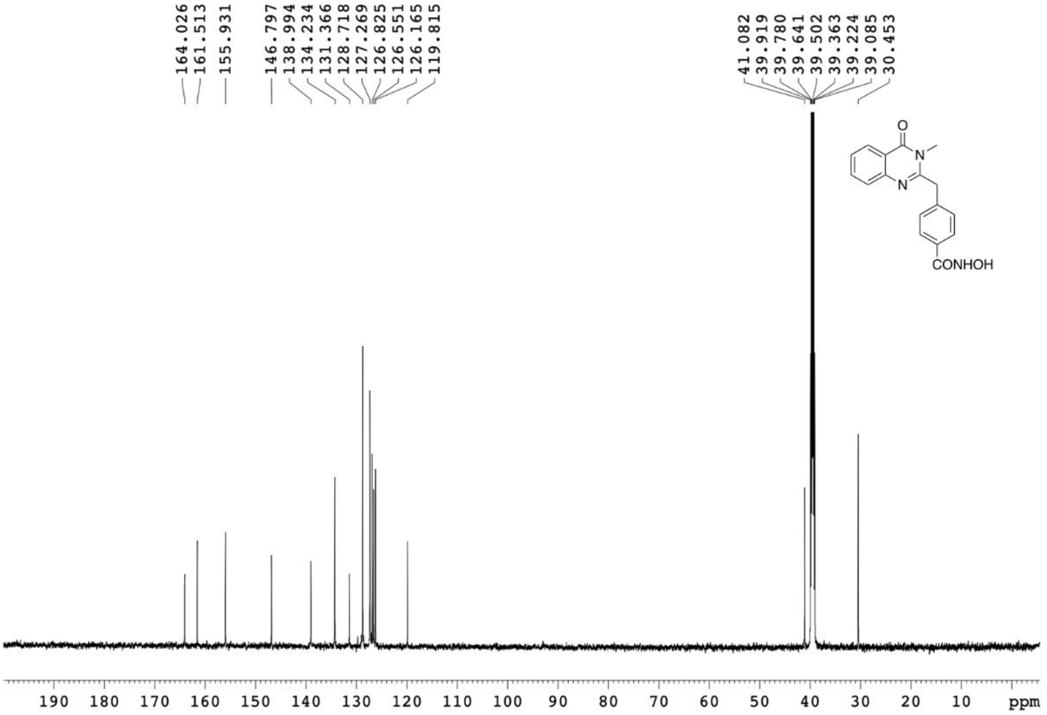

^{1}H NMR spectrum of compound **6b**

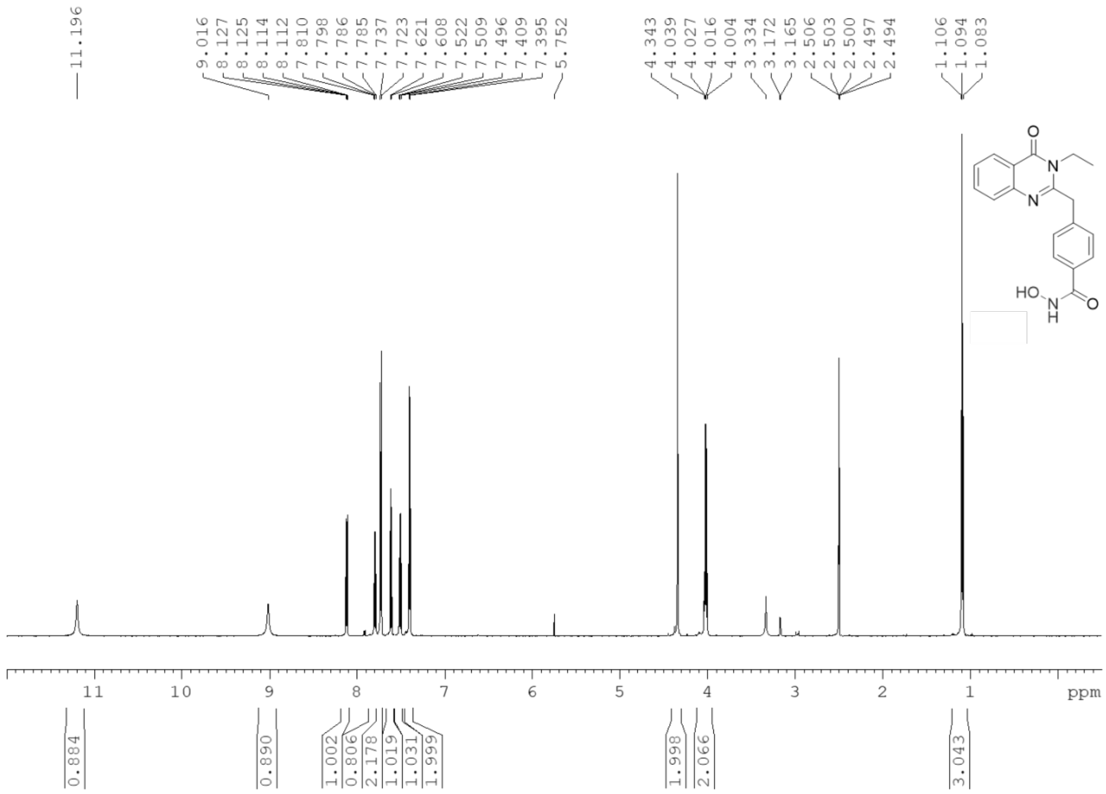

^{13}C NMR spectrum of compound **6b**

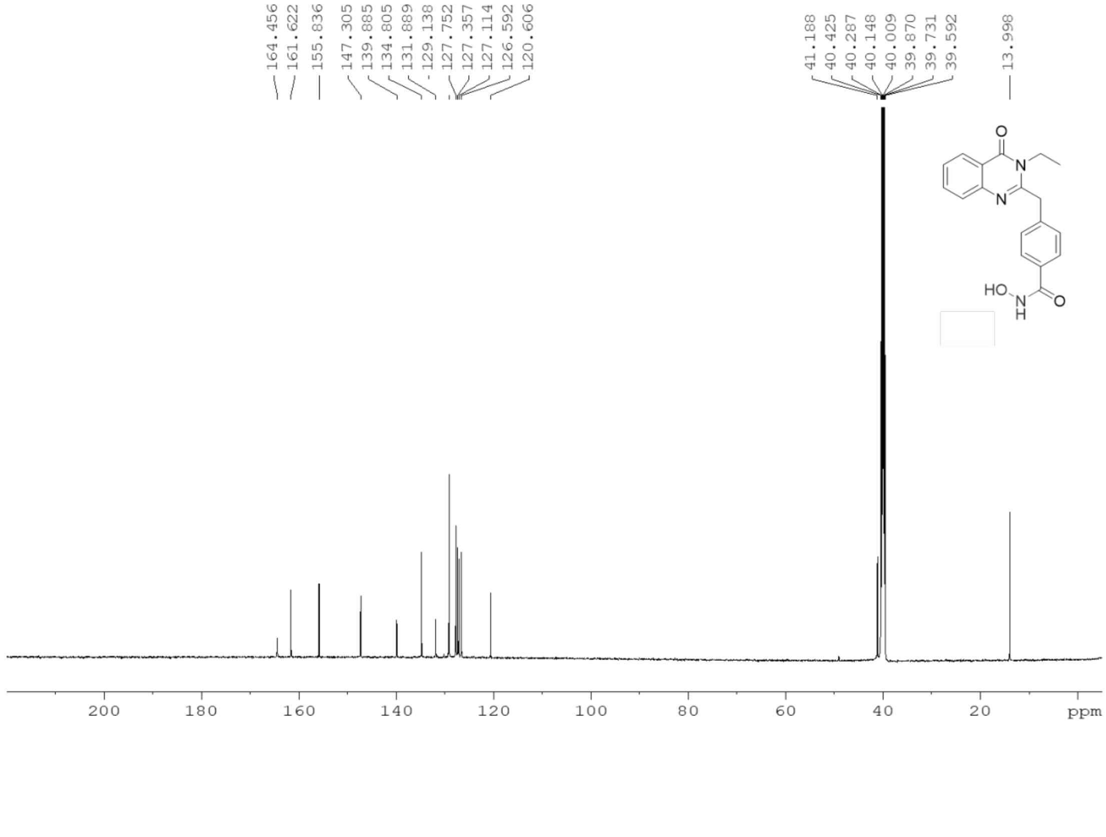

^{1}H NMR spectrum of compound **6c**

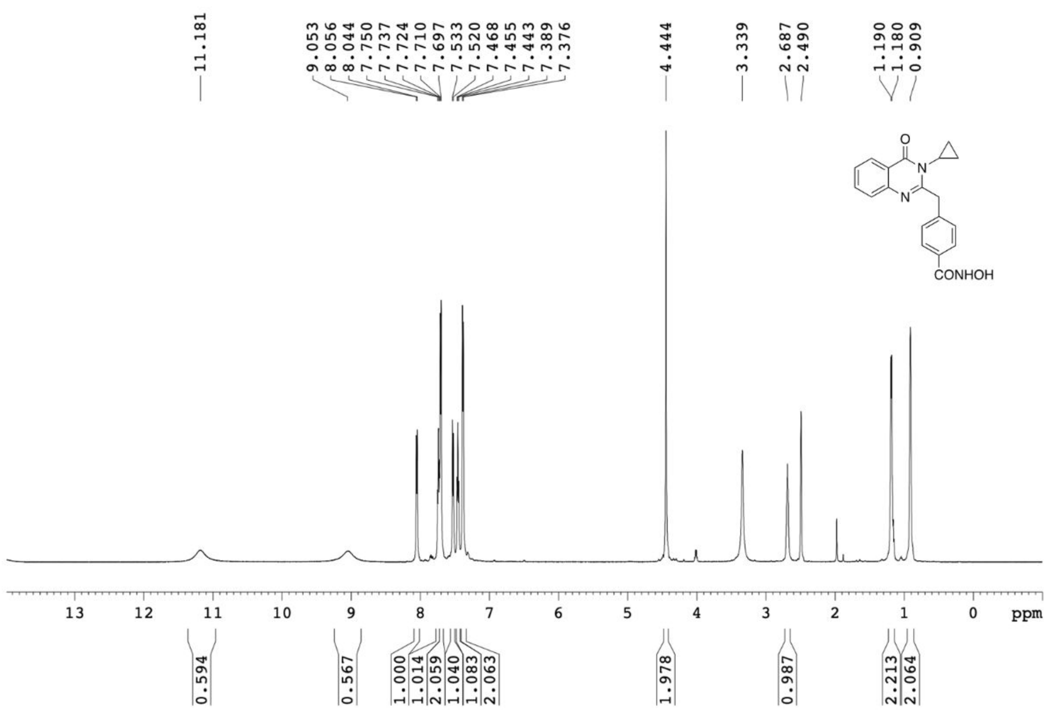

^{13}C NMR spectrum of compound **6c**

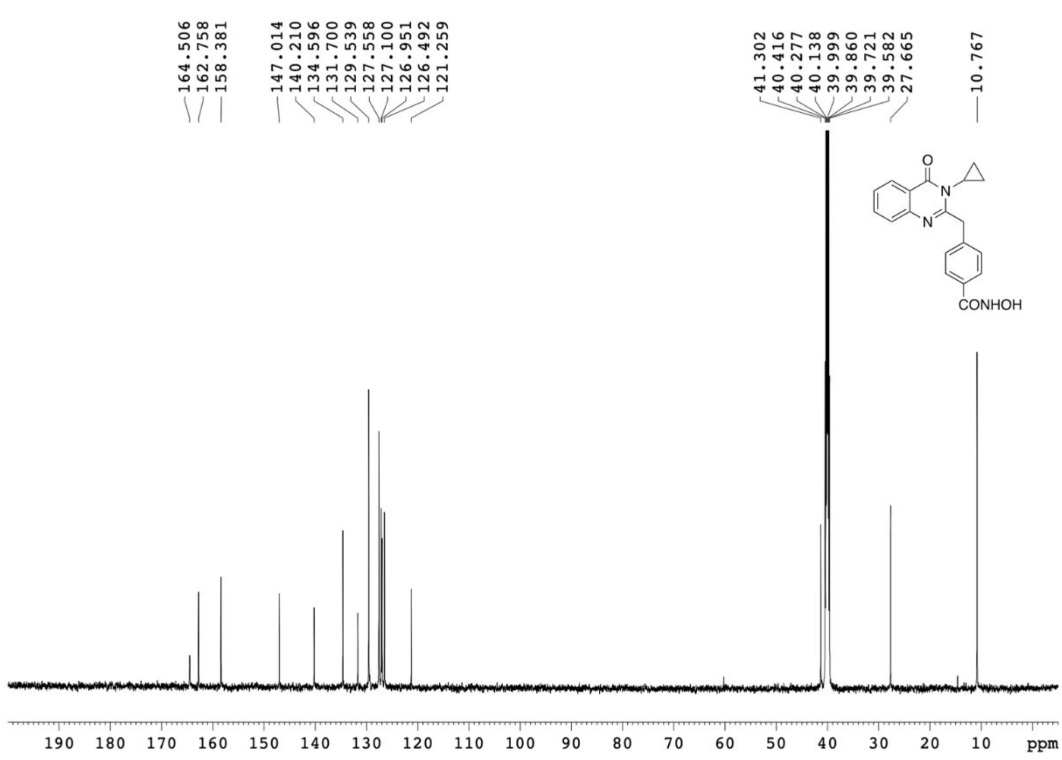

^{1}H NMR spectrum of compound **6d**

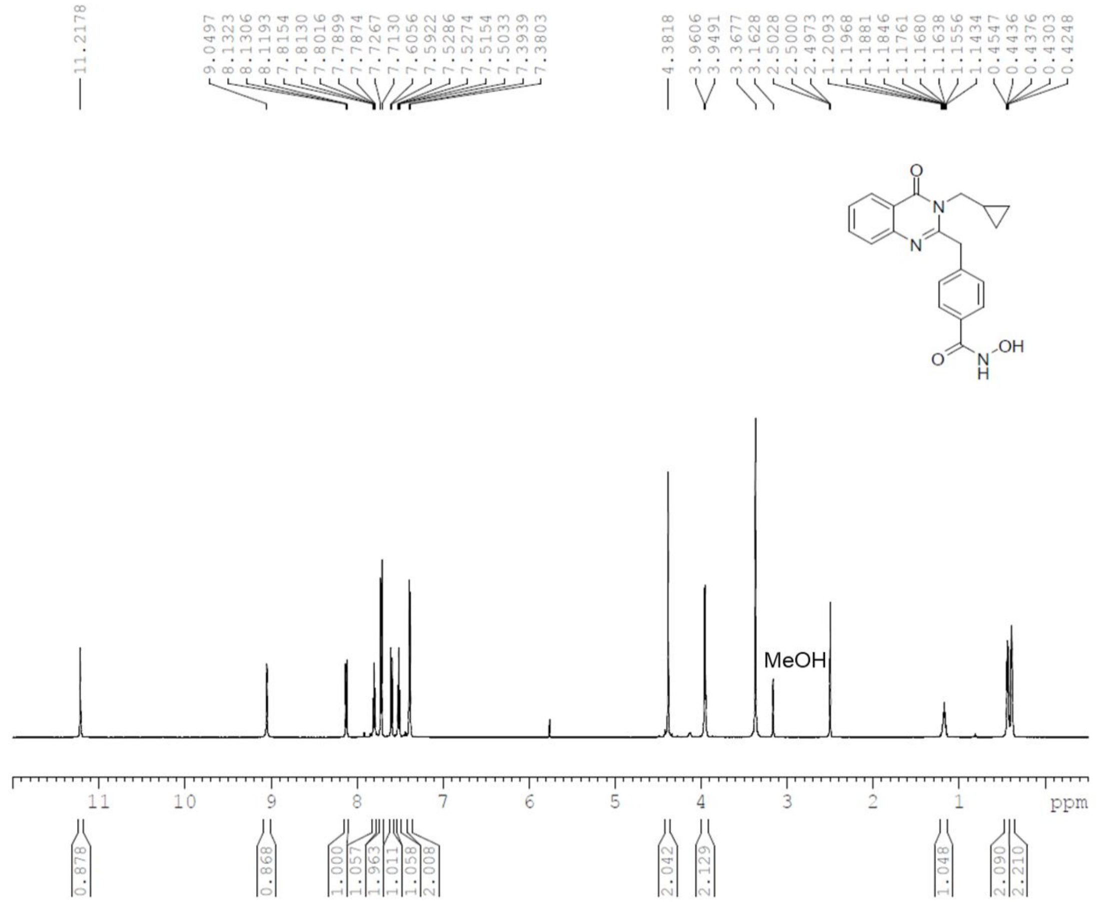

^{13}C NMR spectrum of compound **6d**

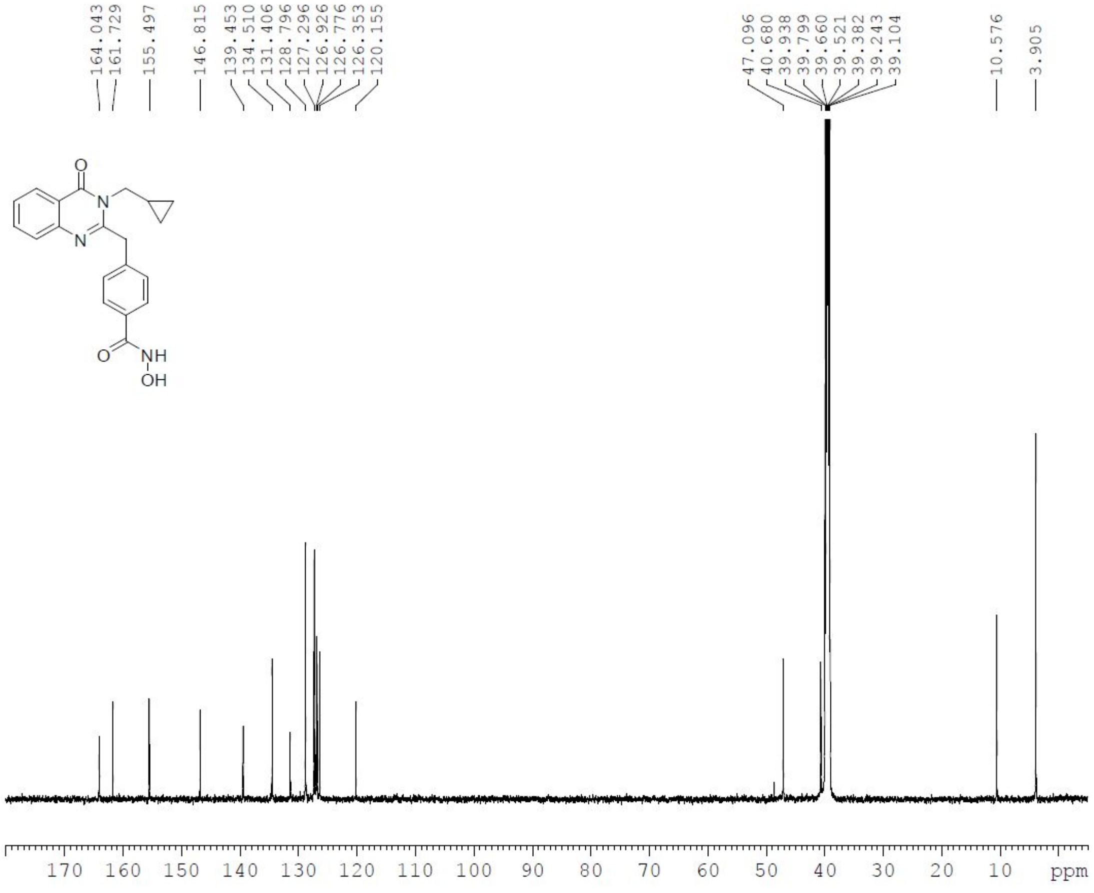

^{1}H NMR spectrum of compound **6e**

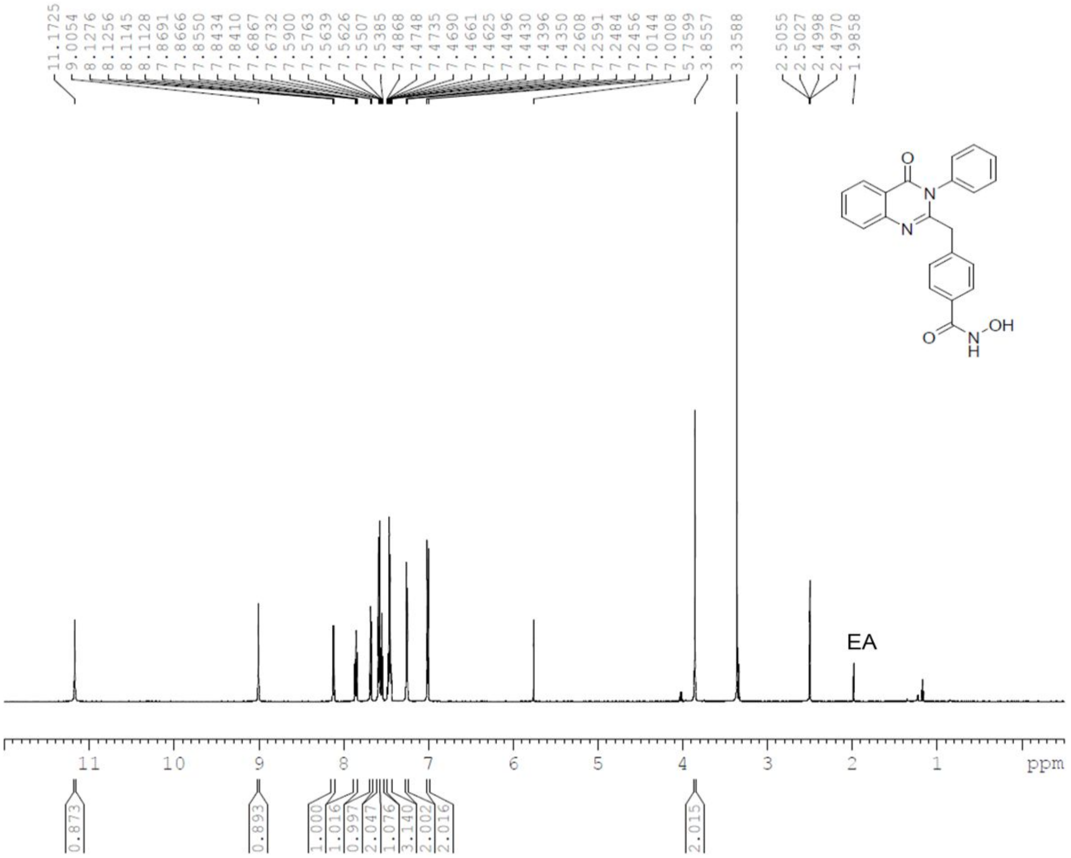

^{13}C NMR spectrum of compound **6e**

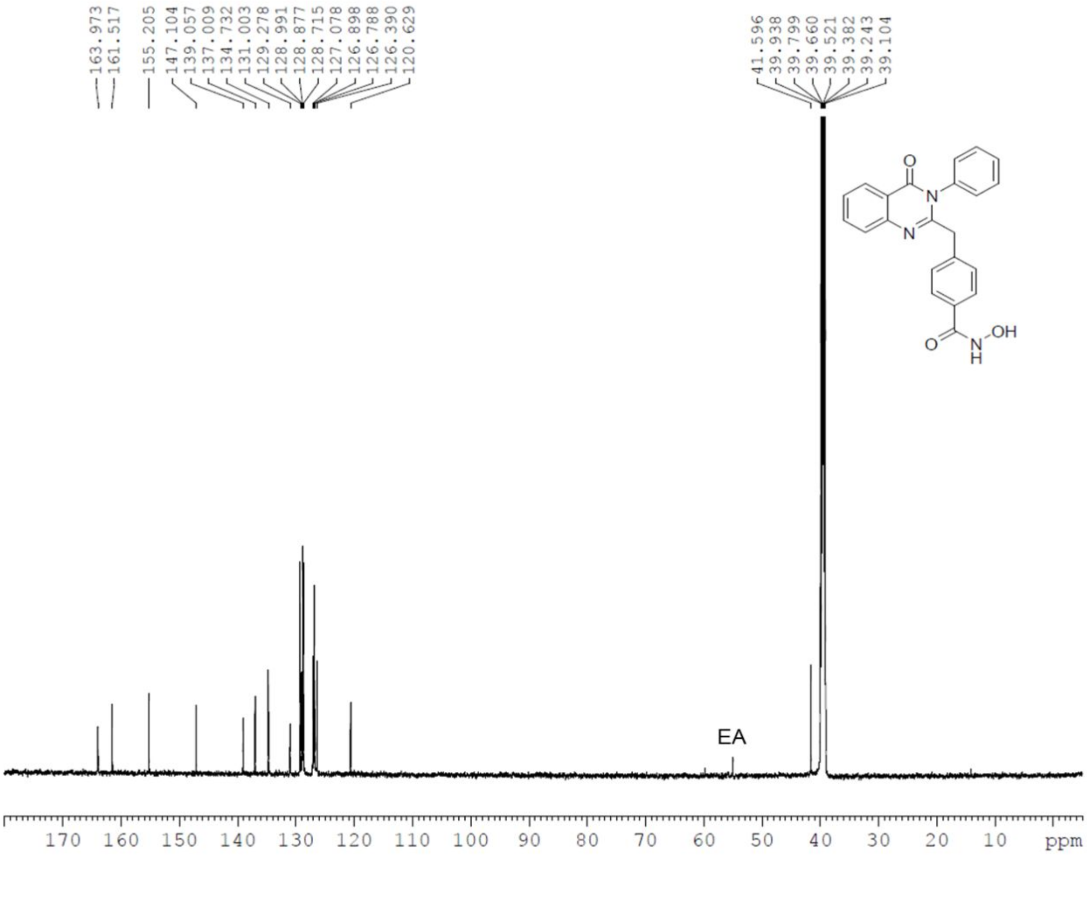

^{1}H NMR spectrum of compound **6f**

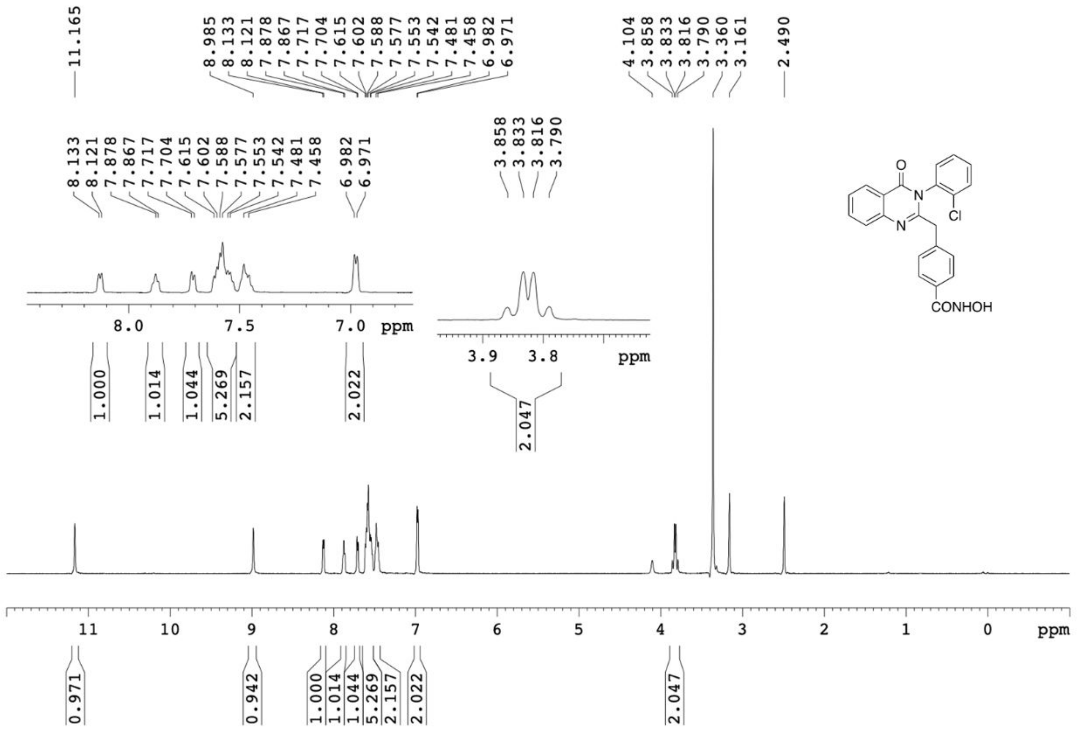

^{13}C NMR spectrum of compound **6f**

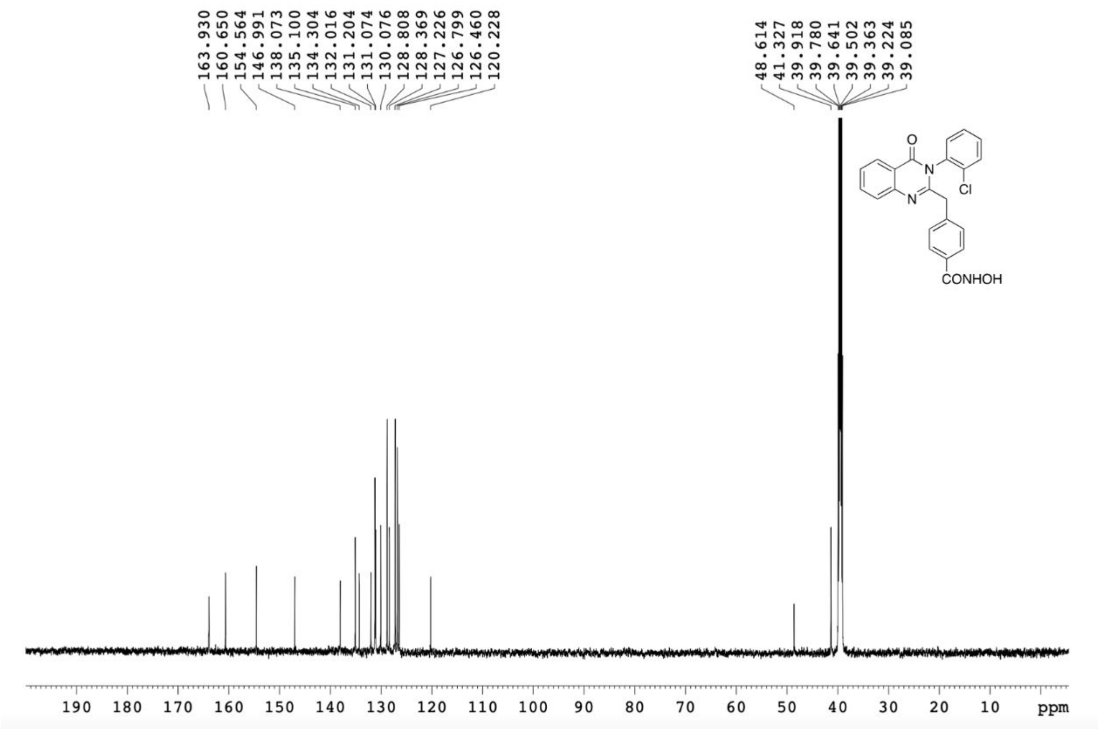

^{1}H NMR spectrum of compound **6g**

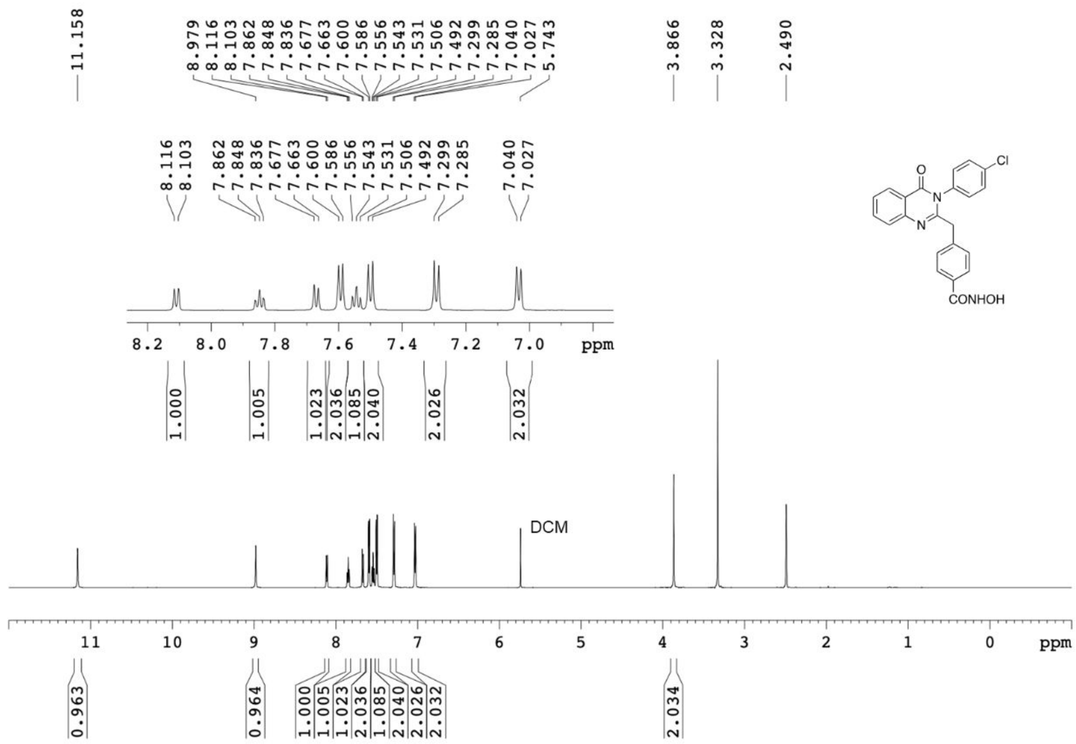

^{13}C NMR spectrum of compound **6g**

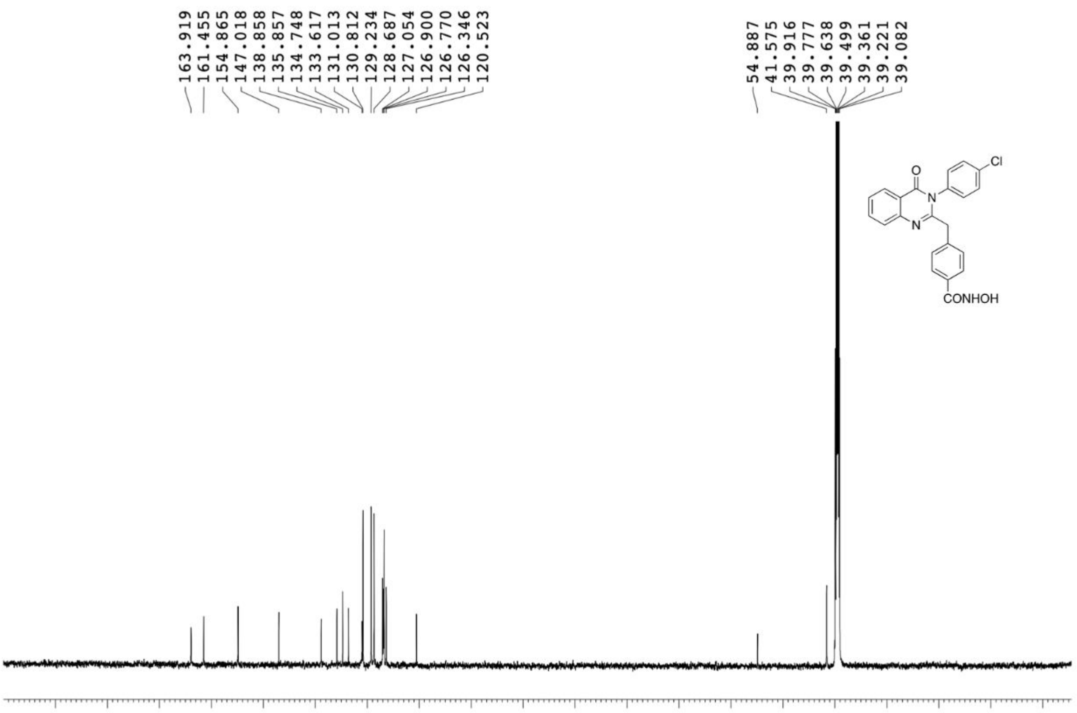

^{1}H NMR spectrum of compound **6h**

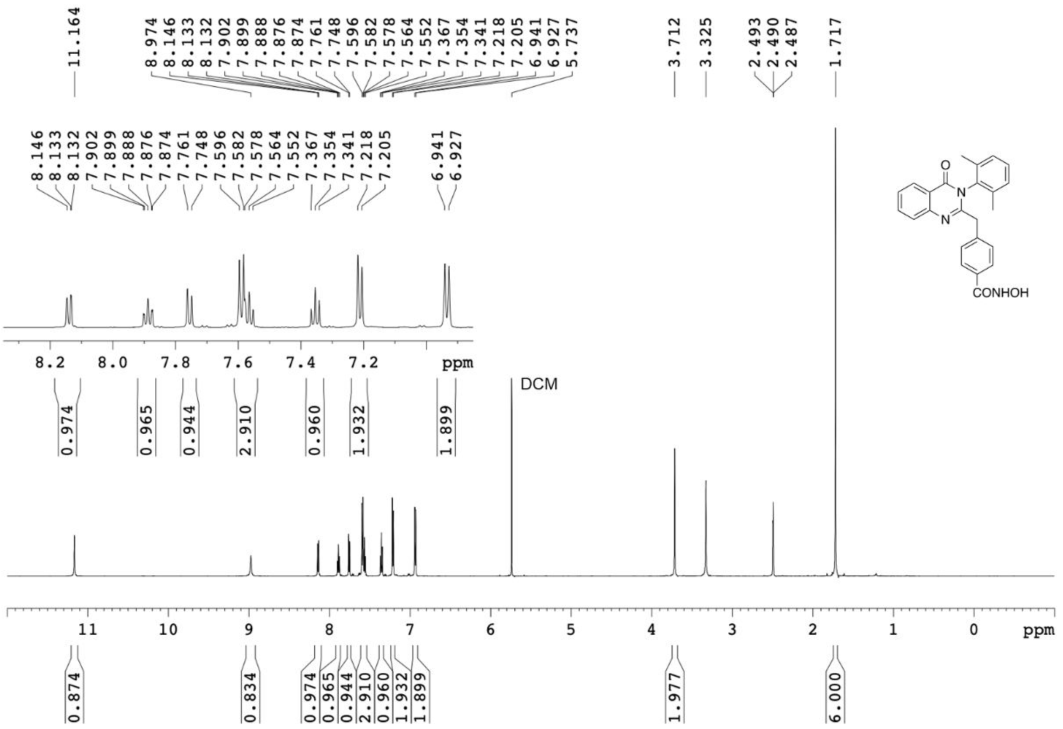

^{13}C NMR spectrum of compound **6h**

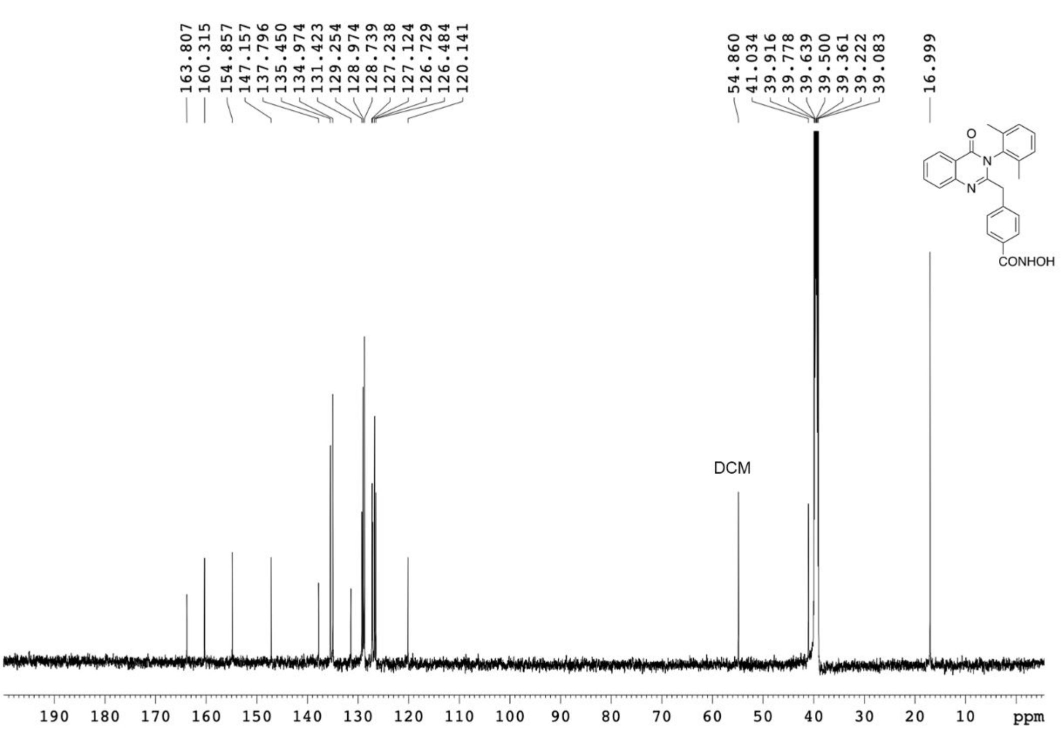

^{1}H NMR spectrum of compound **6i**

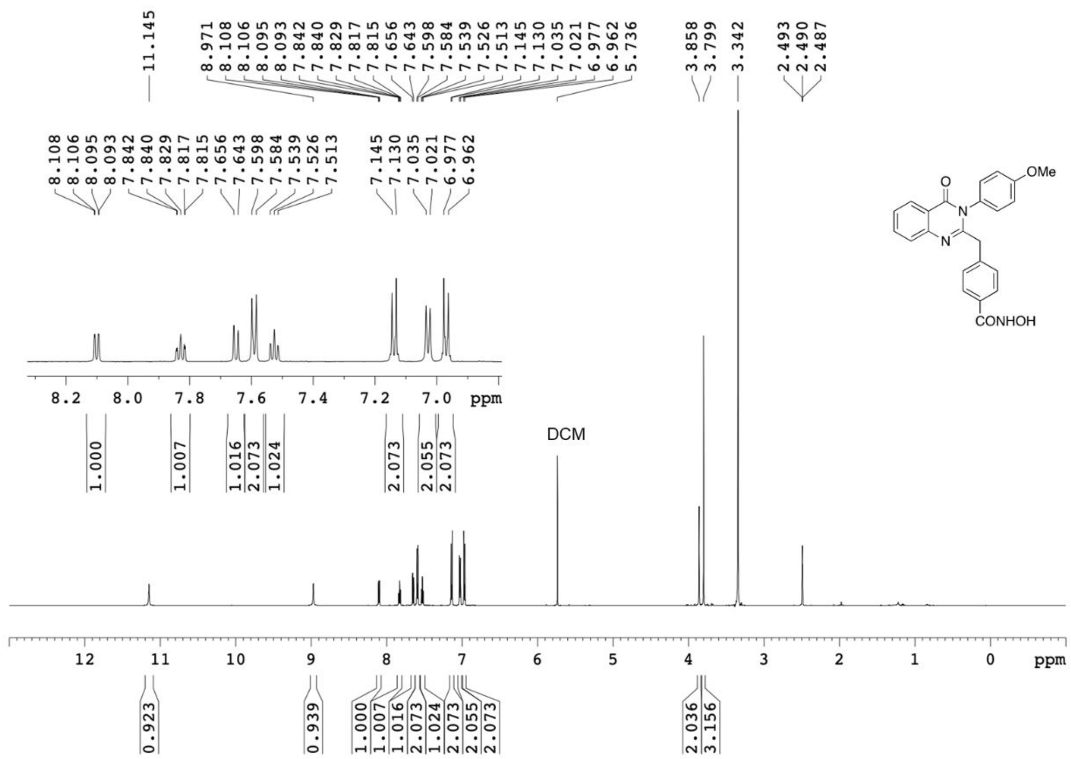

^{13}C NMR spectrum of compound **6i**

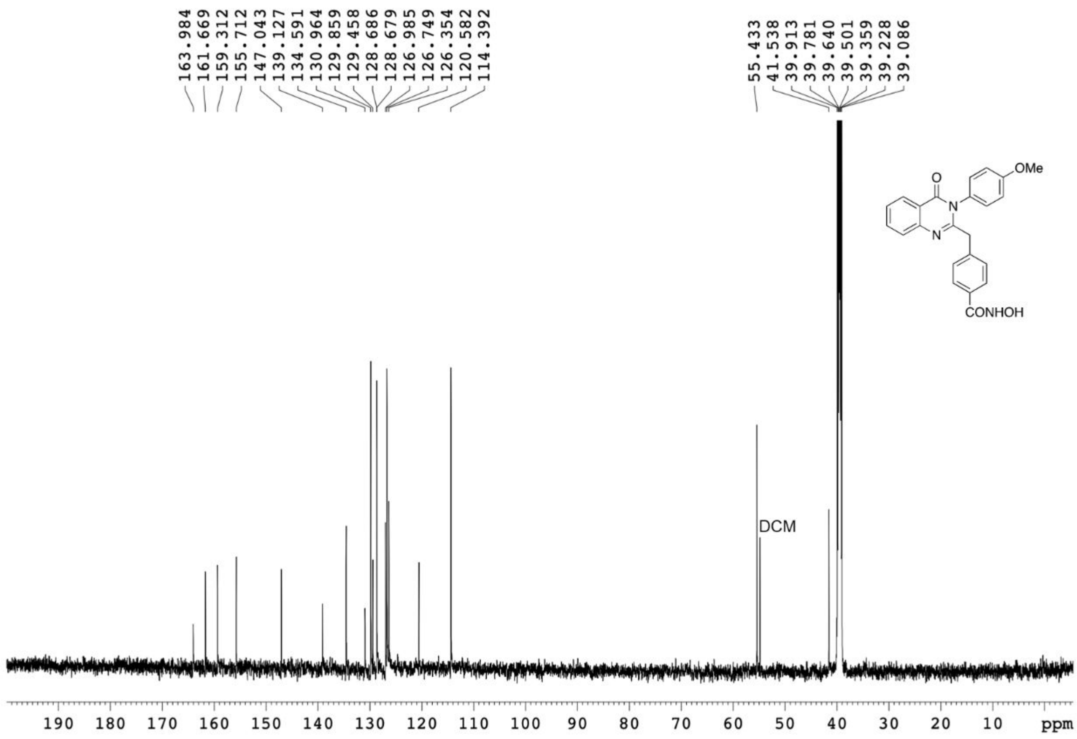

^{1}H NMR spectrum of compound **6j**

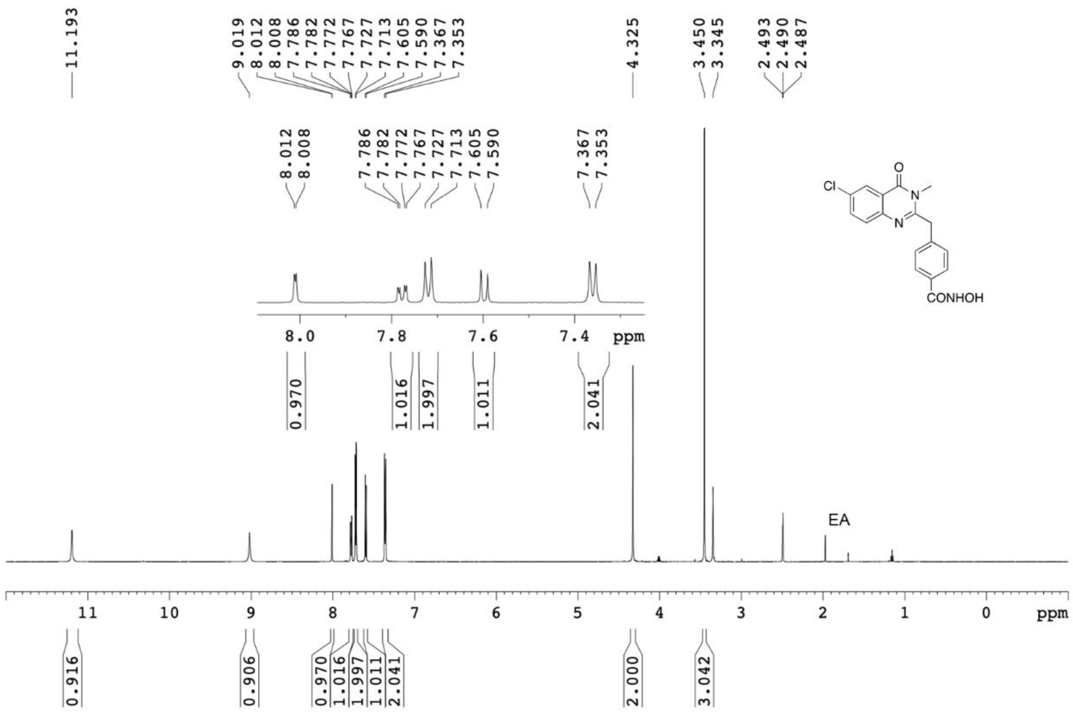

^{13}C NMR spectrum of compound **6j**

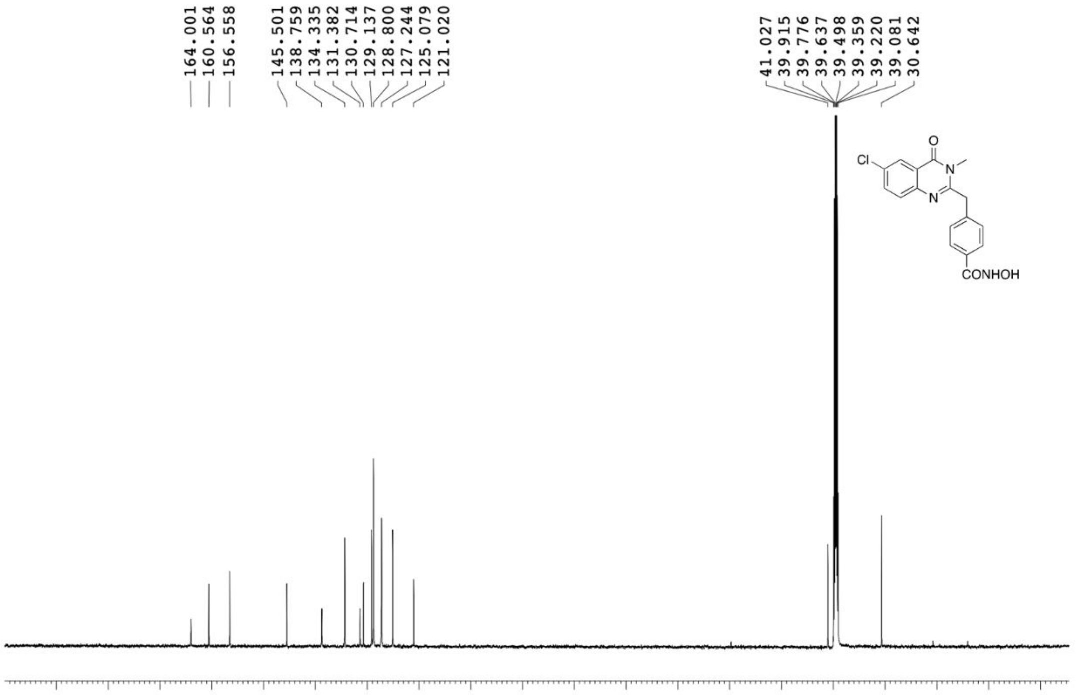

^{1}H NMR spectrum of compound **6k**

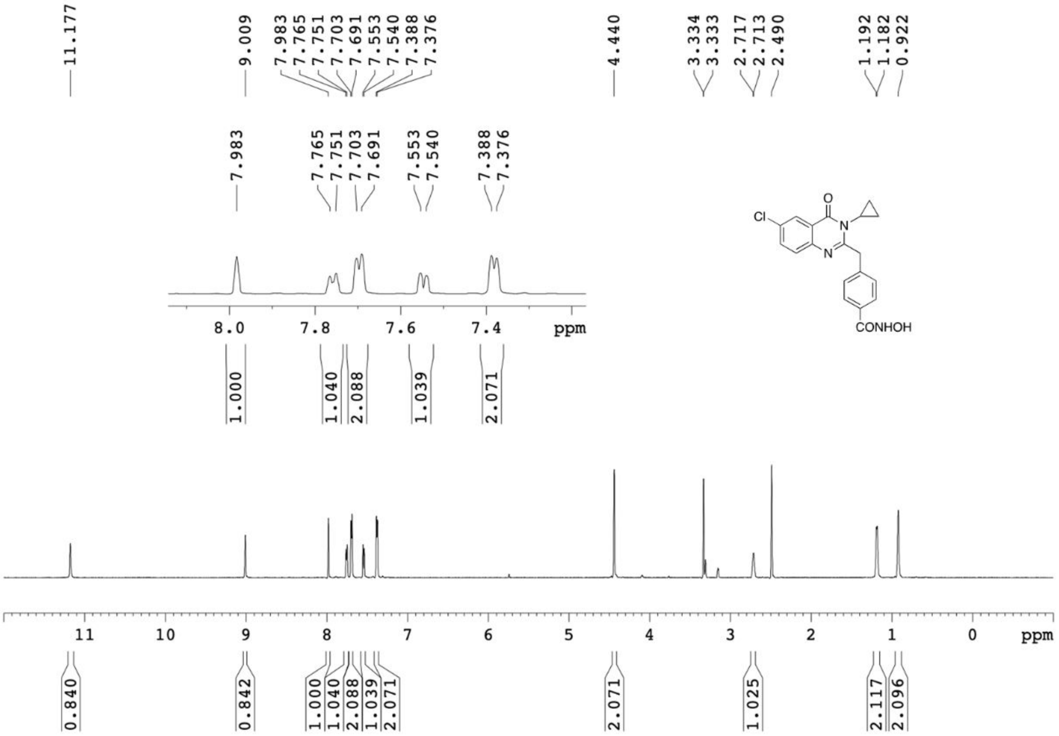

^{13}C NMR spectrum of compound **6k**

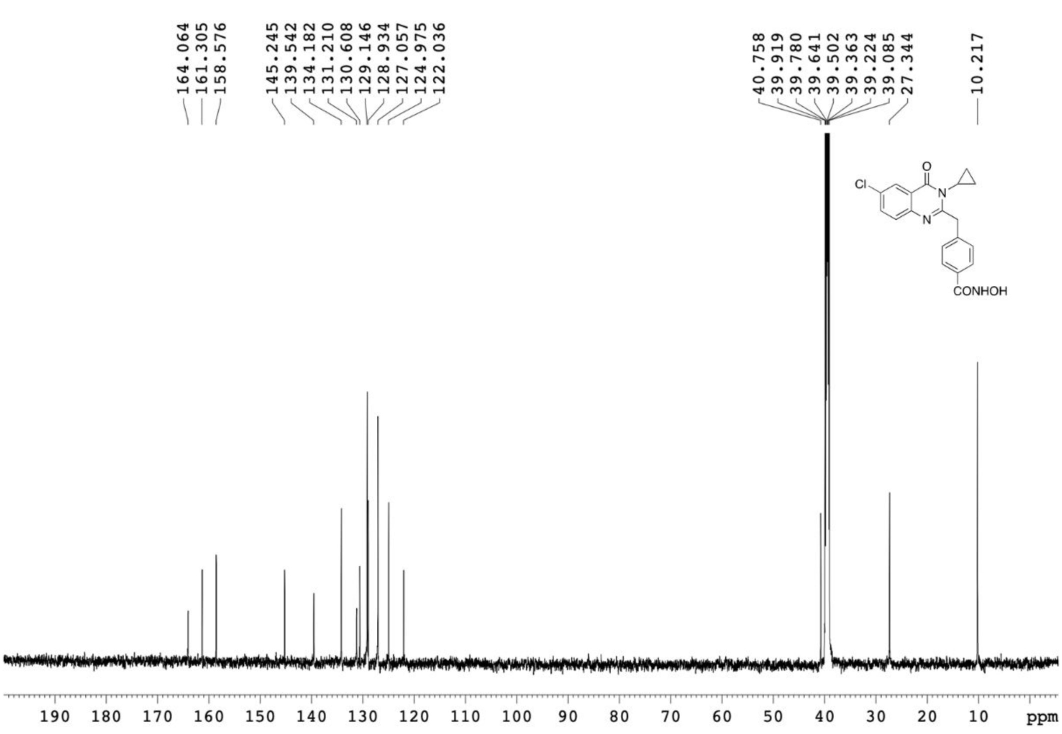

^{1}H NMR spectrum of compound **6l**

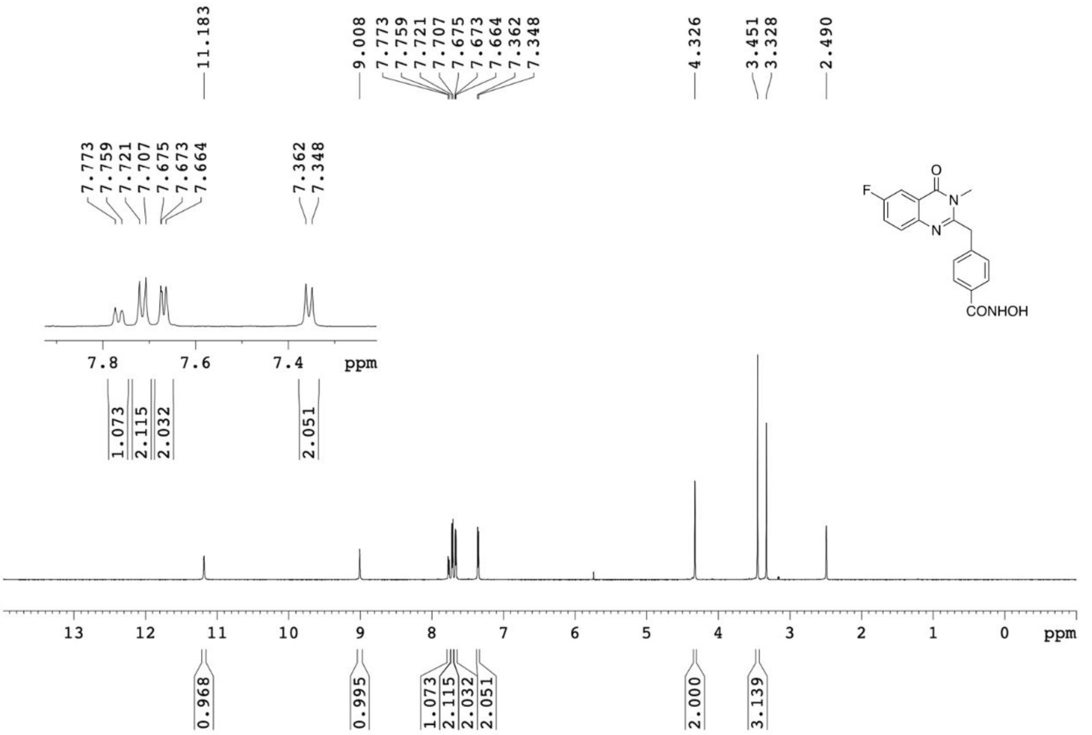

^{13}C NMR spectrum of compound **6l**

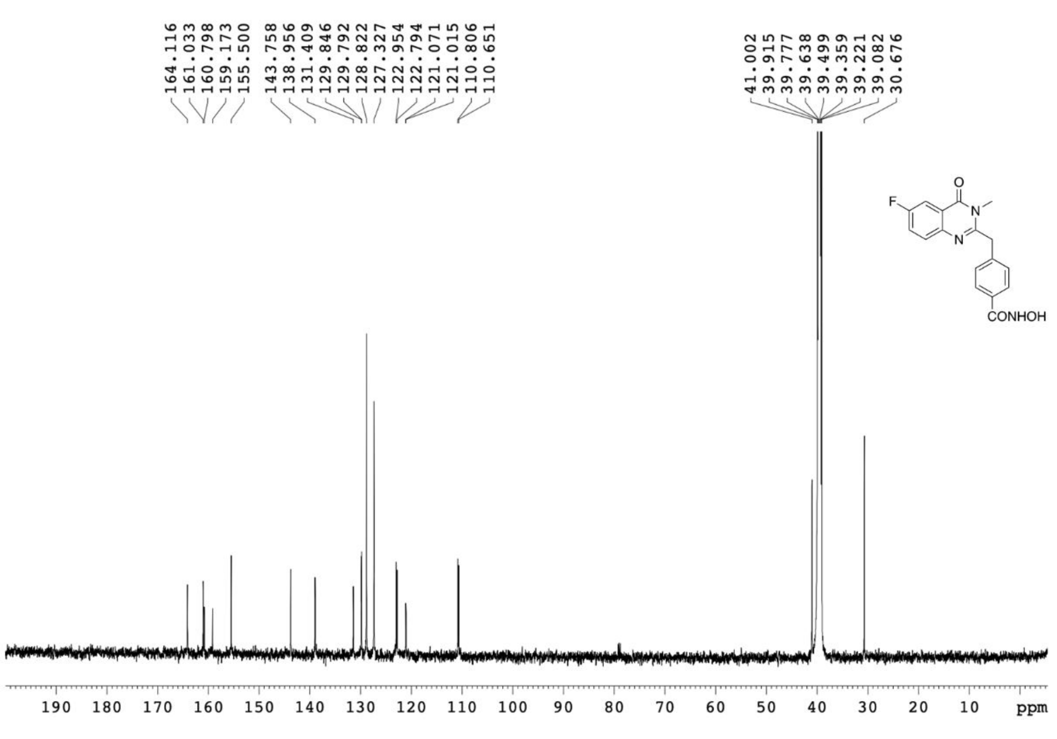

^{1}H NMR spectrum of compound **6m**

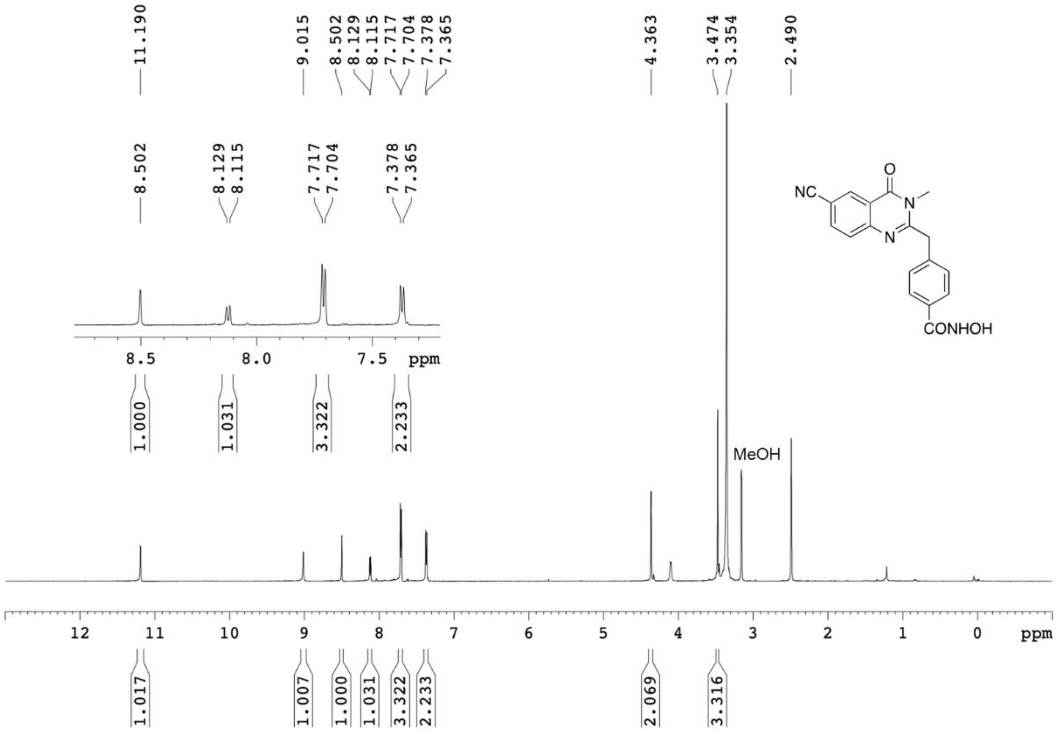

^{13}C NMR spectrum of compound **6m**

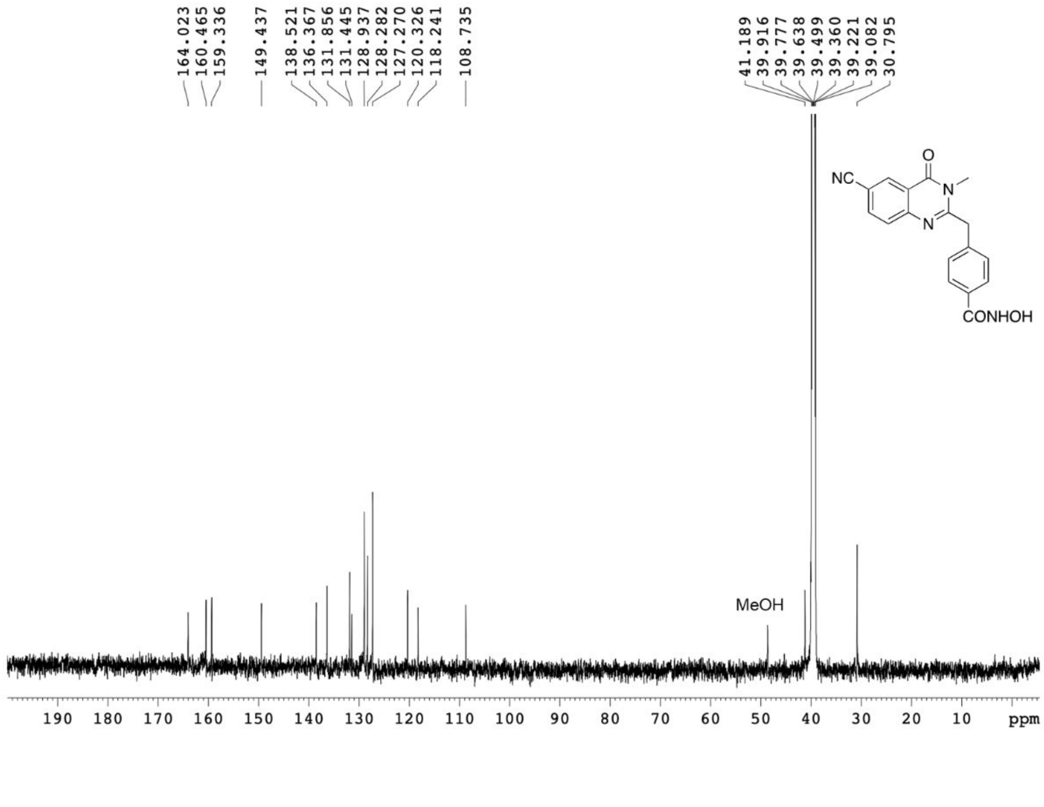

HPLC chromatogram of compound **6a**

<Chromatogram>

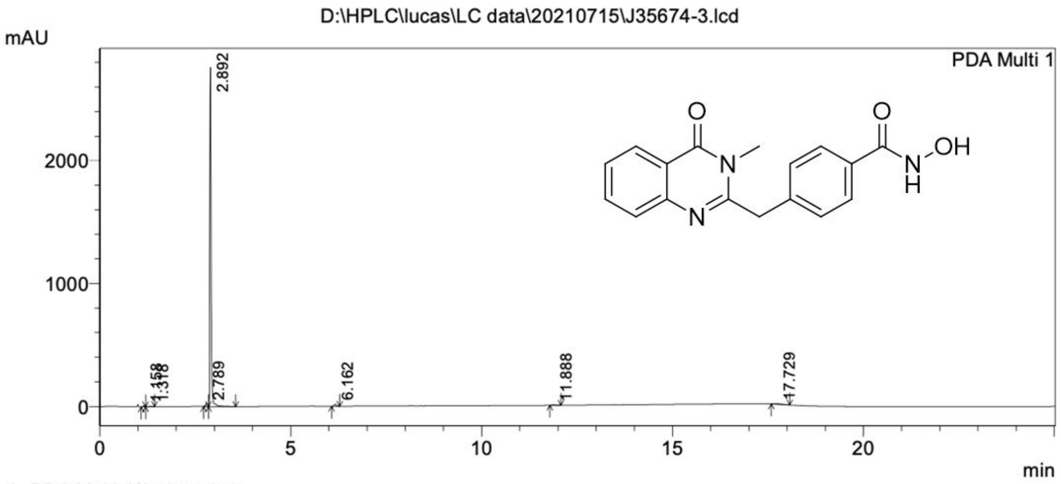

1 PDA Multi 1/254nm 4nm

<Peak List>

PeakTable

PDA Ch1 254nm 4nm

Peak#	Ret. Time	Area	Height	Area %	Height %
1	1.158	35363	12325	0.524	0.436
2	1.318	23376	2703	0.346	0.096
3	2.789	75222	28312	1.115	1.001
4	2.892	6468722	2759759	95.885	97.564
5	6.162	57705	16235	0.855	0.574
6	11.888	22146	5486	0.328	0.194
7	17.729	63785	3854	0.945	0.136
Total		6746319	2828675	100.000	100.000

HPLC chromatogram of compound **6b**

<Chromatogram>

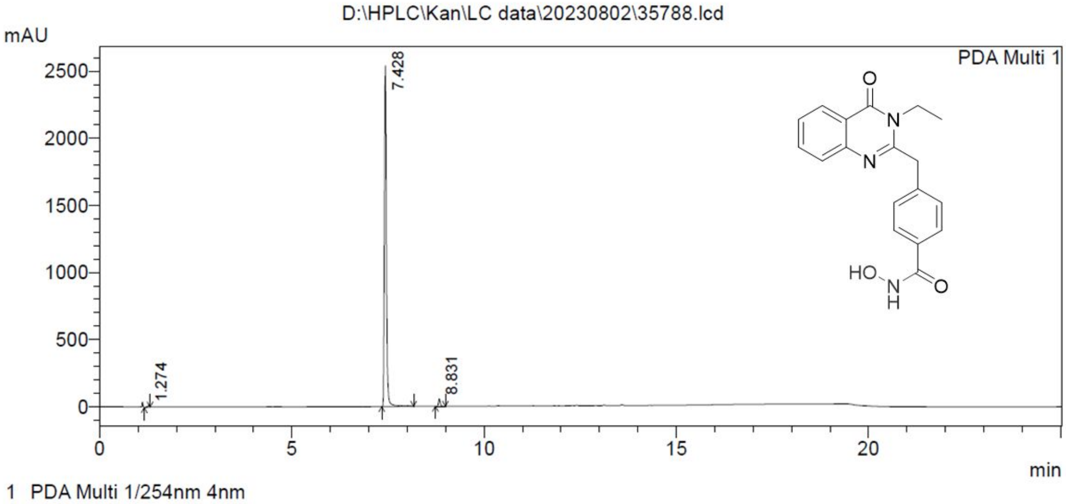

1 PDA Multi 1/254nm 4nm

<Peak List>

PeakTable

PDA Ch1 254nm 4nm

Peak#	Ret. Time	Area	Height	Area %	Height %
1	1.274	45329	21534	0.471	0.823
2	7.428	9412011	2540125	97.772	97.074
3	8.831	169148	55019	1.757	2.103
Total		9626488	2616678	100.000	100.000

HPLC chromatogram of compound **6c**

<Chromatogram>

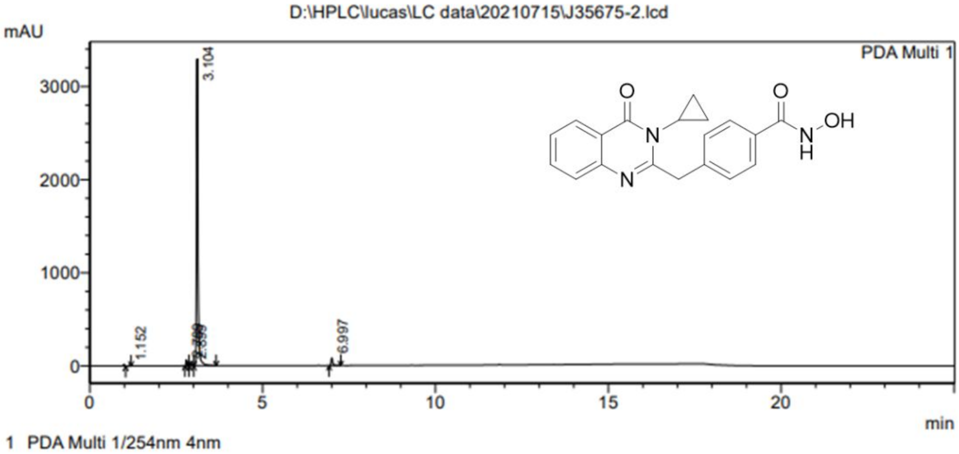

1 PDA Multi 1/254nm 4nm

<Peak List>

PeakTable

PDA Ch1 254nm 4nm

Peak#	Ret. Time	Area	Height	Area %	Height %
1	1.152	34728	10647	0.323	0.306
2	2.789	124835	61543	1.163	1.769
3	2.899	92766	28940	0.864	0.832
4	3.104	10217606	3293058	95.177	94.674
5	6.997	265397	84110	2.472	2.418
Total		10735333	3478298	100.000	100.000

HPLC chromatogram of compound **6d**

<Chromatogram>

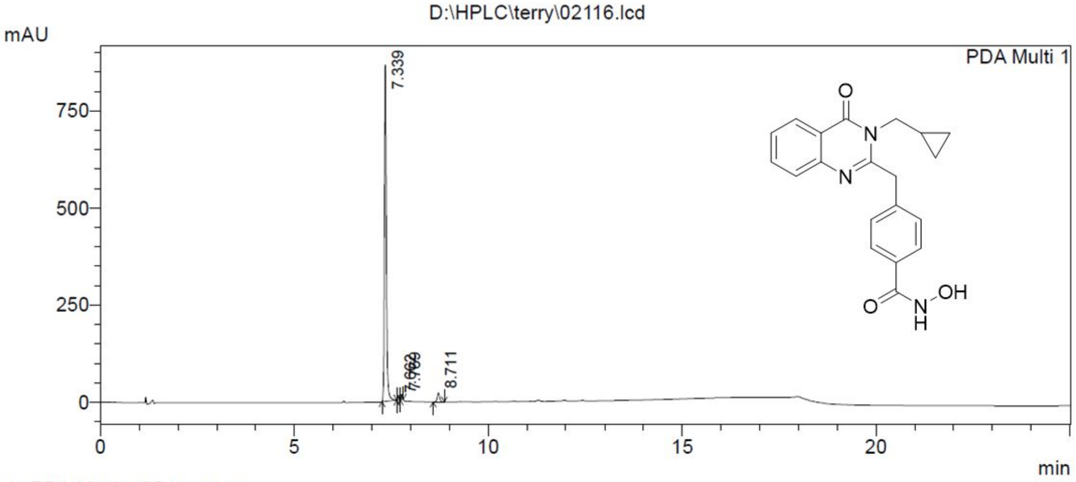

1 PDA Multi 1/254nm 4nm

<Peak List>

PeakTable

PDA Ch1 254nm 4nm

Peak#	Ret. Time	Area	Height	Area %	Height %
1	7.339	3064155	861937	95.093	94.901
2	7.662	25611	8864	0.795	0.976
3	7.769	32888	13845	1.021	1.524
4	8.711	99611	23601	3.091	2.599
Total		3222265	908247	100.000	100.000

HPLC chromatogram of compound **6e**

<Chromatogram>

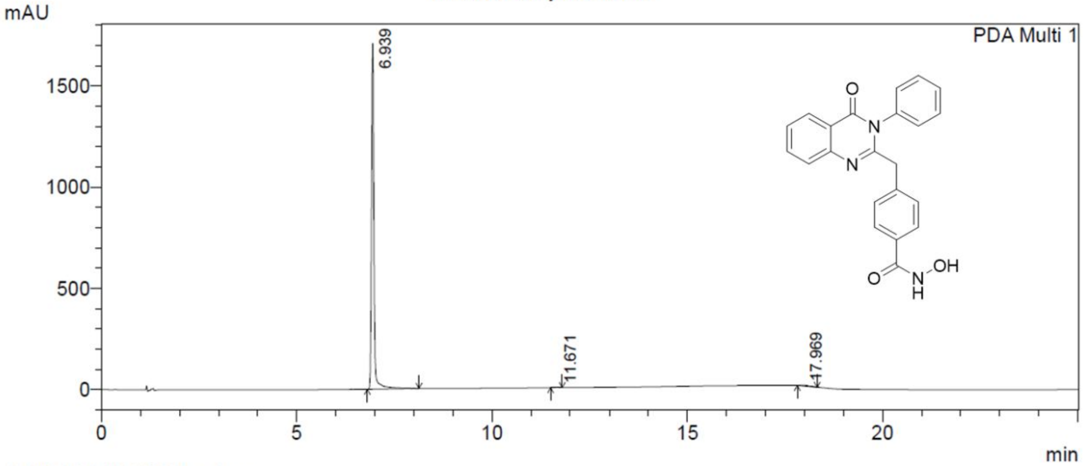

1 PDA Multi 1/254nm 4nm

<Peak List>

PeakTable

PDA Ch1 254nm 4nm

Peak#	Ret. Time	Area	Height	Area %	Height %
1	6.939	7267392	1708877	98.783	99.646
2	11.671	11966	1577	0.163	0.092
3	17.969	77564	4498	1.054	0.262
Total		7356922	1714953	100.000	100.000

HPLC chromatogram of compound **6f**

<Chromatogram>

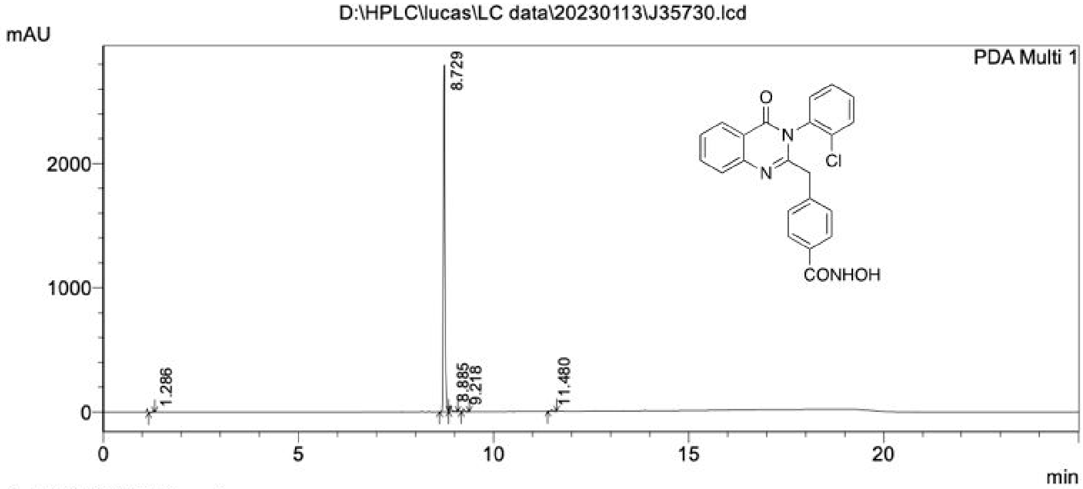

1 PDA Multi 1/254nm 4nm

<Peak List>

PeakTable

PDA Ch1 254nm 4nm

Peak#	Ret. Time	Area	Height	Area %	Height %
1	1.286	34329	12342	0.391	0.428
2	8.729	8477834	2791137	96.598	96.812
3	8.885	165687	46479	1.888	1.612
4	9.218	61246	21164	0.698	0.734
5	11.480	37331	11933	0.425	0.414
Total		8776426	2883054	100.000	100.000

HPLC chromatogram of compound **6g**

<Chromatogram>

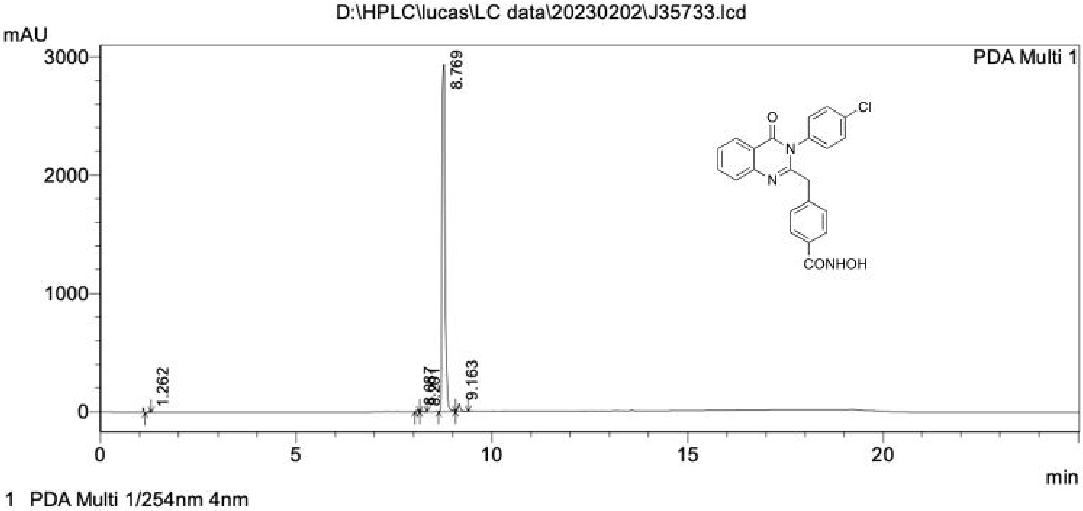

1 PDA Multi 1/254nm 4nm

<Peak List>

PeakTable

PDA Ch1 254nm 4nm

Peak#	Ret. Time	Area	Height	Area %	Height %
1	1.262	47467	13333	0.291	0.439
2	8.087	52795	17844	0.324	0.587
3	8.201	31844	9549	0.195	0.314
4	8.769	15863767	2934227	97.371	96.537
5	9.163	296191	64525	1.818	2.123
Total		16292063	3039477	100.000	100.000

HPLC chromatogram of compound **6h**

<Chromatogram>

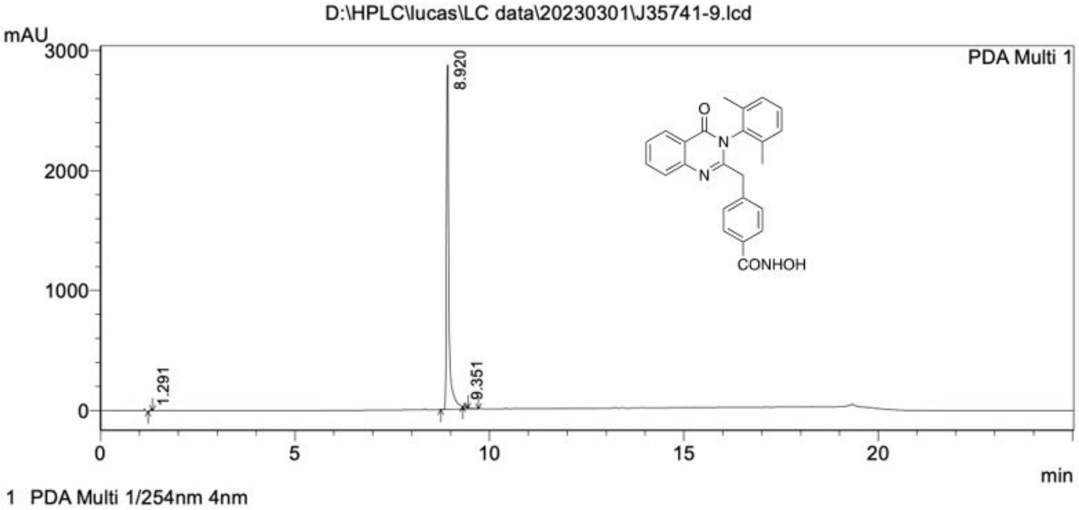

1 PDA Multi 1/254nm 4nm

<Peak List>

PeakTable

PDA Ch1 254nm 4nm

Peak#	Ret. Time	Area	Height	Area %	Height %
1	1.291	33400	12253	0.240	0.421
2	8.920	13808422	2870773	99.275	98.649
3	9.351	67404	27053	0.485	0.930
Total		13909227	2910079	100.000	100.000

HPLC chromatogram of compound **6i**

<Chromatogram>

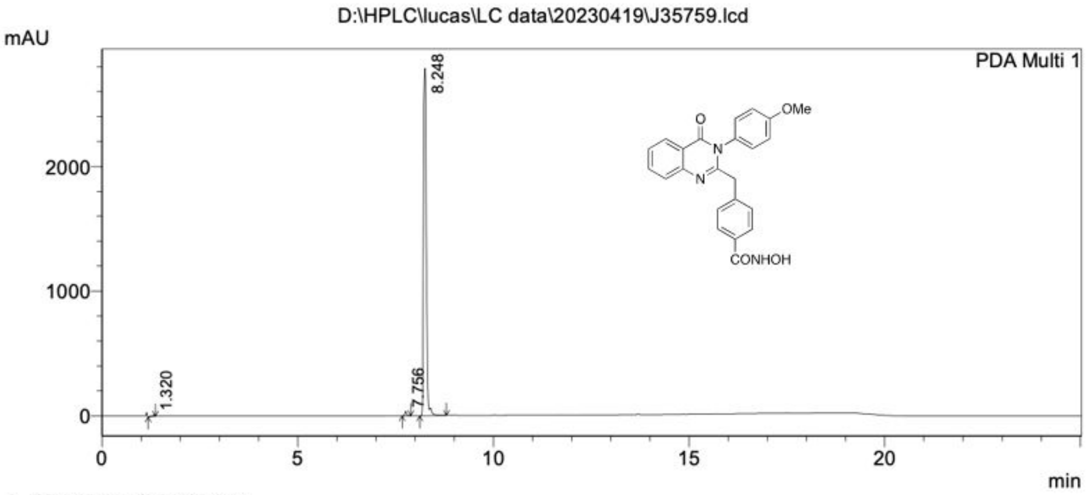

1 PDA Multi 1/254nm 4nm

<Peak List>

PeakTable

PDA Ch1 254nm 4nm

Peak#	Ret. Time	Area	Height	Area %	Height %
1	1.320	45583	14718	0.313	0.519
2	7.756	106524	35806	0.732	1.263
3	8.248	14399346	2783884	98.955	98.217
Total		14551453	2834408	100.000	100.000

HPLC chromatogram of compound **6j**

<Chromatogram>

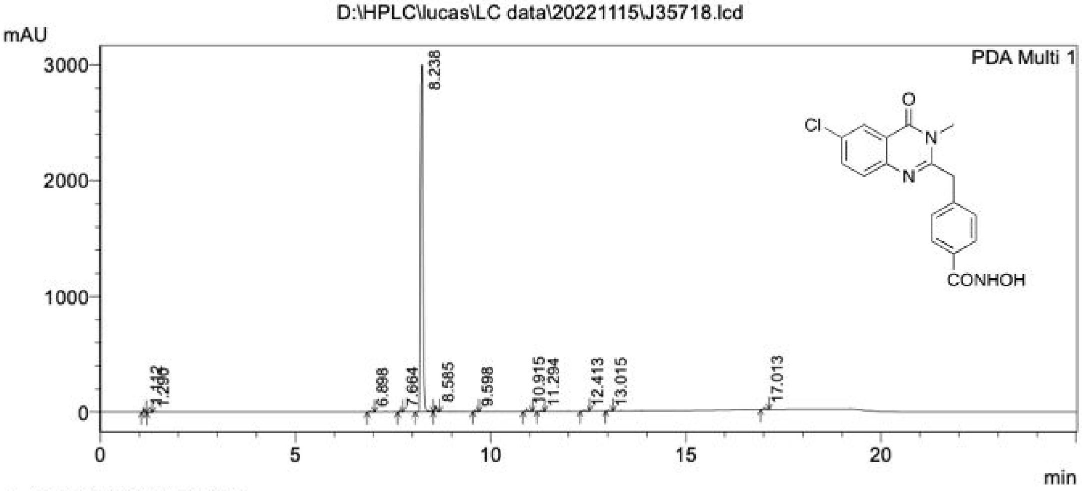

1 PDA Multi 1/254nm 4nm

<Peak List>

PeakTable

PDA Ch1 254nm 4nm

Peak#	Ret. Time	Area	Height	Area %	Height %
1	1.112	63110	35522	0.459	1.107
2	1.290	36506	9023	0.266	0.281
3	6.898	34325	11455	0.250	0.357
4	7.664	40487	14320	0.295	0.446
5	8.238	13126752	2998194	95.490	93.464
6	8.585	150837	53674	1.097	1.673
7	9.598	40347	13949	0.294	0.435
8	10.915	82506	26171	0.600	0.816
9	11.294	39246	11076	0.285	0.345
10	12.413	47115	9368	0.343	0.292
11	13.015	32370	8996	0.235	0.280
12	17.013	53152	16099	0.387	0.502
Total		13746754	3207847	100.000	100.000

HPLC chromatogram of compound **6k**

<Chromatogram>

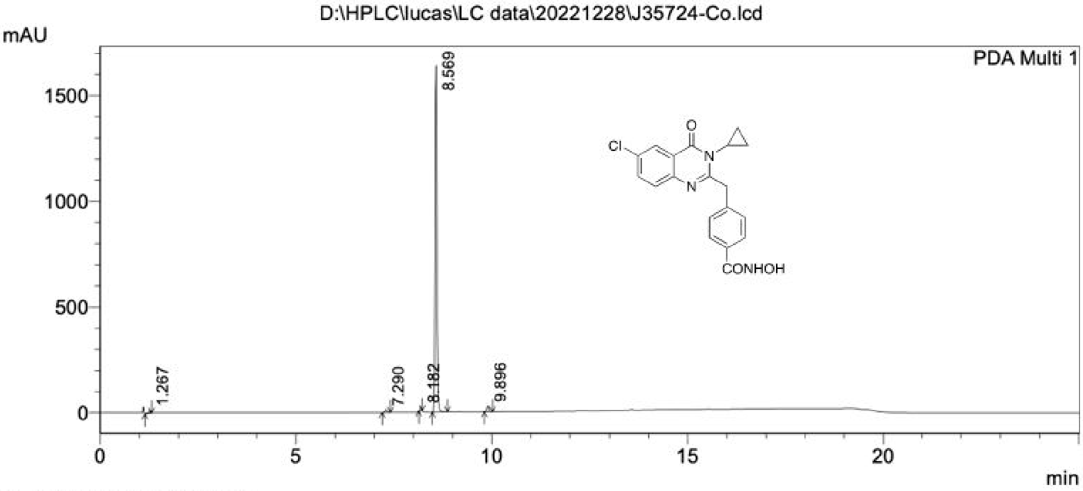

1 PDA Multi 1/254nm 4nm

<Peak List>

PeakTable

PDA Ch1 254nm 4nm

Peak#	Ret. Time	Area	Height	Area %	Height %
1	1.267	47745	15320	0.902	0.894
2	7.290	43341	13811	0.819	0.806
3	8.182	50739	18614	0.959	1.087
4	8.569	5055719	1637214	95.562	95.588
5	9.896	92973	27819	1.757	1.624
Total		5290518	1712778	100.000	100.000

HPLC chromatogram of compound **6l**

<Chromatogram>

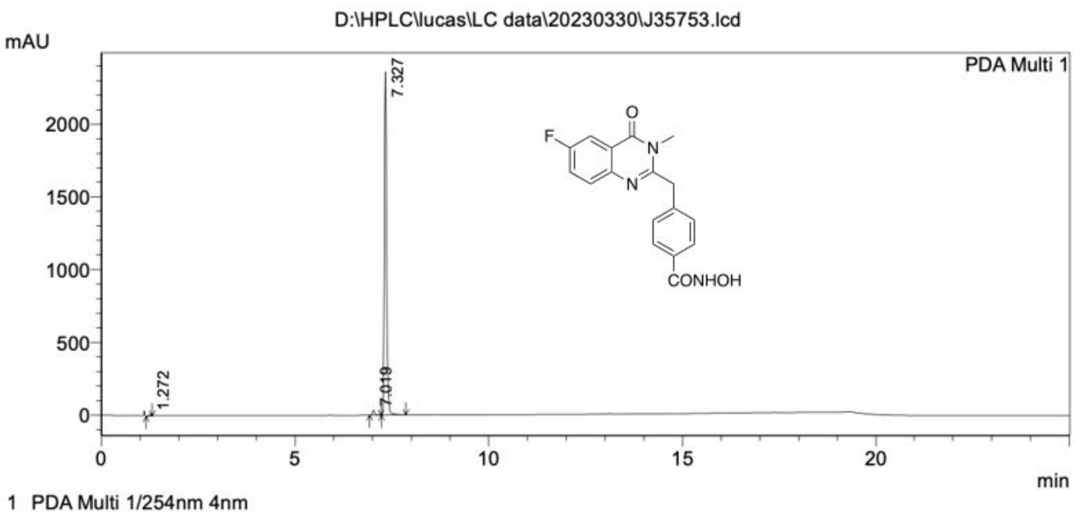

1 PDA Multi 1/254nm 4nm

<Peak List>

PeakTable

PDA Ch1 254nm 4nm

Peak#	Ret. Time	Area	Height	Area %	Height %
1	1.272	48982	14191	0.511	0.590
2	7.019	142709	33805	1.488	1.405
3	7.327	9402034	2358150	98.002	98.005
Total		9593725	2406145	100.000	100.000

HPLC chromatogram of compound **6m**

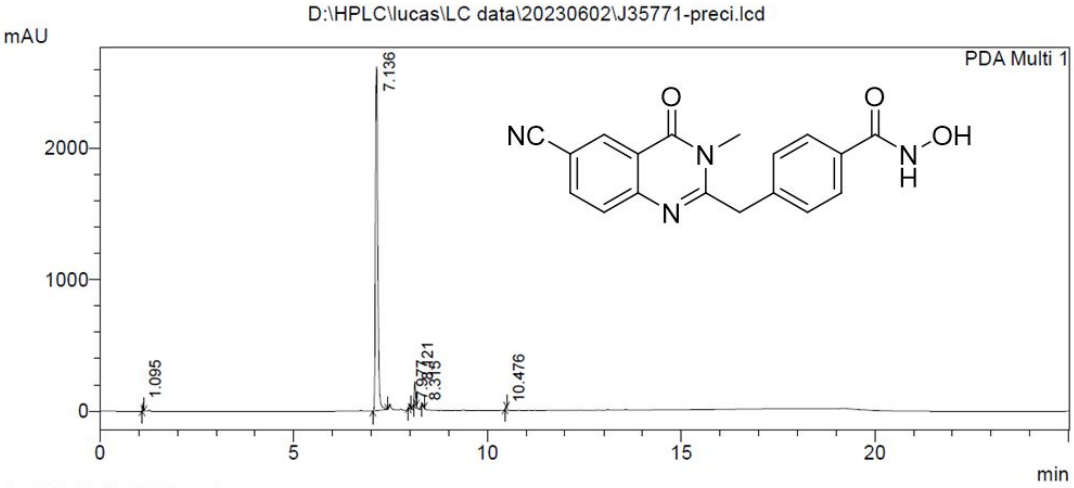

<Peak List>

PeakTable

PDA Ch1 254nm 4nm

Peak#	Ret. Time	Area	Height	Area %	Height %
1	1.095	67808	61284	0.574	2.111
2	7.136	11243312	2615474	95.198	90.108
3	7.977	72137	32014	0.611	1.103
4	8.121	396262	171177	3.355	5.897
5	8.315	5753	8739	0.049	0.301
6	10.476	25161	13916	0.213	0.479
Total		11810432	2902604	100.000	100.000
